# Mosasauroid phylogeny under multiple phylogenetic methods provides new insights on the evolution of aquatic adaptations in the group

**DOI:** 10.1371/journal.pone.0176773

**Published:** 2017-05-03

**Authors:** Tiago R. Simões, Oksana Vernygora, Ilaria Paparella, Paulina Jimenez-Huidobro, Michael W. Caldwell

**Affiliations:** 1 Department of Biological Sciences, University of Alberta, Edmonton, Alberta, Canada; 2 Department of Earth and Atmospheric Sciences, University of Alberta, Edmonton, Alberta, Canada; Royal Belgian Institute of Natural Sciences, BELGIUM

## Abstract

Mosasauroids were a successful lineage of squamate reptiles (lizards and snakes) that radiated during the Late Cretaceous (95–66 million years ago). They can be considered one of the few lineages in the evolutionary history of tetrapods to have acquired a fully aquatic lifestyle, similarly to whales, ichthyosaurs and plesiosaurs. Despite a long history of research on this group, their phylogenetic relationships have only been tested so far using traditional (unweighted) maximum parsimony. However, hypotheses of mosasauroid relationships and the recently proposed multiple origins of aquatically adapted pelvic and pedal features in this group can be more thoroughly tested by methods that take into account variation in branch lengths and evolutionary rates. In this study, we present the first mosasauroid phylogenetic analysis performed under different analytical methods, including maximum likelihood, Bayesian inference, and implied weighting maximum parsimony. The results indicate a lack of congruence in the topological position of halisaurines and *Dallasaurus*. Additionally, the genus *Prognathodon* is paraphyletic under all hypotheses. Interestingly, a number of traditional mosasauroid clades become weakly supported, or unresolved, under Bayesian analyses. The reduced resolutions in some consensus trees create ambiguities concerning the evolution of fully aquatic pelvic/pedal conditions under many analyses. However, when enough resolution was obtained, reversals of the pelvic/pedal conditions were favoured by parsimony and likelihood ancestral state reconstructions instead of independent origins of aquatic features in mosasauroids. It is concluded that most of the observed discrepancies among the results can be associated with different analytical procedures, but also due to limited postcranial data on halisaurines, yaguarasaurines and *Dallasaurus*.

## Introduction

Mosasauroid reptiles *sensu* Bell [1] (mosasaurids + aigialosaurids) were a diverse and globally distributed clade of lizards that invaded freshwater and marine environments during the Late Cretaceous [1–5]. Although multiple reptilian clades have become secondarily adapted to aquatic habitats, mosasauroids were one of the few to become fully aquatic—feeding and spending most of their life cycle in aquatic environments [6]. Some of the most relevant aspects of mosasauroid morphology that illustrate their transition to an aquatic lifestyle are concentrated in a set of changes in their pelvic and pedal anatomy. These changes, such as loss of contact between the sacral vertebrae and the pelvis followed by a reduction in the number of sacrals, characterize the so called hydropelvic condition [7]. Additionally, the development of hyperphalangy in the autopodium, which aids in locomotion under water, constitutes the hydropedal condition [8]. These two conditions of the pelvic and pedal morphologies as observed in most mosasauroids contrast to the connection between sacrum and ilium (termed plesiopelvic), as well as the typical phalangeal formula (plesiopedal), as seen in most limbed squamates [7, 8].

Despite numerous previous studies on mosasauroid phylogeny and evolution of pelvic and pedal characters, it is still uncertain whether mosasauroids acquired their aquatic adaptations only once in their evolutionary history [1, 9, 10], or multiple times [7, 8, 11, 12]. The hypothesis of convergent evolution of aquatic adaptations in mosasauroids has been proposed, and given further support in the past decade, due to the incorporation of new taxa (e.g. *Dallasaurus* and *Tethysaurus*) into phylogenetic analyses of mosasauroids. However, some other studies (with a similar taxonomic sampling) still recover fully aquatic mosasaurs as forming a single clade [11, 13]—e.g. the clade Natantia of Bell [1], also recovered by Caldwell [9, 10].

One common aspect to all analyses published so far is that these have been analyzed using only traditional unweighted maximum parsimony. Nevertheless, incorporating multiple methods that take into account the effect of highly plastic characters to phylogenetic inference can provide an important additional test towards hypothesis of mosasauroid interrelationships, and of the potentially homoplastic origin of fully aquatic forms. In the present study, we provide the first analysis of mosasauroid relationships based on traditional (unweighted) maximum parsimony using two different coding schemes: contingent (Co-UMP) and multistate codings (Mu-UMP). Additionally, we utilize methods designed to downweight homoplasy and/or take evolutionary rates along with branch lengths into consideration: parsimony under implied weighting (IWMP), maximum likelihood (ML) and Bayesian inference. The latter methods should provide a more robust phylogenetic assessment of the recently proposed convergent evolution of aquatically adapted features than the traditional maximum parsimony. We also make comments and considerations relative to the benefits and limitations of likelihood methods in phylogenetic investigations using morphological data, and their potential application to the study of fossil lineages.

## Materials and Methods

### Important considerations on the usage of likelihood based methods for the analysis of morphological data

Numerous advantages of likelihood based methods have been proposed, such as having greater accuracy and efficiency in finding the correct trees when evolutionary rates vary among branches [14–16]. However, allowances must be made due to the lack of comparability across simulation studies that use different taxon sampling and tree topologies to assess accuracy, as well as different approaches towards measuring accuracy [17]. Furthermore, most of these studies were initially based on molecular data only. More recently, however, some studies have tested the performance of likelihood methods against parsimony using morphological data, which are discussed below.

Evolutionary rate variation is essential to a more biologically realistic phylogenetic inference, and these have become a dominant parameter in likelihood based phylogenetics [18–21]. However, while a Poisson model of substitution with a gamma distribution has been claimed to be an efficient prior for molecular substitution rates [21], there is uncertainty over the best model for morphological characters. While some datasets better fit a gamma distribution of rate variation among characters, others better fit a lognormal distribution [19, 22]. Testing both models for a given dataset seems to be crucial to model based phylogenetic investigations [22], and we thus perform such a test herein (see below).

Despite the possible lower fit of gamma distributions for some morphological datasets, this model still performs better than traditional parsimony in several aspects. For instance, it has been demonstrated (using a dataset of taxon sampling size similar to ours herein) that at variable evolutionary rates, Bayesian inference seem to outperforms maximum parsimony for discrete morphological characters, with and without missing data [23, 24]. Furthermore, Bayesian analyses also have less topological error than traditional parsimony in different scenarios of rate heterogeneity, especially when data for slow-evolving characters are missing [23].

### Dataset

We used the mosasauroid dataset of Bell [1], with the subsequent modifications and additions performed over the last 20 years by numerous authors, and summarized most recently by Palci et al. [12], Jimenez-Huidobro & Caldwell [25] and Jiménez-Huidobro et al. [26]. As our goal was primarily to test different phylogenetic methods, we did not alter the character constructions, neither included nor deleted any character. However, we have changed the outgroup choice, tested different coding schemes, and performedsome changes to ingroup taxon inclusion and scoring (see below).

### Outgroup choice

Outgroup choice is an integral part of all parsimony based methods. All published phylogenies focused on mosasauroid relationships thus far have used a combination of varanid lizard scorings to the composition of a theoretical “outgroup” operational taxonomic unit (OTU). The only major datasets that have used a different approach are the large scale squamate phylogenies that also happen to have a large taxonomic sampling of mosasauroids—e.g. Conrad [27] and Conrad et al. [13]. However, the creation of a composite OTU that does not correspond to a real taxonomic entity creates some technical issues, such as unnecessary polymorphisms in the outgroup. Most importantly, in the case of artificial outgroups, character polarity may be determined *a priori* based on prior beliefs of character evolution (e.g. selection of an all zero, or supposedly plesiomorphic outgroup), whereas polarity should be determined *a posteriori* (an assumption implicit in most parsimony software) and the outgroup taxa to be unconstrained [28, 29].

Another potential caveat of the utilization of *Varanus*, or a varanid composite as an outgroup, is the possibility that mosasauroids may actually be distantly related to varanids, according to some previous discussions on the topic [2], as well as some phylogenetic hypotheses. Other than a sister-clade relationship to varanids [30–34], mosasauroids have been inferred to fall somewhere near the stem of Anguimorpha [31, 35–38], outside of Scleroglossa [39], or as toxicoferans, but outside of Anguimorpha [40]. Although outgroups do not need to be necessarily sister taxa to the ingroup [29], very distantly related OTUs may offer little basis of comparison regarding character evolution in the branch leading to the ingroup.

Instead of *Varanus*, or a combination of varanid features, we added three dolichosaurid lizards to the dataset: *Adriosaurus suessi* Seeley, 1881 [30, 41], *Dolichosaurus longicollis* Owen, 1850 [42, 43]and *Pontosaurus kornhuberi* Caldwell, 2006 [44], designating *Adriosaurus suessi* as the outgroup. These taxa, along with other dolichosaurids, have consistently been found in every analysis of mosasauroid and squamate relationships as closely related to mosasauroids, either as part of a Hennigean comb leading to mosasauroids [9, 13, 27, 40], or as part of their sister clade [30–32, 39, 44, 45]. This should provide a more reasonable comparison of character evolution and polarization relative to the ingroup: mosasaurids. An unconstrained outgroup, including *Adriosaurus* (as the officially designated outgroup) and the two other dolichosaurids also allows testing of the ingroup composition and some of the relationships among the outgroup taxa. Additionally, all taxa recovered as external to mosasaurids will have an influence over the character-state optimization at the node ancestral to the ingroup. Therefore, the criterion for determining the ancestral states to the ingroup during optimization will be the same as the one used for ancestral node optimization as performed for the ingroup ancestral nodes—the Fitch optimization in the case of discrete characters [46] and Farris optimization for ordered characters [47]. We consider this a better approach than constraining a set of taxa as the outgroup.

We have also added to the dataset herein two aigialosaurid species: *Aigialosaurus dalmaticus* Kramberger, 1892 [48, 49] and *Aigialosaurus bucchichi* Kornhuber, 1901 [11, 50]. These taxa have been previously analyzed in mosasauroid datasets, but often combined into a single OTU, or only one of them being used. We consider it relevant to include both as separate OTUs in order to test their position as early evolving mosasauroids, and outgroups to mosasaurids—which is especially relevant when considering the possibility of multiple origins of fully aquatic mosasauroids (see Dutchak & Caldwell [11]). In case they are recovered as early evolving mosasauroids, then they should have a strong influence over the composition and character polarity of early mosasaurids.

### Ingroup taxa

Minor changes to taxon inclusion and scoring were performed, in accordance with recent revisions and incorrect scorings noticed by us. According to recent revisions [51–53], *Mosasaurus maximus* is a junior synonym of *M*. *hoffmannii*, and this synonymization is incorporated in our dataset by exclusion of the *M*. *maximus* OTU. A few incorrect scorings for both *Platecarpus* species, as well as *Latoplatecarpus willistoni* and *Plioplatecarpus*, are corrected herein based on Konishi & Caldwell [54–56]. Additional changes include: Character 29 (maxilla tooth number) was re-scored as ‘1’ for *Tethysaurus nopcsai* based on both literature and personal observation (IP): 19 between actual teeth and tooth positions are found in the holotype and Bardet et al. [57] recognize that there may be room for an extra one, but no more than that. Moreover, 19 teeth are scored in the dentary, and there is no evidence to support a different number in the maxilla. A polymorphism for character 70 (tooth fluting) in *Tethysaurus* was solved by re-coding the character state as ‘0’, following the information available from published material [57].

### Dataset modifications

The inclusion of dolichosaurid and aigialosaurid taxa required the addition of a new character-state to three characters: 37 (state 3), 63 (state 2), 100 (state 2) (see also see also S1 Text), as the conditions observed in these taxa were not accounted for in the existing character-states. Another modification to the dataset was combining six binary characters into multistate characters (see S1 Text and S1 Dataset). These characters were dependent on each other and would be better analyzed under a single transformation series. The dataset with multistate coding merged 6 characters with other pre-existing characters, thus resulting in a final character list of 125 characters. The final number of taxa consisted of 44 taxa. For matters of comparison with numerous previously published results of mosasauroid relationships that used contingently coded characters, we also tested a contingently coded characters dataset containing 131 characters analyzed under unweighted maximum parsimony (Co-UMP—see S2 Dataset).

### Analytical procedures

#### Traditional (unweighted) maximum parsimony (Co-UMP and Mu-UMP)

The morphological dataset was analyzed in the software T.N.T. [58] using the heuristic “Traditional Search” under TBR (100 replicates x 100 iterations). Although the number of taxa in the dataset is relatively low, we further tested the dataset using the “New Technologies Search” algorithms “Ratchet” (1000 iterations), “Sectorial Searches” (1000 rounds), and “Tree Fusing” (1000 rounds), upon the trees initially obtained by the same algorithms and 1,000 Wagner trees obtained by random addition sequence (RAS), in the sequence outlined in Simões et al. [59]. As expected, however, there was no difference in the number or length of the most parsimonious trees obtained by both methods for a dataset of this size.

#### Maximum parsimony under implied weighting (IWMP)

A second parsimony analysis used the algorithm of implied weighting, as described by Goloboff [60, 61], along with TBR (100 replicates x 100 iterations), with the default function of K = 3.0.

#### Maximum likelihood (ML)

The ML analysis was performed in IQ-Tree v. 1.3.10 available on the web server [62, 63]. We selected traditional for morphological data and Mk for the model of substitution model [18] for the analysis of the dataset. Rate variation among sites was modeled using a discrete gamma distribution [64] with eight rate categories (+G8). Effects of the number of rate categories on the phylogenetic reconstructions under the gamma distribution model have been tested for both molecular and morphological data (using ML and Bayesian inference) with similar results showing that four to ten categories are sufficient for the effective approximation of the continuous gamma distribution [21, 22, 64]. Therefore, we selected a number of categories for our analysis from this empirically determined range of values. To account for the absence of invariable characters in the data set, we used an ascertainment bias correction (+ASC). The node support was estimated using the ultrafast bootstrap option with 1000 replicates [65].

#### Bayesian inference

Bayesian analysis of the data set was performed in MrBayes v. 3.2.5 [66]. We used the Mk(V) model that combines traditional Mk model [18] and an ascertainment bias correction to account for the lack of invariable characters in our data set. To explore potential effects of the different distribution models of the rate heterogeneity and state frequencies, we performed four independent analyses with different combinations of settings for these parameters. We tested both gamma (GA) and lognormal (LN) distributions for rate heterogeneity, and uniform (Uni) and exponential (Exp) priors governing the shape of these distributions (α and σ^2^ values). These were combined as follows: (i) Mk(V) + Exp + GA; (ii) Mk(V) + Exp + LN; (iii) Mk(V) + Uni + GA; and (iv) Mk(V) + Uni + LN. Equal rates of variation are systematically found as a less desirable model in comparison to variable rates for Bayesian inference not just of molecular, but also of morphological data [22, 67–71]. Equal rates are also less realistic in a biological sense [18–21]. Therefore, we focused on comparing traditionally used gamma distribution with the lognormal model of the among character rate variation. Each analysis was performed with two independent runs of 1×10^7^ generations each. The relative burn-in fraction was set to 25% and the chains were sampled every 1000 generations. The temperature parameter for the four chains in each independent run was set to 0.01 as determined by preliminary runs to achieve optimal chain mixing values (0.4–0.8). Convergence of independent runs was assessed through the average standard deviation of split frequencies (ASDSF < 0.01) and potential scale reduction factors [PSRF ≈ 1 for all parameters, [72]] calculated at the end of the Bayesian runs. We used Tracer v. 1.6 [73] software to determine whether the runs reached stationary phase and to ensure that the effective sample size (ESS) for each parameter was greater than 200. To estimate the posterior tree with maximum clade credibility (Bayesian MCC), we used TreeAnnotator v. 2.4.3 [74].

#### Topology tests

In order to test whether the tree topologies obtained under the different methods above were significantly different from each other, we performed a parsimony based Wilcoxon signed-ranks test [75] in PAUP* 4.0a146 [76] and a likelihood-based Shimodaira–Hasegawa test [SH test [77]] as implemented in IQ-tree v. 1.3.10 [62, 63]. In each test, the following topologies were compared: 84 most parsimonious trees from the Mu-UMP analysis, 30 MPTs from the Co-UMP analysis, the best-fit IWMP tree, the ML tree, and the final trees from each of the four independent Bayesian analyses represented as majority-rule consensus trees. For the SH test, 1000 bootstrap replicates were resampled using the re-estimated log likelihoods (RELL) method.

#### Model tests

Recent studies have shown that substitution rates in morphological data may better fit a lognormal distribution instead of a gamma distribution [19, 22], the latter being the most commonly implemented model for likelihood based methods. When there is a difference in the distribution model fit to the data, a lognormal distribution is usually the better fit model. However, fit to the data is dependent on the dataset, and model tests are strongly recommended [22]. Unfortunately, all maximum likelihood softwares known to us that implement the Mk model for morphological data do not implement the lognormal distribution model. Therefore, model testing and subsequent runs with distinct distribution parameters were performed for the Bayesian inference analyses only. For Bayesian inference, model fit was tested using Bayes factors [*B*_10_] and calculated using the marginal model likelihoods [$\hat{f}(X|M)$] [71] by applying the stepping-stone sampling (SS) method [78]. Model likelihoods using SS as implemented in Mr. Bayes v. 3.2 [66] provides greater accuracy and allows comparisons across different priors when compared to model likelihoods using harmonic means [22, 78, 79]. The interpretation of the results of the model fit to the data follows previous authors [22, 67, 69–71, 80] in using the values provided by Kass & Raftery [81] as a common basis of comparison: when 2*log*_*e*_(*B*_10_) > 2 (positive evidence against model *M*_0_); when 2*log*_*e*_(*B*_10_) > 6 (strong evidence against model *M*_0_); when 2*log*_*e*_(*B*_10_) > 10 (very strong evidence against model *M*_0_).

*Character mapping*: Characters were mapped in Mesquite v.3.04 [82] utilizing the “Trace Character History” tool. Character history was established using parsimony reconstruction of characters states for parsimony inferred trees obtained from T.N.T. Likelihood reconstruction of character states was used for the tree topologies and associated branch lengths from the Bayesian consensus tree and the maximum likelihood tree imported from the model based software packages, using the Mk1 probability model.

## Results

**Fig 1:**
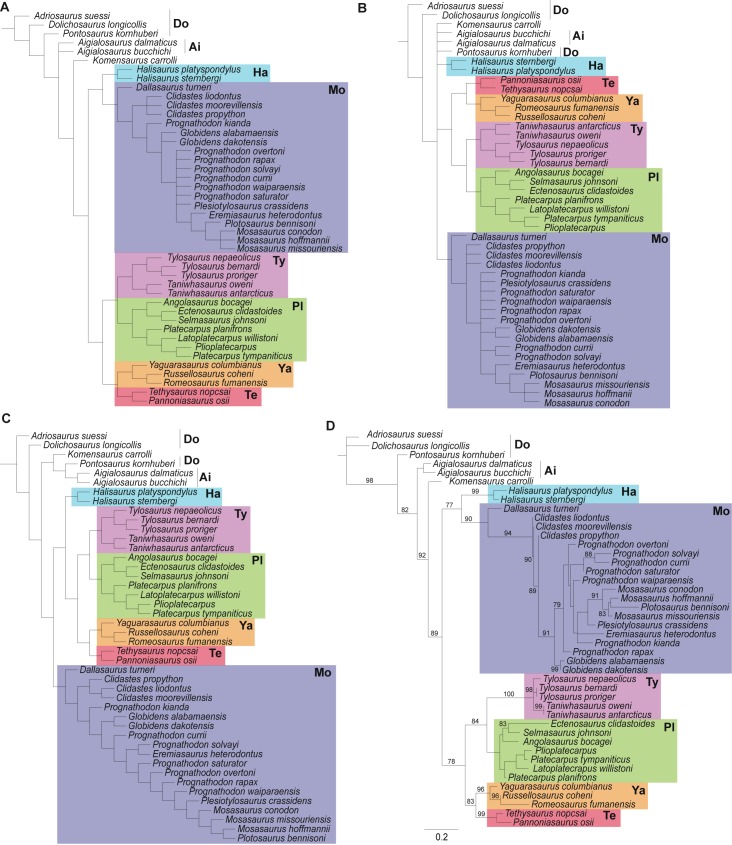
Phylogenetic analysis of mosasauroid relationships using different methods. (A) Co-UMP: strict consensus of 30 most parsimonious trees (450 steps each) (CI = 0.350; RI = 0.692). (B) Mu-UMP: strict consensus of 84 most parsimonious trees (445 steps each) (CI = 0.329; RI = 0.660; length). (C) IWMP (fit = 45.45942; CI = 0.360; RI = 0.706; length = 449 steps). (D) ML tree. For the ML tree, branches are proportional to their length, values above branches indicate bootstrap support and scale bar represents branch lengths. Abbreviations: Ai, Aigialosauridae; Do, Dolichosauridae; Ha, Halisaurinae; Mo, Mosasaurinae; Pl, Plioplatecarpinae; Te, Tethysaurinae; Ty, Tylosaurinae; Ya, Yaguarasaurinae.

Despite the change in the outgroup, and minor updates in the ingroup taxa and scorings, the analysis with Co-UMP (the coding and search method used by most mosasauroid phylogenies) provided results (Fig 1A) that are generally similar to the most recent analyses of mosasauroid relationships [12, 25, 26]. Namely, aigialosaurs lie at the base of the lineage leading to mosasaurids; Mosasaurinae is monophyletic (and inclusive of Halisaurinae and *Dallasaurus*); and Russellosaurina is also monophyletic (inclusive of yaguarasaurines, tethysaurines, plioplatecarpines and tylosaurines). Our Co-UMP results are also similar to those of Palci et al. [12] and Jimenez-Huidobro & Caldwell [25], regarding the position of *Komensaurus* and halisaurines.

The Mu-UMP analysis offers the least resolved topology when compared to all other resultant topologies (Co-UMP, IWMP, ML, Bayesian)—Fig 1B. The monophyly of Mosasauroidea (Aigialosauridae + Mosasauridae) and Mosasauridae [sensu 1] could not be verified due to a polytomy that includes *Pontosaurus*, *Komensaurus*, aigialosaurids, halisaurines, russellosaurines and mosasaurines. *Dallasaurus* is recovered at the base of a monophyletic Mosasaurinae, as in previous phylogenies [e.g., 8, 12, 83, 84], and Russellosaurina [84] was also found as a monophyletic group.

A better resolved and alternative hypothesis is offered instead by the IWMP analysis (Fig 1C). A major difference between the IWMP tree and the Mu-UMP strict consensus is the placement of halisaurines as the sister group of Russellosaurina rather than of Mosasaurinae (as in the Co-UMP strict consensus). Additionally, aigialosaurs are found in a clade with *Pontosaurus* and *Komensaurus* as early evolving mosasauroids. Moreover, because the best-fit tree represented by the IWMP is not a consensus tree, the relationships amongst the less inclusive mosasaurines clades (i.e., *Clidastes*, *Globidens*, *Prognathodon* and *Mosasaurus* groups) are all fully resolved, thus differing from both other maximum parsimony trees (Co- and Mu-UMP). As in previous phylogenies, and all our topologies in which enough resolution was obtained, *Prognathodon* is confirmed to represent a paraphyletic genus [cf. 12, 83].

The maximum likelihood tree recovers some of the relationships observed in both the Co-UMP strict consensus and the IWMP trees (Fig 1D). For instance, *Pontosaurus* is the sister taxon of Mosasauroidea, aigialosaurids are monophyletic and lie at the base of the mosasauroids, and *Komensaurus* is the sister taxon to Mosasauridae. Mosasaurines and Russellosaurina are recovered as monophyletic, and as in the Co-UMP, *Dallasasaurus* is found along with halisaurines at the base of the lineage leading to Mosasaurinae.

In the Bayesian inference analyses four different combinations of models were used, and their fit to the data tested using Bayes Factors (see methods and Table 1). The models fit to the data indicate the lognormal distribution was positively preferred over the gamma distribution under both shape priors (uniform and exponential), although not strongly preferred: 6 > 2*log*_*e*_(*B*_10_) > 2. When comparing both priors, there is no positive preference for any model: 2*log*_*e*_(*B*_10_) < 2.

**Table 1:** Model likelihoods and bayes factors for the analyses performed.

*M*_1_/*M*_0_	Mean marginal model log likelihood		Bayes Factor	
	${log}_{e}\hat{f}(X|M_{1})$	${log}_{e}\hat{f}(X|M_{0})$	log_*e*_*B*_10_	2log_*e*_*B*_10_
Exp-LN/Exp-GA	-1671.54	-1673.62	2.08	4.16
Exp-LN/Uni-LN	-1671.54	-1671.66	0.12	0.24
Exp-GA/Uni-GA	-1673.62	-1673.83	0.21	0.42
Uni-LN/Uni-GA	-1671.66	-1673.83	2.17	4.34

Exp, exponential hyperprior on the shape of the gamma or lognormal distributions; GA, gamma distribution of among character rate variation; LN, lognormal distribution of among character rate variation; Uni, uniform (flat) hyperprior on the shape of the gamma or lognormal distributions

**Fig 2:**
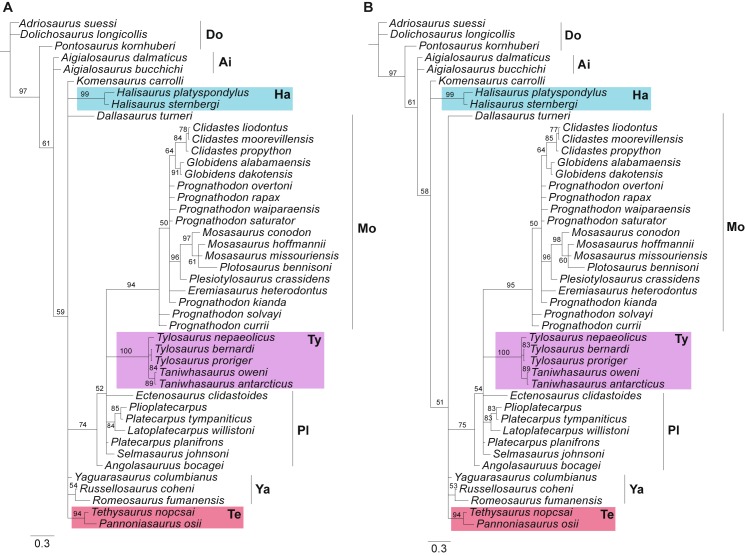
Bayesian majority rule consensus tree drawn from 15,002 posterior trees (lognormal prior on rate variation across characters). (A) Using an exponential hyperprior on the shape of the lognormal distribution. (B) Using a uniform hyperprior on the shape of the lognormal distribution. Branches are proportional to their length. Values above branches indicate clade probabilities and scale bar represents branch lengths. Abbreviations: Ai, Aigialosauridae; Do, Dolichosauridae; Ha, Halisaurinae; Mo, Mosasaurinae; Pl, Plioplatecarpinae; Te, Tethysaurinae; Ty, Tylosaurinae; Ya, Yaguarasaurinae.

The trees obtained from Bayesian inference with the lognormal distributions are depicted in Fig 2, using the exponential (Fig 2A) and the uniform (Fig 2B) priors. All trees (see also S1 Fig) are characterized by a greater lack of resolution in multiple sectors of the tree when compared to the final trees from Co-UMP, IWMP, and ML analyses, although more similar in this aspect to the Mu-UMP strict consensus tree. Importantly, they are unique in several aspects, and deviate the most from the other trees (Table 2).

**Table 2:** Summary of the Wilcoxon signed-ranks and Shimodaira-Hasegawa tests (SH) test results for topologies generated under the different search methods.

Method	Templeton		SH	
	Length	P	-LogL	P
**Mu-UMP**	445	0.9857–1.0000	2336.973–2353.912	0.5070–0.9890
**Co-UMP**	446–447	0.6374–1.0000	2343.877–2352.36	0.5730–0.8800
**IWMP**	449	0.4142	2367.288	0.2370
**ML**	447	0.7327	2334.732	1.0000
**Bayesian inference**				
**Exp+GA**	521	<0.0001*	2441.756	0.0000*
**Exp+LN**	511	<0.0001*	2425.579	0.0000*
**Uni+GA**	525	<0.0001*	2448.811	0.0000*
**Uni+LN**	505	<0.0001*	2416.245	0.0000*

Results are summarized for all trees obtained with each method and presented as a range of values (for the Bayesian analyses, results are shown for each different combination of parameters). Abbreviations: Co-UMP_,_ unweighted parsimony analysis with contingent character coding; IWMP, maximum parsimony under implied weighting; ML–maximum likelihood analysis; Mu-UMP_,_ traditional unweighted parsimony analysis with multistate character coding.

* Statistically significant values indicating tree topologies that are not equally well-explained by the data and significantly differ from the other analysed topologies.

The majority rule consensus trees from the Bayesian inference analyses recovered relationships amongst the earliest branching lineages (dolichosaurs and aigialosaurs) that are very similar to the ones in the ML and Co-UMP trees. However, for all the other major clades and terminal taxa there are quite important re-arrangements. Within Mosasauroidea, halisaurines are recovered in a polytomy with *Komensaurus*, *Dallasaurus*, “yaguarasaurines”, tethysaurines and the branch leading to all the other mosasaurids. Yaguarasaurinae is not recovered as monophyletic, and neither are Plioplatecarpinae, Russellosaurina or Mosasaurinae—if *Dallasaurus* is considered to be part of Mosasaurinae, as in most previous studies including that taxon [7, 8, 12, 25, 83, 85]. Most mosasaurines, however, do form a clade if *Dallasaurus* is not considered as a member of that group. The most notable difference between all Bayesian analyses and the remaining ones is the position of taxa usually classified within Russellosaurina. Tylosaurines (recovered as monophyletic) and most “plioplatecarpines” are found in a polytomy with Mosasaurinae (exclusive of *Dallsasaurus*). In three of the four Bayesian trees (Exp-GA, Uni-LN, Exp-LN), *Angolasaurus* is recovered as the sister taxon to the clade inclusive of other “plioplatecarpines”, tylosaurines and mosasaurines (excluding *Dallasaurus*), whereas in the Uni-GA Bayesian analysis it is in the polytomy with the aforementioned groups. Within Mosasaurinae (exclusive of *Dallsasaurus*), all species of *Mosasaurus* form a clade with *Plotosaurus bennisoni* and *Plesiotylosaurus crassidens*. Additionally, all species of *Clidastes* form a clade with the two *Globidens* OTUs. In all of the Bayesian trees, *Prognathodon* is a paraphyletic genus, with *P*. *curri* and *P*. *solvayi* recovered as the earliest derived mosasaurines in the two Bayesian trees with lognormal distribution of substitution rates (the ones with better Bayes Factor indices herein).

**Fig 3:**
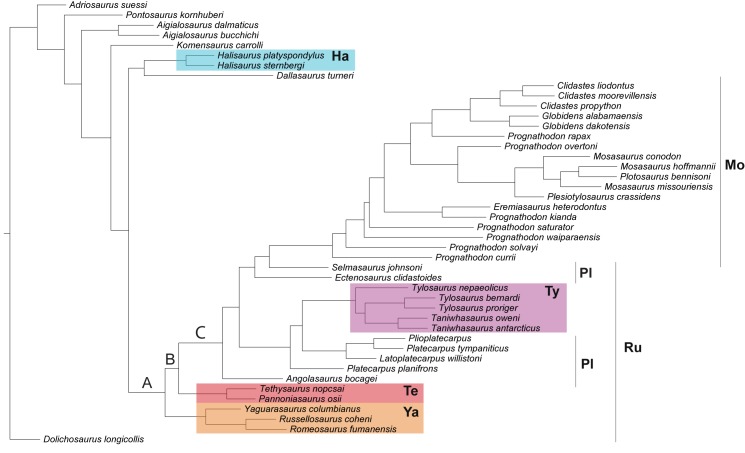
Maximum posterior clade credibility tree. Tree obtained with a lognormal prior on rate variation across characters and a uniform hyperprior (see text for details). Abbreviations: Ha, Halisaurinae; Mo, Mosasaurinae; Pl, Plioplatecarpinae; Ru, Russellosaurina; Te, Tethysaurinae; Ty, Tylosaurinae; Ya, Yaguarasaurinae.

The trees with maximum clade posterior probability values from the Bayesian inference analyses, just as the maximum likelihood tree, indicate the tree with the highest values for the optimality criterion for this method (posterior probabilities). The maximum credibility tree (Fig 3) was obtained as the product of the clades posterior probabilities (see also Methods). In both the maximum credibility tree and the majority rule consensus tree, russellosaurines are paraphyletic, forming a comb leading to mosasaurines (without *Dallasaurus*). Additionally, plioplatecarpines are polyphyletic, whereas tylosaurines are once more found as monophyletic. Clades A, B and C in the maximum credibility tree indicate new broad level topological relationships amongst mosasauroid clades obtained only by Bayesian inference results, with clade C being the most consistent as it is recovered across most of the posterior trees, thus being recovered in the consensus tree too (Fig 2).

## Discussion

Despite some modifications performed in this study to the data matrix being utilized, this dataset still includes the characters and most ingroup taxa used by different authors since Bell & Polcyn [8]. As a consequence, there is an overall topology similarity between the strict consensus of the Co-UMP tree (Fig 1A) and the results of most studies published in the past decade, regarding the position of aigialosaurids, the monophyly and composition of Russellosaurina, and the monophyly of Mosasaurinae (although the internal relationships of the latter clade can be quite variable). Therefore, most of the major differences in the trees obtained by the other remaining methods relative to recently published studies (and our Co-UMP tree) should be explained mostly by differences in the methods of tree inference being implemented (implied weighting, maximum likelihood and Bayesian inference), or coding method (contingent vs multistate).

The lack of resolution in our Mu-UMP (Fig 1B) when compared to the same tree using contingent coding (Co-UMP, Fig 1A) may be a consequence of the loss of resolution provided by unordered multistate character coding, at least for parsimony-based analyses [86]. This loss of resolution has the consequence of binary characters in the dataset playing a relatively greater influence to the resulting trees [87]. Despite this effect, other datasets in which numerous ordered multistate characters have been re-analyzed as unordered have not shown an overall decrease in resolution in the strict consensus—e.g. Simões et al. [88]. Additionally, only a small portion of the original characters were converted into multistate characters. Thus, we conjecture that finding reduced resolution in the strict consensus of the multistate dataset should also indicate a lack of agreement among characters in the present dataset, and not just a loss of resolution due to multistate coding.

The majority rule consensus of the posterior trees output from the Bayesian analyses (Fig 2) also had less resolution than the Co-UMP strict consensus and the ML trees. The Bayesian inference trees were also less resolved than preliminary runs using equal rates of variation (not shown). However, as noted in the Introduction, equal rates of variation are systematically found as a less desirable model in comparison to variable rates for Bayesian inference, both due to lack of accuracy and because of the implicit biological assumptions. Importantly, adding more parameters and rates of variation to likelihood based analyses increases the topology variance being recovered, and this may occasionally result in decreased resolution [71]. Furthermore, analyses under the Mk model have been shown to have decreased resolution compared to parsimony methods when analysed using Bayesian inference [24, 89]. Therefore, it is suggested that adding rates of variation to the mosasauroid dataset tested here might be responsible for the decreased resolution when compared to the Bayesian inference under equal rates. Decreased resolution by the Mk model using Bayesian inference compared to parsimony methods might also explain the decreased resolution of the Bayesian trees relative to the Co-UMP tree.

It is interesting to note that the polytomy recovered in the early divergence of mosasauroids in the Mu-UMP and Bayesian analyses also provides a good representation of the lack of agreement and conflicting results regarding the position of *Komensaurus* and halisaurines relative to other mosasauroids in previous analyses: sometimes recovered as early mosasauroids [1, 10, 11, 27, 42], or within russellosaurines [7, 8, 49, 83, 85], or with *Komensaurus* as an early mosasauroid and halisaurines as early mosasaurines [12, 25]. These numerous previous conflicting results for the phylogenetic position of these taxa (as well as our results) indicate their phylogenetic position is far from fully understood and thus remains poorly resolved.

The problematic taxon *Dallasaurus* is most often recovered at the early divergence of, or as the sister taxon to, mosasaurines [7, 8, 12, 25, 83, 85]. Dutchak & Caldwell [11] had previously indicated an alternative view, with *Dallasaurus* as an early mosasauroidalong with halisaurines and *Haasiasaurus*. Conflicting results with the commonly recovered position for *Dallasaurus* (at the base of or sister taxon to mosasaurines), were obtained by three distinct tree inference methods herein: traditional parsimony with multistate characters (Mu-UMP), implied weighting (IWMP) and Baysian inference. Although we also recovered *Dallasaurus* as an early mosasaurine using ML, our results urge caution for the placement of *Dallasaurus*. Taking into consideration the also problematic placement of halisaurines and *Komensaurus*, we consider these three taxa to be critical towards a better understanding of the early radiation of mosasauroid reptiles, and the acquisition of pelvic and autopodial anatomies fully adapted to aquatic locomotion (see more below).

### The acquisition and potential loss of features related to aquatic locomotion

One of the most discussed topics concerning the evolution of mosasauroid reptiles regards the origin of the hydropelvic condition, which is associated with the achievement of a fully aquatic lifestyle. As discussed by Caldwell & Palci [7], the retention of the sacrum in plesiopelvic mosasauroids might have allowed the capacity to move ashore, while the hydropelvic condition (with a total loss of any bony articulation between the vertebral column and the pelvic girdle—see character 89 and 117 in S1 Text) represents a transition to a fully aquatic lifestyle. In addition to these modifications of the sacral region, fore and hind limbs also underwent a set of modifications that facilitate locomotion in aquatic environments. The autopodials, for instance, are modified in most mosasauroids into paddle-shaped structures. The set of transformations leading to that configuration include changes from a plesiopedal condition, as typified by the configuration seen in aigialosaurs (early mosasauroids)—phalangeal formula (i.e., 2-3-4-5-3 or 2-3-4-5-4)—to a hydropedal condition, characterized by hyperphalangy (especially in digits II-to-IV), and which may occur at different degrees of development [e.g., 7, 12, 83]—character 123.

Previous phylogenetic hypotheses suggest both hydropelvic and hydropedal configurations have multiple origins [7, 8, 11, 12]. In our results, however, we detect a different evolutionary scenario by mapping the characters that account for these morphologies in the current dataset. Due to lack of resolution in the Mu-UMP and Bayesian inferences, character mapping using optimization of ancestral character-states was possible for the Co-UMP, IWMP and ML trees only.

**Fig 4:**
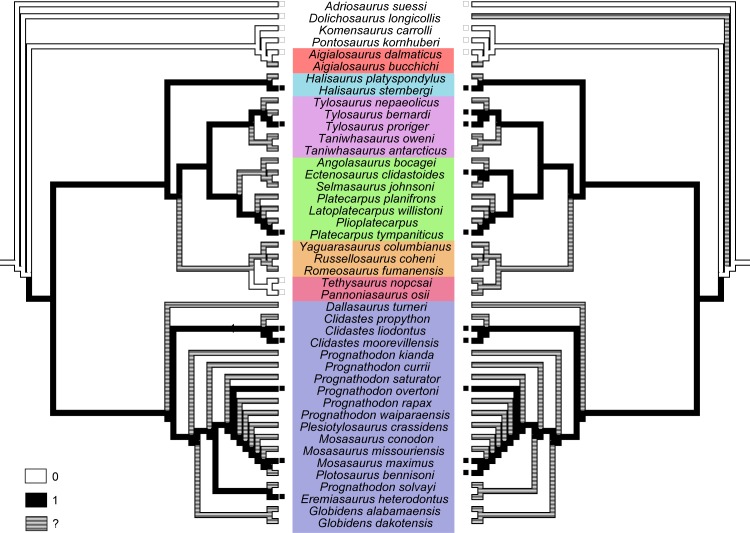
Maximum parsimony ancestral trait optimization on the IWMP best fit tree. Characters maps indicate evolutionary change of character 117 on the pelvic condition (left) and 123 on the pedal condition (right). White branches indicate state “0”, black branches indicate state “1”, and branches in shades of gray indicate missing data or ancestral state ambiguity.

In the IWMP tree (with halisaurines as early russellosaurines), both hydropelvic and hydropedal conditions evolve only once in the early evolution of Mosasauridae, with the pelvic condition reversed in tethysaurines (Fig 4). The pedal condition in tethysaurines is currently unknown, thus if reversals in the pelvic condition are coupled with pedal changes, this cannot be assessed given the present data. In the Co-UMP and ML trees, hydropelvic and hydropedal conditions are ancestral to all mosasaurines (as they are in the IWMP tree). However, the placement of the group formed by Tethysaurinae+Yaguarasaurinae as the sister clade to other russellosaurines (and halisaurines as early mosasaurines), makes the optimization of the branch that is ancestral to all russellosaurines ambiguous in both the Co-UMP and ML trees (Table 3 and S2 Fig). The ancestral state reconstruction for the ML tree gives a higher likelihood that the ancestral condition for russellosaurines is hydropelvic (Table 3), which would favour the hypothesis of reversal among tethysaurines, as also implied by the IWMP analysis.

**Table 3:** Ancestral state likelihood reconstruction (Mk model) for pelvic and pedal characters obtained from the ML tree.

Clade	Ch. 89		Ch. 117		Ch. 123	
	State 0	State 1	State 0	State 1	State 0	State 1
**Mosasaurinae**	0.06	0.94	0.047	0.953	0.004	0.996
**Russellosaurina**	0.399	0.601	0.313	0.687	?	?
**Mosasauridae**	0.543	0.457	0.48	0.52	0.072	0.928

Proportional ancestral state likelihoods (using Mk model) for the three main mosasauroid clades. Characters 89 and 117 relate to pelvic and 123 to pedal morphologies. State “0” is associated with plesiopelvic/pedal conditions and state “1” with hydropelvic/pedal conditions.

? Indicate ambiguous likelihoods that could not be calculated.

**Fig 5:**
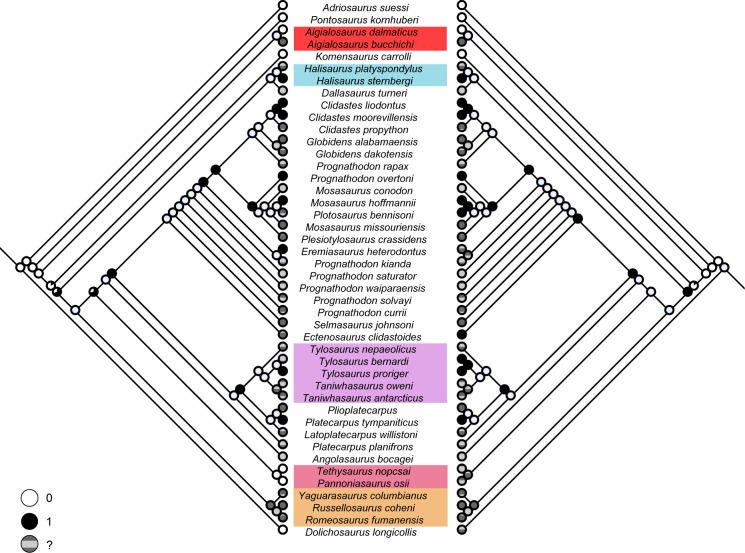
Likelihood ancestral trait reconstruction on the Bayesian MCC tree. Characters maps indicate evolutionary change of character 117 on the pelvic condition (left) and 123 on the pedal condition (right). White nodes indicate 100% likelihood for state “0”, black nodes indicate 100% likelihood for state “1”, and nodes in shades of gray indicate missing data or ancestral state ambiguity. Nodes with both black and white colors indicate the proportional likelihoods of state “0”(white) and state “1” (black) in a pie chart format.

Ancestral state reconstruction using on the Bayesian MCC tree also supports a higher likelihood for the hydropelvic condition as the ancestral one for Mosasauridae and for Clade B (Fig 5). However, the likelihood do not reach close to 0.95 (see Table 4), and so this result should be seen with caution. Regarding the evolution of the pedal morphology, there is a high likelihood of the hydropedal condition being ancestral for the whole Mosasauridae. Although the pedal condition in tethysaurines is currently unknown, if their pedal morphology evolved along with the pectoral morphology then it should be expected that tethysaurines possess a plesiopedal condition. This would favor the hypothesis of reversal to a plesiopelvic/pedal condition in this clade under the Bayesian MCC tree.

**Table 4:** Ancestral state likelihood reconstruction (Mk model) for pelvic and pedal characters obtained from the Bayesian maximum clade credibility tree.

Clade	Ch. 89		Ch. 117		Ch. 123	
	State 0	State 1	State 0	State 1	State 0	State 1
**Mosasauridae**	0.432	0.568	0.314	0.686	0.033	0.967
Clade B*	0.419	0.580	0.291	0.709	?	?

Proportional ancestral state likelihoods (using Mk model) for the three main mosasauroid clades. Characters 89 and 117 relate to pelvic and 123 to pedal morphologies. State “0” is associated with plesiopelvic/pedal conditions and state “1”with hydropelvic/pedal conditions.

* Clade B is chosen herein because the node from where this clade branches from is also the most recent ancestral node to tethysaurines, thus revealing the likelihood in the most recent common ancestor for that clade.

? Indicate ambiguous likelihoods that could not be calculated.

Therefore, two hypotheses remain concerning the ancestral condition for russellosaurines (found as a clade in most trees): hydropelvic and hydropedal conditions are ancestral to russellosaurines (and thus to all Mosasauridae too because of the condition in Mosasaurinae) with a reversal in tethysaurines; or plesiopelvic/pedal conditions are ancestral to all russellosaurines, with tethysaurines representing the plesiomorphic condition, and thus that hydropelvic/pedal condition evolved independently in other russellosaurines relative to mosasaurines. Under the ML tree and the Bayesian MCC tree, there is also ambiguity regarding the early evolution of pelvic and pedal conditions in mosasaurs, but a greater likelihood is given to an early evolution of hydropelvic/pedal conditions for all mosasaurids, meaning that tethysaurines may represent a case of reversal under this hypothesis (similarly to the ML tree results).

## Conclusions

Our results suggest that even among methods that take branch lengths, evolutionary rates, and homoplasy rates into consideration, there is no conclusive solution for the placement of halisaurines and *Dallasaurus*. Additionally, under Bayesian inference, Russellosaurina is paraphyletic. Yet, all the results agree on the monophyly of Mosasaurinae (exclusive of *Dallasaurus*), Tylosaurinae, and the paraphyly of the genus *Prognathodon*, Additionally, we found no phylogenetic hypothesis supporting the recently proposed convergent evolution of the hydropelvic/pedal conditions in mosasauroids [7, 8, 11, 12]. Instead, the only unambiguous result obtained concerning the semi-to-fully aquatic transition in mosasauroids (from the implied weighting analysis) indicates a single origin of hydropelvic/pedal mosasauroids, with a reversal within tethysaurines for the pelvic condition. Additionally, despite some ambiguity under the ML and Bayesian MCC trees, a greater likelihood is given to a reversal among tethysaurines to a plesiopelvic condition.

One of the main causes of divergence between the resolved topologies obtained herein (using Co-UMP, IWMP, ML and Bayesian MCC tree), are the phylogenetic position of halisaurines, *Dallasaurus*, and the early evolution of russellosaurines. Combined with the discussions above, we consider that in order to better understand the semi-to-fully aquatic transition in mosasauroids, and evaluate potential reasons for the seeming reversal condition in plesiopelvic forms, two areas of investigation need to be further developed: i) a deep re-assessment of the character construction used to infer mosasauroid relationships. Our observations strongly suggest that a significant portion of the characters currently used in all phylogenetic investigations of mosasauroid relationships—all derived as modification of Bell [1]—might fall in a number of problematic character categories recently identified by Simões et al. [88]; and ii) a revision of relevant taxa in the early evolution of mosasauroids, in particular regard to halisaurines, yaguarasaurines and *Dallasaurus*.

It is also recommended that future investigations concerning mosasauroid evolution should take into account phylogenetic procedures that can account for great disparity in branch lengths and evolutionary rates. In the case of Bayesian inference, this method yields quite distinct relationships among mosasauroids from most previous studies. Considering the seemingly greater performance of Bayesian methods over other phylogenetic procedures regarding accuracy in datasets of similar size to the one tested herein [23, 24, 89], topological relationship that are resultant from Bayesian inference must be taken into account (despite some potential loss of resolution).

## Supporting information

S1 Dataset Nexus file with the dataset analysed herein using multistate coding. (NEX)

S2 Dataset Nexus file with the dataset analysed herein using contingent coding. (NEX)

S1 Fig Bayesian trees. Majority rule consensus trees obtained from the four different prior and hyperprior choice combinations performed herein.
(PDF)

S2 Fig Character maps on trees derived from all the analyses performed herein. (PDF)

S1 Text New characters list for the dataset used herein. (DOCX)

## References

[1] Bell GL Jr. A phylogenetic revision of North American and Adriatic Mosasauroidea In: Callaway JM, Nicholls EL, editors. Ancient Marine Reptiles. San Diego: San Diego Academic Press; 1997 pp. 293–332.

[2] Caldwell MW. A challenge to categories: “What, if anything, is a mosasaur?”. Bull Soc Geol Fr. 2012; 183: 7–34.

[3] Russell DA. Systematics and morphology of American mosasaurs (Reptilia, Sauria). Bull Peabody Mus Nat Hist. 1967; 23: 1–237.

[4] Kocsis L, Ősi A, Vennemann T, Trueman CN, Palmer MR. Geochemical study of vertebrate fossils from the Upper Cretaceous (Santonian) Csehbánya Formation (Hungary): Evidence for a freshwater habitat of mosasaurs and pycnodont fish. Palaeogeogr, Palaeoclimatol, Palaeoecol. 2009; 280: 532–42.

[5] Garcia G, Bardet N, Houssaye A, Pereda-Suberbiola X, Valentin X. Mosasauroid (Squamata) discovery in the Late Cretaceous (Early Campanian) continental deposits of Villeveyrac–L’Olivet, southern France. Comptes Rendus Palevol. 2015; 14: 495–505.

[6] Motani R. The evolution of marine reptiles. Evu Edu Outreach. 2009; 2: 224–35.

[7] Caldwell MW, Palci A. A new basal mosasauroid from the Cenomanian (U. Cretaceous) of Slovenia with a review of mosasauroid phylogeny and evolution. J Vert Paleontol. 2007; 27: 863–80.

[8] Bell G, Polcyn M. *Dallasaurus turneri*, a new primitive mosasauroid from the Middle Turonian of Texas and comments on the phylogeny of Mosasauridae (Squamata). Neth J Geosci. 2005; 84: 177.

[9] Caldwell MW. On the aquatic squamate *Dolichosaurus longicollis* Owen, 1850 (Cenomanian, Upper Cretaceous), and the evolution of elongate necks in Squamates. J Vert Paleontol. 2001; 20: 720–35.

[10] Caldwell MW. Description and phylogenetic relationships of a new species of *Coniasaurus* Owen, 1850 (Squamata). J Vert Paleontol. 1999; 19: 438–55.

[11] Dutchak AR, Caldwell MW. A redescription of *Aigialosaurus* (= *Opetiosaurus*) *bucchichi* (Kornhuber, 1901)(Squamata: Aigialosauridae) with comments on mosasauroid systematics. J Vert Paleontol. 2009; 29: 437–52.

[12] Palci A, Caldwell MW, Papazzoni CA. A new genus and subfamily of mosasaurs from the Upper Cretaceous of northern Italy. J Vert Paleontol. 2013; 33: 599–612.

[13] Conrad JL, Ast JC, Montanari S, Norell MA. A combined evidence phylogenetic analysis of Anguimorpha (Reptilia: Squamata). Cladistics. 2011; 27: 230–77. 10.1111/j.1096-0031.2010.00330.x 34875778

[14] Kuhner MK, Felsenstein J. A simulation comparison of phylogeny algorithms under equal and unequal evolutionary rates. Mol Biol Evol. 1994; 11: 459–68. 8015439 10.1093/oxfordjournals.molbev.a040126

[15] Saitou N, Imanishi T. Relative efficiencies of the Fitch-Margoliash, maximum-parsimony, maximum-likelihood, minimum-evolution, and neighbor-joining methods of phylogenetic tree construction in obtaining the correct tree. Mol Biol Evol. 1989; 6: 514–25.

[16] Huelsenbeck JP, Rannala B. Phylogenetic methods come of age: testing hypotheses in an evolutionary context. Science. 1997; 276: 227–32. 9092465 10.1126/science.276.5310.227

[17] Kim J. Large-scale phylogenies and measuring the performance of phylogenetic estimators. Syst Biol. 1998; 47: 43–60. 12064240 10.1080/106351598261021

[18] Lewis PO. A Likelihood Approach to Estimating Phylogeny from Discrete Morphological Character Data. Syst Biol. 2001; 50: 913–25. 12116640 10.1080/106351501753462876

[19] Wagner PJ. Modelling rate distributions using character compatibility: implications for morphological evolution among fossil invertebrates. Biol Lett. 2012; 8: 143–6. doi: 10.1098/rsbl.2011.0523 21795266 10.1098/rsbl.2011.0523 PMC3259953

[20] Yang Z, Rannala B. Molecular phylogenetics: principles and practice. Nature Reviews Genetics. 2012; 13: 303–14. doi: 10.1038/nrg3186 22456349 10.1038/nrg3186

[21] Ronquist F, van der Mark P, Huelsenbeck JP. Bayesian phylogenetic analysis using MrBayes In: Lemey P, Salemi M, Vandamme A-M, editors. The phylogenetic handbook. Cambridge: Cambridge University Press; 2009.

[22] Harrison LB, Larsson HC. Among-character rate variation distributions in phylogenetic analysis of discrete morphological characters. Syst Biol. 2015; 64: 307–24. doi: 10.1093/sysbio/syu098 25527198 10.1093/sysbio/syu098

[23] Wright AM, Hillis DM. Bayesian Analysis Using a Simple Likelihood Model Outperforms Parsimony for Estimation of Phylogeny from Discrete Morphological Data. PLoS ONE. 2014; 9: e109210 doi: 10.1371/journal.pone.0109210 25279853 10.1371/journal.pone.0109210 PMC4184849

[24] O'Reilly JE, Puttick MN, Parry L, Tanner AR, Tarver JE, Fleming J, et al Bayesian methods outperform parsimony but at the expense of precision in the estimation of phylogeny from discrete morphological data. Biol Lett. 2016; 12. 10.1098/rsbl.2016.0081 PMC4881353 27095266

[25] Jimenez-Huidobro P, Caldwell MW. Reassessment and reassignment of the early Maastrichtian mosasaur *Hainosaurus bernardi* Dollo, 1885, to *Tylosaurus* Marsh, 1872. J Vert Paleontol. 2016; 36: e1096275.

[26] Jiménez-Huidobro P, Simões TR, Caldwell MW. Re-characterization of *Tylosaurus nepaeolicus* (Cope, 1874) and *Tylosaurus kansasensis* Everhart, 2005: Ontogeny or sympatry? Cretaceous Res. 2016; 65: 68–81.

[27] Conrad JL. Phylogeny and systematics of Squamata (Reptilia) based on morphology. Bull Am Mus Nat Hist. 2008; 310: 1–182.

[28] Kitching IJ, Forey PL, Humphries CJ, William DD. Cladistics: the theory and practise of parsimony analysis 2° edition ed. Oxford, New York & Tokyo: Oxford University Press; 1998.

[29] Nixon KC, Carpenter JM. On outgroups. Cladistics. 1993; 9: 413–26. 10.1111/j.1096-0031.1993.tb00234.x 34929985

[30] Lee MS, Caldwell MW. *Adriosaurus* and the affinities of mosasaurs, dolichosaurs and snakes. J Paleontol. 2000; 74: 915–37.

[31] Lee MSY. Squamate phylogeny, taxon sampling, and data congruence Org Divers Evol. 2005; 5: 25–45.

[32] Lee MS. Molecular evidence and marine snake origins. Biol Lett. 2005; 1: 227–30. doi: 10.1098/rsbl.2004.0282 17148173 10.1098/rsbl.2004.0282 PMC1626205

[33] Lee MSY. The phylogeny of varanoid lizards and the affinities of snakes. Philos Trans R Soc Lond, Ser B: Biol Sci. 1997; 352: 53–91.

[34] Lee MSY. Convergent evolution and character correlation in burrowing reptiles: towards a resolution of squamate relationships. Biol J Linn Soc. 1998; 65: 369–453.

[35] Caldwell MW. Squamate phylogeny and the relationships of snakes and mosasauroids. Zool J Linn Soc. 1999; 125: 115–47.

[36] Caldwell MW, Carroll RL, Kaiser H. The pectoral girdle and forelimb of *Carsosaurus marchesetti* (Aigialosauridae), with a preliminary phylogenetic analysis of mosasauroids and varanoids. J Vert Paleontol. 1995; 15: 516–31.

[37] Evans SE, Wang Y. The Early Cretaceous lizard *Dalinghosaurus* from China. Acta Palaeontol Pol. 2005; 50: 725.

[38] Evans SE, Wang Y, Li C. The Early Cretaceous lizard genus *Yabeinosaurus* from China: resolving an enigma. J Syst Palaeont. 2005; 3: 319–35.

[39] Gauthier JA, Kearney M, Maisano JA, Rieppel O, Behlke ADB. Assembling the squamate tree of life: perspectives from the phenotype and the fossil record. Bull Peabody Mus Nat Hist. 2012; 53: 3–308.

[40] Reeder TW, Townsend TM, Mulcahy DG, Noonan BP, Wood PL Jr., Sites JW Jr., et al Integrated Analyses Resolve Conflicts over Squamate Reptile Phylogeny and Reveal Unexpected Placements for Fossil Taxa. PLoS ONE. 2015; 10: e0118199 doi: 10.1371/journal.pone.0118199 25803280 10.1371/journal.pone.0118199 PMC4372529

[41] Seeley HG. On Remains of a small Lizard from the Neocomian Rocks of Comén, near Trieste preserved in the Geological Museum of the University of Vienna. Quarterly Journal of the Geological Society. 1881; 37: 52–6.

[42] Caldwell MW. On the aquatic squamate *Dolichosaurus longicollis* owen, 1850 (Cenomanian, Upper Cretaceous), and the evolution of elongate necks in squamates. J Vert Paleontol. 2000; 20: 720–35.

[43] Owen R. Description of the Fossil Reptiles of the Chalk Formation In: Dixon F, editor. The Geology and Fossils of the Tertiary and Cretaceous Formations of Sussex. London: Longman, Brown, Green and Longman,; 1850 pp. 378–404.

[44] Caldwell MW. A new species of *Pontosaurus* (Squamata, Pythonomorpha) from the Upper Cretaceous of Lebanon and a phylogenetic analysis of Pythonomorpha. Memorie della Società italiana di scienze naturali e Museo civico di storia naturale di Milano. 2006; 34: 1–43.

[45] Wiens JJ, Kuczynski CA, Townsend T, Reeder TW, Mulcahy DG, Sites JW. Combining phylogenomics and fossils in higher-level squamate reptile phylogeny: molecular data change the placement of fossil taxa. Syst Biol. 2010; 59: 674–88. doi: 10.1093/sysbio/syq048 20930035 10.1093/sysbio/syq048

[46] Fitch WM. Toward defining the course of evolution: minimum change for a specific tree topology. Syst Biol. 1971; 20: 406–16.

[47] Farris JS. Methods for computing Wagner trees. Syst Biol. 1970; 19: 83–92.

[48] Kramberger KG. *Aigialosaurus*, eine neue Eidechse aus den Kreideschiefern der Insel Lesina mit Rücksicht auf die bereits beschriebenen Lacertiden von Comen und Lesina. Glasnik huvatskoga naravolosovnoga derstva (Societas historico-matulis croatica) u Zagrebu. 1892; 7: 74–106.

[49] Caldwell MW, Dutchak AR. Redescription of *Aigialosaurus dalmaticus* Kramberger, 1892, a Cenomanian mosasauroid lizard from Hvar Island, Croatia. Can J Earth Sci. 2006; 43: 1821–34.

[50] Kornhuber A. *Opetiosaurus bucchichi*, eine neue fossile Eidechse aus der unteren Kreide von Lesina in Dalmatien. Abhandlungen der kaiserlich-königlichen geologischen Reichsanstalt zu Wien. 1901; 17: 1–24.

[51] Harrell J, TL, Martin J. A mosasaur from the Maastrichtian Fox Hills Formation of the northern Western Interior Seaway of the United States. Netherlands Journal of Geosciences—–Geologie en Mijnbouw. 2015; 94: 23–37.

[52] Mulder EWA. Transatlantic latest Cretaceous mosasaurs (Reptilia, Lacertilia) from the Maastrichtian type area and New Jersey. Geologie en Mijnbouw. 1999; 78: 281–300.

[53] Street HP, Caldwell MW. Rediagnosis and redescription of *Mosasaurus hoffmannii* (Squamata: Mosasauridae) and an assessment of species assigned to the genus *Mosasaurus*. Geological Magazine. 2016: 1–37.

[54] Konishi T, Caldwell MW. New specimens of *Platecarpus planifrons* (Cope, 1874) (Squamata: Mosasauridae) and a revised taxonomy of the genus. J Vert Paleontol. 2007; 27: 59–72.

[55] Konishi T, Caldwell MW. New material of the mosasaur *Plioplatecarpus nichollsae* Cuthbertson et al., 2007, clarifies problematic features of the holotype specimen. J Vert Paleontol. 2009; 29: 417–36.

[56] Konishi T, Caldwell MW. Two new plioplatecarpine (Squamata, Mosasauridae) genera from the Upper Cretaceous of North America, and a global phylogenetic analysis of plioplatecarpines. J Vert Paleontol. 2011; 31: 754–83.

[57] Bardet N, Suberbiola XP, Jalil N-E. A new mosasauroid (Squamata) from the Late Cretaceous (Turonian) of Morocco. Comptes Rendus Palevol. 2003; 2: 607–16.

[58] Goloboff PA, Farris JS, Nixon KC. TNT, a free program for phylogenetic analysis. Cladistics. 2008; 24: 774–86.

[59] Simões TR, Caldwell MW, Kellner AWA. A new Early Cretaceous lizard species from Brazil, and the phylogenetic position of the oldest known South American squamates. J Syst Palaeont. 2014.

[60] Goloboff PA. Estimating character weights during tree search. Cladistics. 1993; 9: 83–91. 10.1111/j.1096-0031.1993.tb00209.x 34929936

[61] Goloboff PA, Carpenter JM, Arias JS, Esquivel DRM. Weighting against homoplasy improves phylogenetic analysis of morphological data sets. Cladistics. 2008; 24: 758–73.

[62] Nguyen L-T, Schmidt HA, von Haeseler A, Minh BQ. IQ-TREE: A Fast and Effective Stochastic Algorithm for Estimating Maximum-Likelihood Phylogenies. Mol Biol Evol. 2015; 32: 268–74. doi: 10.1093/molbev/msu300 25371430 10.1093/molbev/msu300 PMC4271533

[63] Trifinopoulos J, Nguyen L-T, von Haeseler A, Minh BQ. W-IQ-TREE: a fast online phylogenetic tool for maximum likelihood analysis. Nucleic Acids Res. 2016. 10.1093/nar/gkw256 PMC4987875 27084950

[64] Yang Z. Maximum likelihood phylogenetic estimation from DNA sequences with variable rates over sites: approximate methods. J Mol Evol. 1994; 39: 306–14. 7932792 10.1007/BF00160154

[65] Minh BQ, Nguyen MAT, von Haeseler A. Ultrafast Approximation for Phylogenetic Bootstrap. Mol Biol Evol. 2013; 30: 1188–95. doi: 10.1093/molbev/mst024 23418397 10.1093/molbev/mst024 PMC3670741

[66] Ronquist F, Teslenko M, van der Mark P, Ayres DL, Darling A, Höhna S, et al MrBayes 3.2: efficient Bayesian phylogenetic inference and model choice across a large model space. Syst Biol. 2012; 61: 539–42. doi: 10.1093/sysbio/sys029 22357727 10.1093/sysbio/sys029 PMC3329765

[67] Wiens JJ, Bonett RM, Chippindale PT. Ontogeny discombobulates phylogeny: paedomorphosis and higher-level salamander relationships. Syst Biol. 2005; 54: 91–110. doi: 10.1080/10635150590906037 15805013 10.1080/10635150590906037

[68] Müller J, Reisz RR. The Phylogeny of Early Eureptiles: Comparing Parsimony and Bayesian Approaches in the Investigation of a Basal Fossil Clade. Syst Biol. 2006; 55: 503–11. doi: 10.1080/10635150600755396 16861212 10.1080/10635150600755396

[69] Ayache NC, Near TJ. The utility of morphological data in resolving phylogenetic relationships of darters as exemplified with *Etheostoma* (Teleostei: Percidae). Bull Peabody Mus Nat Hist. 2009; 50: 327–46.

[70] Prieto‐Márquez A. Global phylogeny of Hadrosauridae (Dinosauria: Ornithopoda) using parsimony and Bayesian methods. Zool J Linn Soc. 2010; 159: 435–502.

[71] Nylander JAA, Ronquist F, Huelsenbeck JP, Nieves-Aldrey J. Bayesian Phylogenetic Analysis of Combined Data. Syst Biol. 2004; 53: 47–67. 14965900 10.1080/10635150490264699

[72] Gelman A, Rubin DB. Inference from Iterative Simulation Using Multiple Sequences. Stat Sci. 1992; 7: 457–72.

[73] Rambaut A, Suchard MA, Xie D, Drummond AJ. Tracer v1.6, Available from http://beast.bio.ed.ac.uk/Tracer 2014.

[74] Rambaut A, Drummond AJ. TreeAnnotator v.2.4.3. 2016. Available from: http://beast.bio.ed.ac.uk/treeannotator.

[75] Templeton AR. Phylogenetic Inference From Restriction Endonuclease Cleavage Site Maps with Particular Reference to the Evolution of Humans and the Apes. Evolution. 1983; 37: 221–44. 10.1111/j.1558-5646.1983.tb05533.x 28568373

[76] Swofford DL. PAUP*. Phylogenetic Analysis Using Parsimony (* and Other Methods). Version 4.b10. Sunderland, MA: Sinauer Associates. 2002.

[77] Shimodaira H, Hasegawa M. Multiple Comparisons of Log-Likelihoods with Applications to Phylogenetic Inference. Mol Biol Evol. 1999; 16: 1114.

[78] Xie W, Lewis PO, Fan Y, Kuo L, Chen M-H. Improving marginal likelihood estimation for Bayesian phylogenetic model selection. Syst Biol. 2011; 60: 150–60. doi: 10.1093/sysbio/syq085 21187451 10.1093/sysbio/syq085 PMC3038348

[79] Ronquist F, Huelsenbeck J, Teslenko M. Draft MrBayes version 3.2 manual: tutorials and model summaries. 2011. Available from: http://mrbayes.sourceforge.net/manual.php.

[80] Fröbisch NB, Schoch RR. Testing the impact of miniaturization on phylogeny: Paleozoic dissorophoid amphibians. Syst Biol. 2009; 58: 312–27. doi: 10.1093/sysbio/syp029 20525586 10.1093/sysbio/syp029

[81] Kass RE, Raftery AE. Bayes factors. Journal of the american statistical association. 1995; 90: 773–95.

[82] Maddison WP, Maddison DR. Mesquite: a modular system for evolutionary analysis. Version 3.01. 2014. Available from: http://mesquiteproject.org.

[83] Leblanc ARH, Caldwell MW, Bardet N. A new mosasaurine from the Maastrichtian (Upper Cretaceous) phosphates of Morocco and its implications for mosasaurine systematics. J Vert Paleontol. 2012; 32: 82–104.

[84] Polcyn MJ, Bell GL. *Russellosaurus coheni* n. gen., n. sp., a 92 million-year-old mosasaur from Texas (USA), and the definition of the parafamily Russellosaurina. Netherlands Journal of Geosciences—Geologie en Mijnbouw. 2005; 84: 321–33.

[85] Cuthbertson RS, Mallon JC, Campione NE, Holmes RB. A new species of mosasaur (Squamata: Mosasauridae) from the Pierre Shale (lower Campanian) of Manitoba. Can J Earth Sci. 2007; 44: 593–606.

[86] Hawkins JA, Hughes CE, Scotland RW. Primary Homology Assessment, Characters and Character States. Cladistics. 1997; 13: 275–83. 10.1111/j.1096-0031.1997.tb00320.x 34911232

[87] Forey PL, Kitching I. Experiments in coding multistate characters In: Scotland RW, Pennington RT, editors. Homology and systematics: coding characters for phylogenetic analysis. London & New York: Taylor & Francis; 2000 pp. 54–80.

[88] Simões TR, Caldwell MW, Palci A, Nydam RL. Giant taxon-character matrices: quality of character constructions remains critical regardless of size. Cladistics. 2017; 33: 198–219. 10.1111/cla.12163 34710972

[89] Puttick MN, O'Reilly JE, Tanner AR, Fleming JF, Clark J, Holloway L, et al Uncertain-tree: discriminating among competing approaches to the phylogenetic analysis of phenotype data. Proc R Soc Lond, Ser B: Biol Sci. 2017; 284. 10.1098/rspb.2016.2290 PMC5247500 28077778

