# Supplementary figures and images for: Mosasauroid phylogeny under multiple phylogenetic methods provides new insights on the evolution of aquatic adaptations in the group
Source: PLoS One. 2017 May 3;12(5):e0176773. doi: 10.1371/journal.pone.0176773 (PMC5415187; doi:10.1371/journal.pone.0176773)

Exp+GA

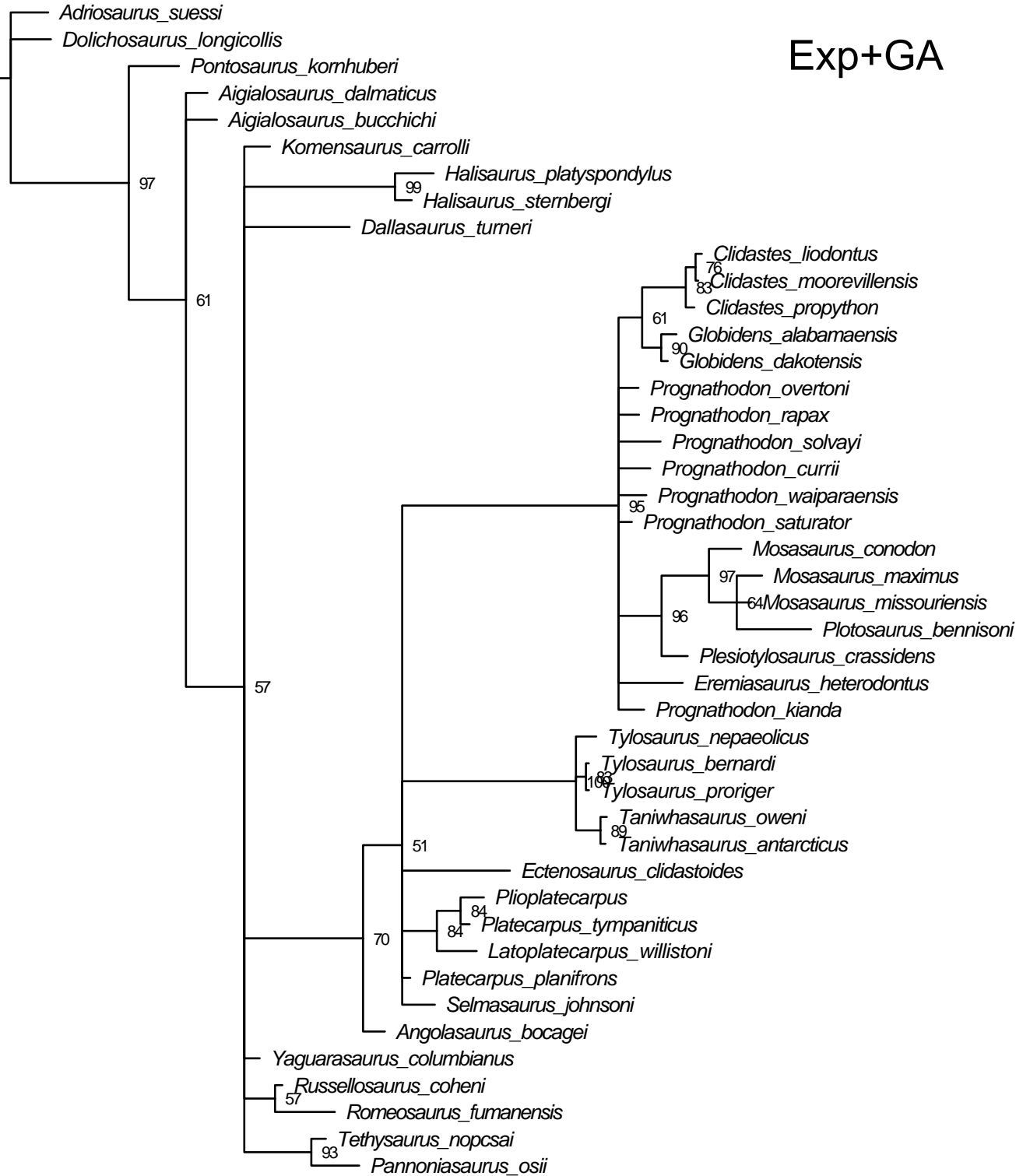

Exp+LN

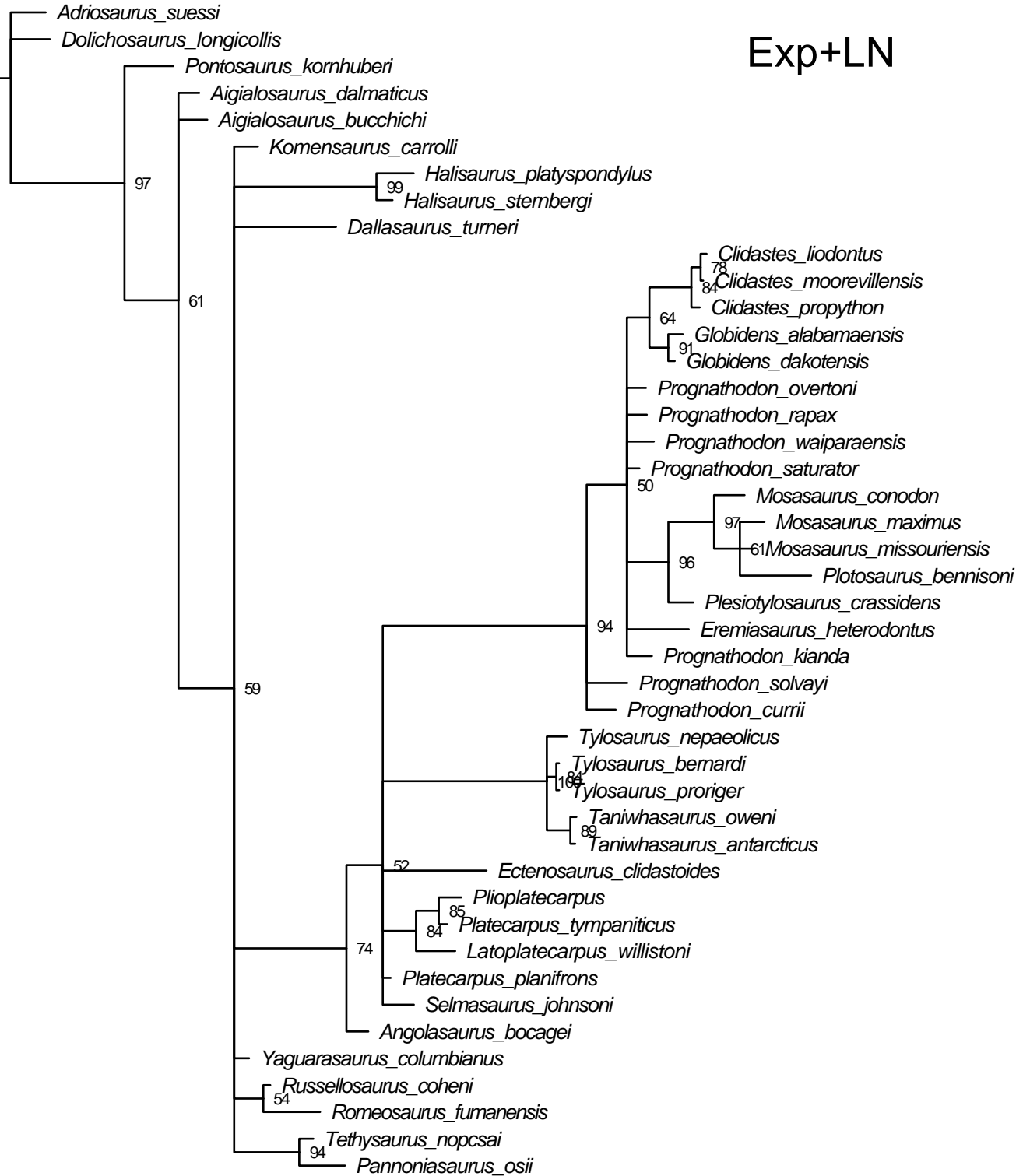

0.3

Uni+GA

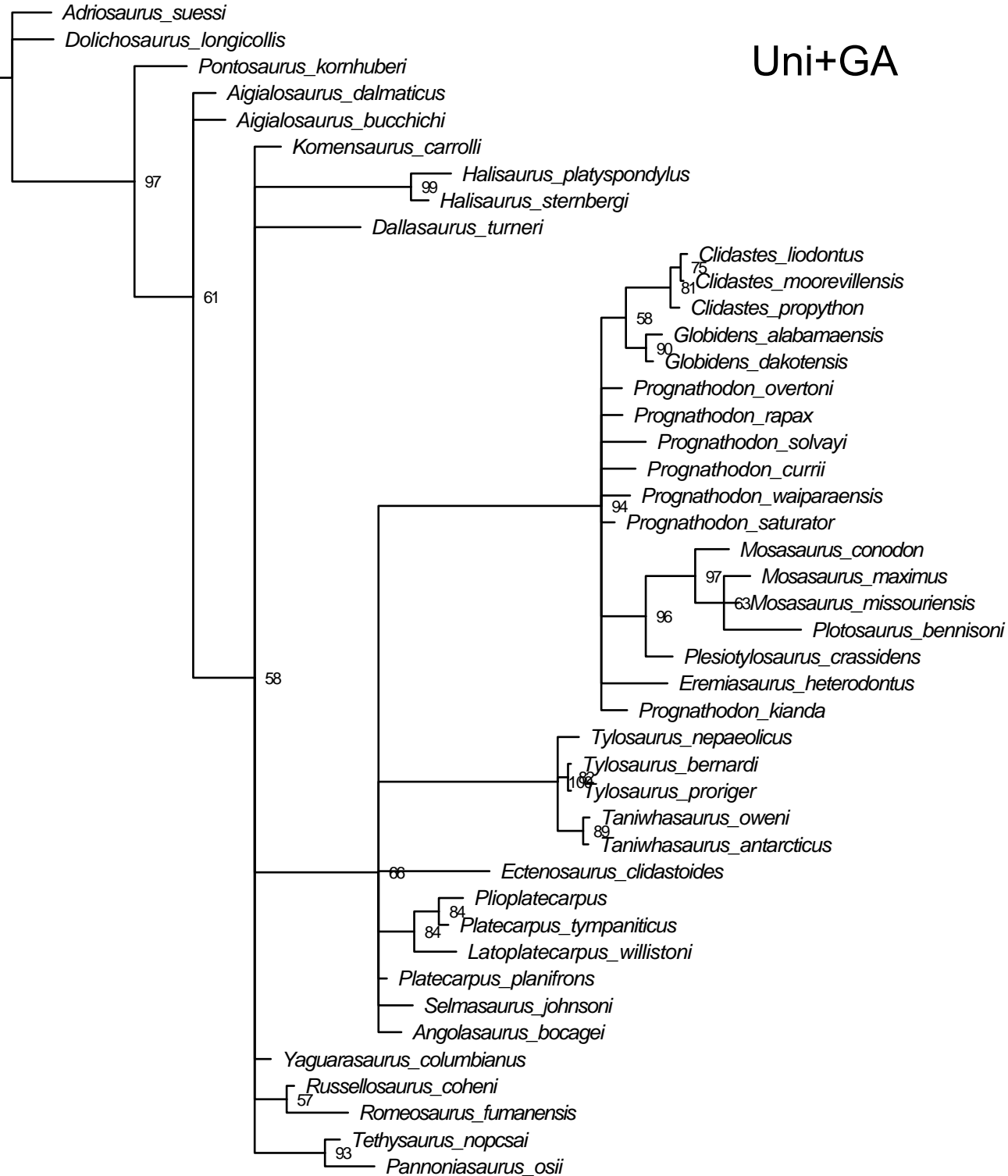

0.3

Uni+LN

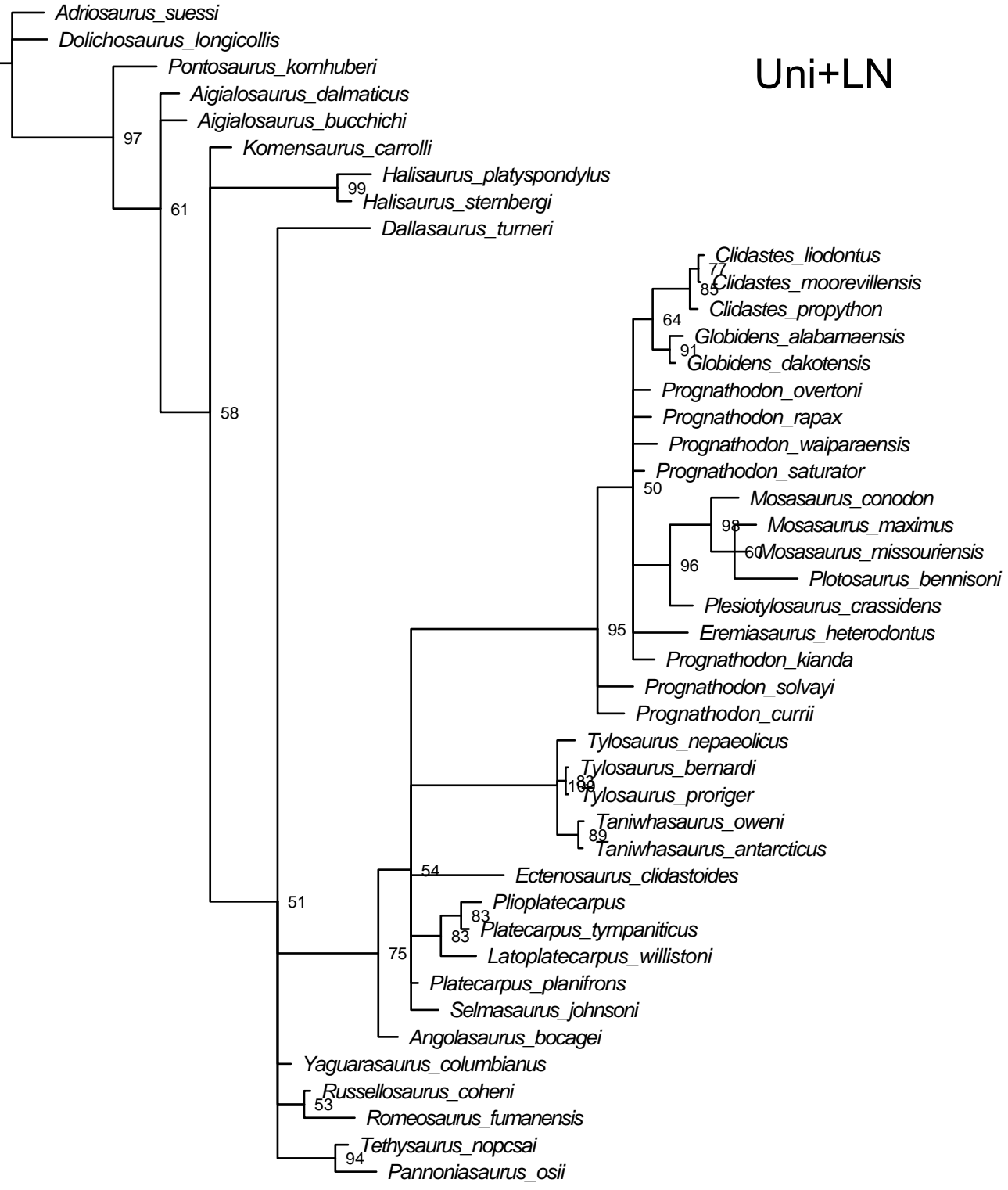

0.3

Supplement: S1 Fig — Majority rule consensus trees obtained from the four different prior and hyperprior choice combinations performed herein. (PDF) [file pone.0176773.s003.pdf]
