# Supplementary material for: Mosasauroid phylogeny under multiple phylogenetic methods provides new insights on the evolution of aquatic adaptations in the group
Source: PLoS One. 2017 May 3;12(5):e0176773. doi: 10.1371/journal.pone.0176773 (PMC5415187; doi:10.1371/journal.pone.0176773)

Co-UMP: Ch. 89

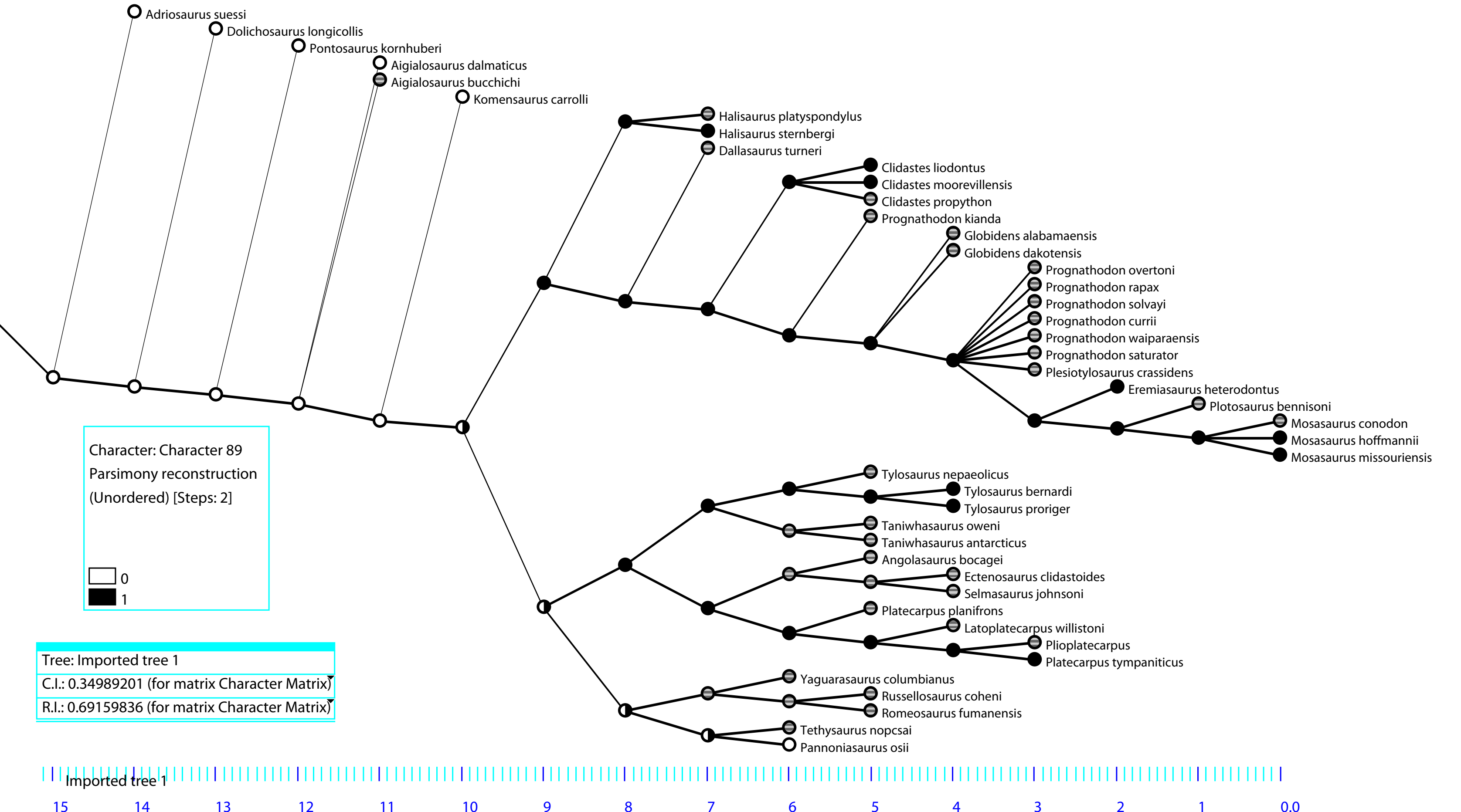
Adriosaurus suessi
Dolichosaurus longicollis
Pontosaurus kornhuberi
Aigialosaurus dalmaticus
Aigialosaurus bucchichi
Komensaurus carrolli
Halisaurus platyspondylus
Halisaurus sternbergi
Dallasaurus turneri
Clidastes liodontus
Clidastes moorevillensis
Clidastes propython
Prognathodon kianda
Globidens alabamaensis
Globidens dakotensis
Prognathodon overtoni
Prognathodon rapax
Prognathodon solvayi
Prognathodon currii
Prognathodon waiparaensis
Prognathodon saturator
Plesiotylosaurus crassidens
Eremiasaurus heterodontus
Plotosaurus bennisoni
Mosasaurus conodon
Mosasaurus hoffmannii
Mosasaurus missouriensis
Tylosaurus nepaeolicus
Tylosaurus bernardi
Tylosaurus proriger
Taniwhasaurus oweni
Taniwhasaurus antarcticus
Angolasaurus bocagei
Ectenosaurus clidastoides
Selmasaurus johnsoni
Platecarpus planifrons
Latoplatecarpus willistoni
Plioplatecarpus
Platecarpus tympaniticus
Yaguarasaurus columbianus
Russellosaurus coheni
Romeosaurus fumanensis
Tethysaurus nopcsai
Pannoniasaurus osii
Character: Character 89
Parsimony reconstruction
(Unordered) [Steps: 2]
0
1
Tree: Imported tree 1
C.I.: 0.34989201 (for matrix Character Matrix)
R.I.: 0.69159836 (for matrix Character Matrix)
Imported tree 1
15
14
13
12
11
10
9
8
7
6
5
4
3
2
1
0.0

Co-UMP: Ch. 117

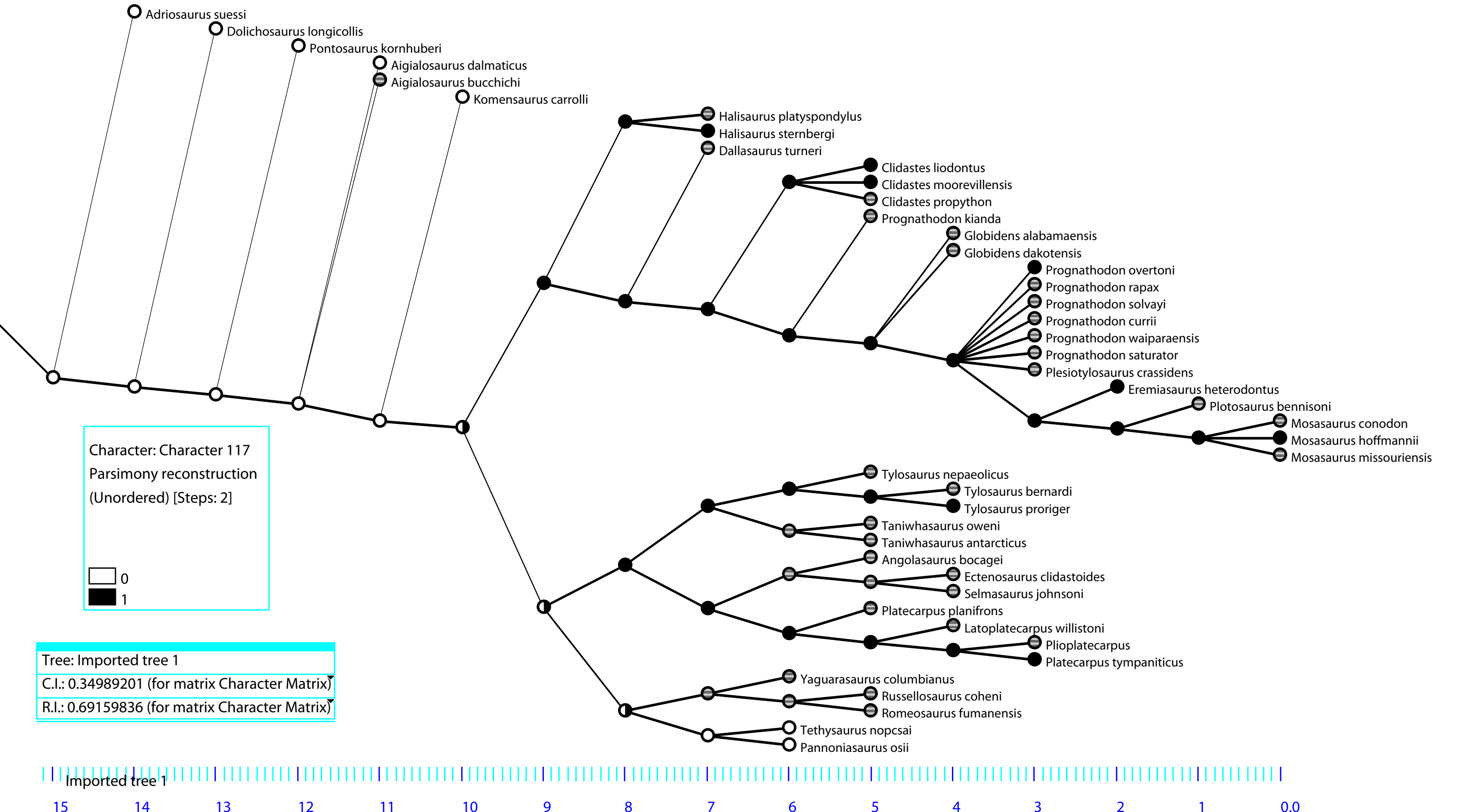

Adriosaurus suessi
Dolichosaurus longicollis
Pontosaurus kornhuberi
Aigialosaurus dalmaticus
Aigialosaurus bucchichi
Komensaurus carrolli
Halisaurus platyspondylus
Halisaurus sternbergi
Dallasaurus turneri
Clidastes liodontus
Clidastes moorevillensis
Clidastes propython
Prognathodon kianda
Globidens alabamaensis
Globidens dakotensis
Prognathodon overtoni
Prognathodon rapax
Prognathodon solvayi
Prognathodon currii
Prognathodon waiparaensis
Prognathodon saturator
Plesiotylosaurus crassidens
Eremiasaurus heterodontus
Plotosaurus bennisoni
Mosasaurus conodon
Mosasaurus hoffmannii
Mosasaurus missouriensis
Tylosaurus nepaeolicus
Tylosaurus bernardi
Tylosaurus proriger
Taniwhasaurus oweni
Taniwhasaurus antarcticus
Angolasaurus bocagei
Ectenosaurus clidastoides
Selmasaurus johnsoni
Platecarpus planifrons
Latoplatecarpus willistoni
Plioplatecarpus
Platecarpus tympaniticus
Yaguarasaurus columbianus
Russellosaurus coheni
Romeosaurus fumanensis
Tethysaurus nopcsai
Pannoniasaurus osii
Character: Character 117
Parsimony reconstruction
(Unordered) [Steps: 2]
0
1
Tree: Imported tree 1
C.I.: 0.34989201 (for matrix Character Matrix)
R.I.: 0.69159836 (for matrix Character Matrix)
Imported tree 1
15
14
13
12
11
10
9
8
7
6
5
4
3
2
1
0.0

Co-UMP: Ch. 123

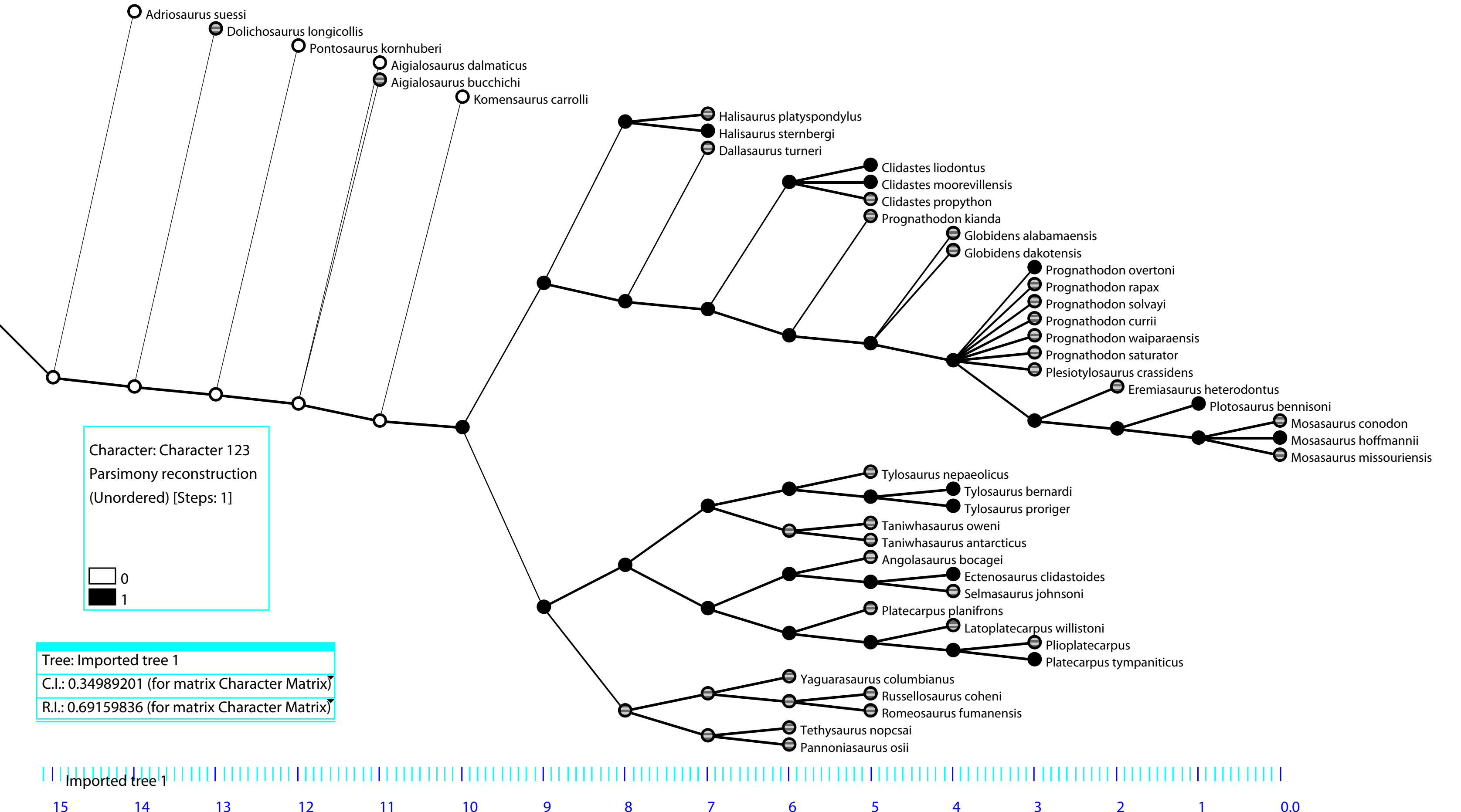

Adriosaurus suessi
Dolichosaurus longicollis
Pontosaurus kornhuberi
Aigialosaurus dalmaticus
Aigialosaurus bucchichi
Komensaurus carrolli
Halisaurus platyspondylus
Halisaurus sternbergi
Dallasaurus turneri
Clidastes liodontus
Clidastes moorevillensis
Clidastes propython
Prognathodon kianda
Globidens alabamaensis
Globidens dakotensis
Prognathodon overtoni
Prognathodon rapax
Prognathodon solvayi
Prognathodon currii
Prognathodon waiparaensis
Prognathodon saturator
Plesiotylosaurus crassidens
Eremiasaurus heterodontus
Plotosaurus bennisoni
Mosasaurus conodon
Mosasaurus hoffmannii
Mosasaurus missouriensis
Tylosaurus nepaeolicus
Tylosaurus bernardi
Tylosaurus proriger
Taniwhasaurus oweni
Taniwhasaurus antarcticus
Angolasaurus bocagei
Ectenosaurus clidastoides
Selmasaurus johnsoni
Platecarpus planifrons
Latoplatecarpus willistoni
Plioplatecarpus
Platecarpus tympaniticus
Yaguarasaurus columbianus
Russellosaurus coheni
Romeosaurus fumanensis
Tethysaurus nopcsai
Pannoniasaurus osii
Character: Character 123
Parsimony reconstruction
(Unordered) [Steps: 1]
0
1
Tree: Imported tree 1
C.I.: 0.34989201 (for matrix Character Matrix)
R.I.: 0.69159836 (for matrix Character Matrix)
Imported tree 1
15
14
13
12
11
10
9
8
7
6
5
4
3
2
1
0.0

Mu-UMP: Ch. 89

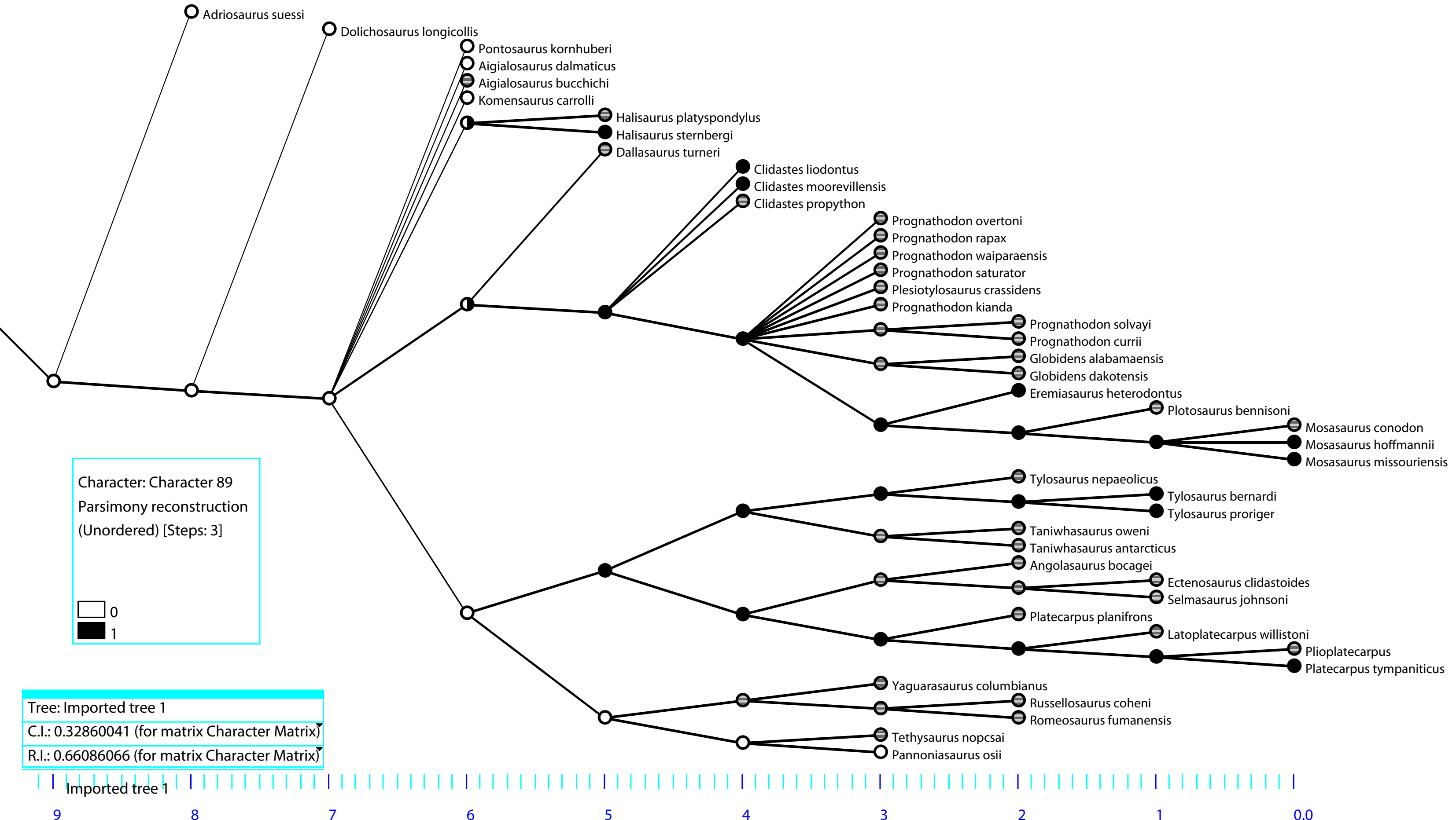

Adriosaurus suessi
Dolichosaurus longicollis
Pontosaurus kornhuberi
Aigialosaurus dalmaticus
Aigialosaurus bucchichi
Komensaurus carrolli
Halisaurus platyspondylus
Halisaurus sternbergi
Dallasaurus turneri
Clidastes liodontus
Clidastes moorevillensis
Clidastes propython
Prognathodon overtoni
Prognathodon rapax
Prognathodon waiparaensis
Prognathodon saturator
Plesiotylosaurus crassidens
Prognathodon kianda
Prognathodon solvayi
Prognathodon currii
Globidens alabamaensis
Globidens dakotensis
Eremiasaurus heterodontus
Plotosaurus bennisoni
Mosasaurus conodon
Mosasaurus hoffmannii
Mosasaurus missouriensis
Tylosaurus nepaeolicus
Tylosaurus bernardi
Tylosaurus proriger
Taniwhasaurus oweni
Taniwhasaurus antarcticus
Angolasaurus bocagei
Ectenosaurus clidastoides
Selmasaurus johnsoni
Platecarpus planifrons
Latoplatecarpus willistoni
Plioplatecarpus
Platecarpus tympaniticus
Yaguarasaurus columbianus
Russellosaurus coheni
Romeosaurus fumanensis
Tethysaurus nopcsai
Pannoniasaurus osii
Character: Character 89
Parsimony reconstruction
(Unordered) [Steps: 3]
0
1
Tree: Imported tree 1
C.I.: 0.32860041 (for matrix Character Matrix)
R.I.: 0.66086066 (for matrix Character Matrix)
Imported tree 1
9
8
7
6
5
4
3
2
1
0.0

Mu-UMP: Ch. 117

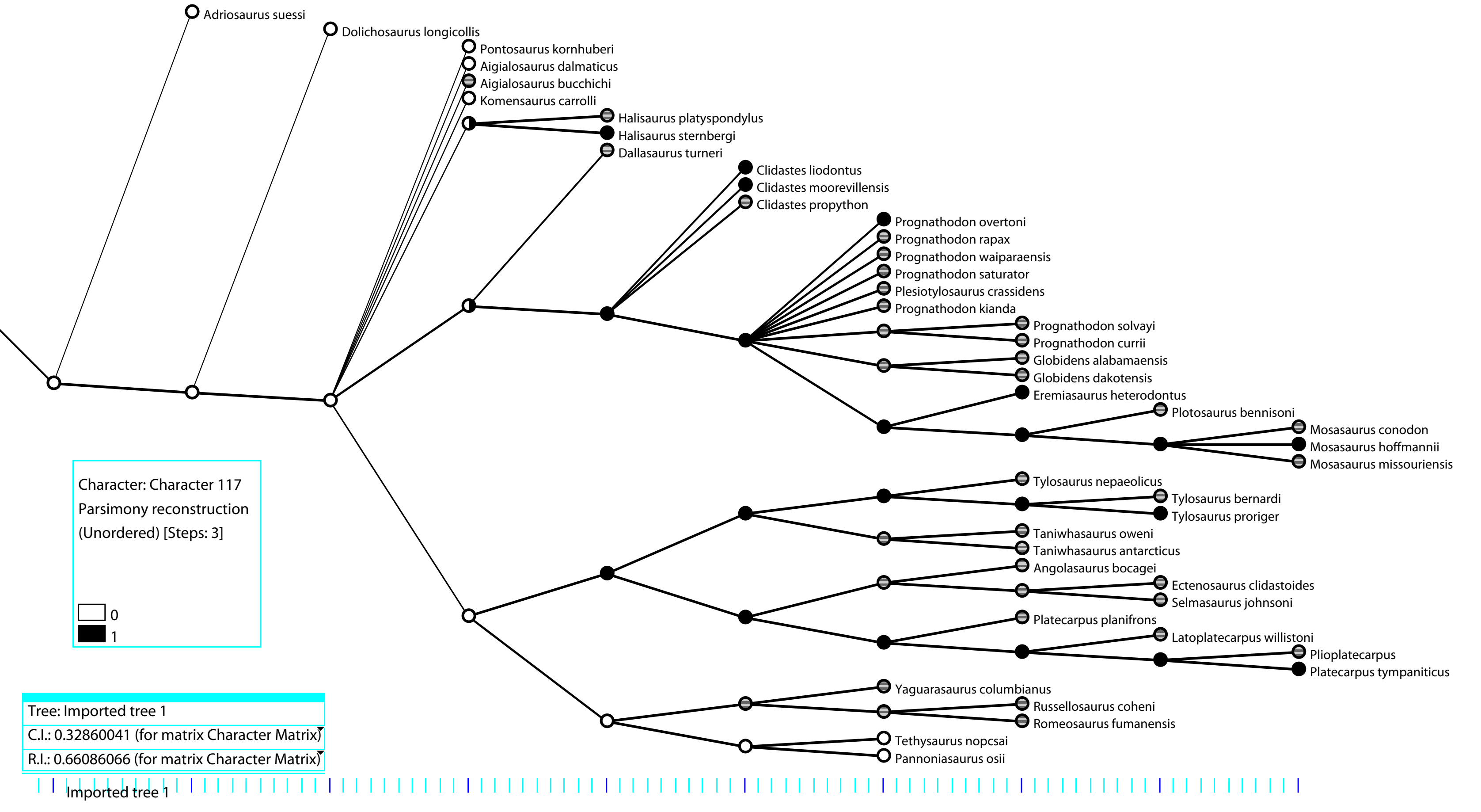

Adriosaurus suessi
Dolichosaurus longicollis
Pontosaurus kornhuberi
Aigialosaurus dalmaticus
Aigialosaurus bucchichi
Komensaurus carrolli
Halisaurus platyspondylus
Halisaurus sternbergi
Dallasaurus turneri
Clidastes liodontus
Clidastes moorevillensis
Clidastes propython
Prognathodon overtoni
Prognathodon rapax
Prognathodon waiparaensis
Prognathodon saturator
Plesiotylosaurus crassidens
Prognathodon kianda
Prognathodon solvayi
Prognathodon currii
Globidens alabamaensis
Globidens dakotensis
Eremiasaurus heterodontus
Plotosaurus bennisoni
Mosasaurus conodon
Mosasaurus hoffmannii
Mosasaurus missouriensis
Tylosaurus nepaeolicus
Tylosaurus bernardi
Tylosaurus proriger
Taniwhasaurus oweni
Taniwhasaurus antarcticus
Angolasaurus bocagei
Ectenosaurus clidastoides
Selmasaurus johnsoni
Platecarpus planifrons
Latoplatecarpus willistoni
Plioplatecarpus
Platecarpus tympaniticus
Yaguarasaurus columbianus
Russellosaurus coheni
Romeosaurus fumanensis
Tethysaurus nopcsai
Pannoniasaurus osii
Character: Character 117
Parsimony reconstruction
(Unordered) [Steps: 3]
0
1
Tree: Imported tree 1
C.I.: 0.32860041 (for matrix Character Matrix)
R.I.: 0.66086066 (for matrix Character Matrix)
Imported tree 1
9
8
7
6
5
4
3
2
1
0.0

Mu-UMP: Ch. 123

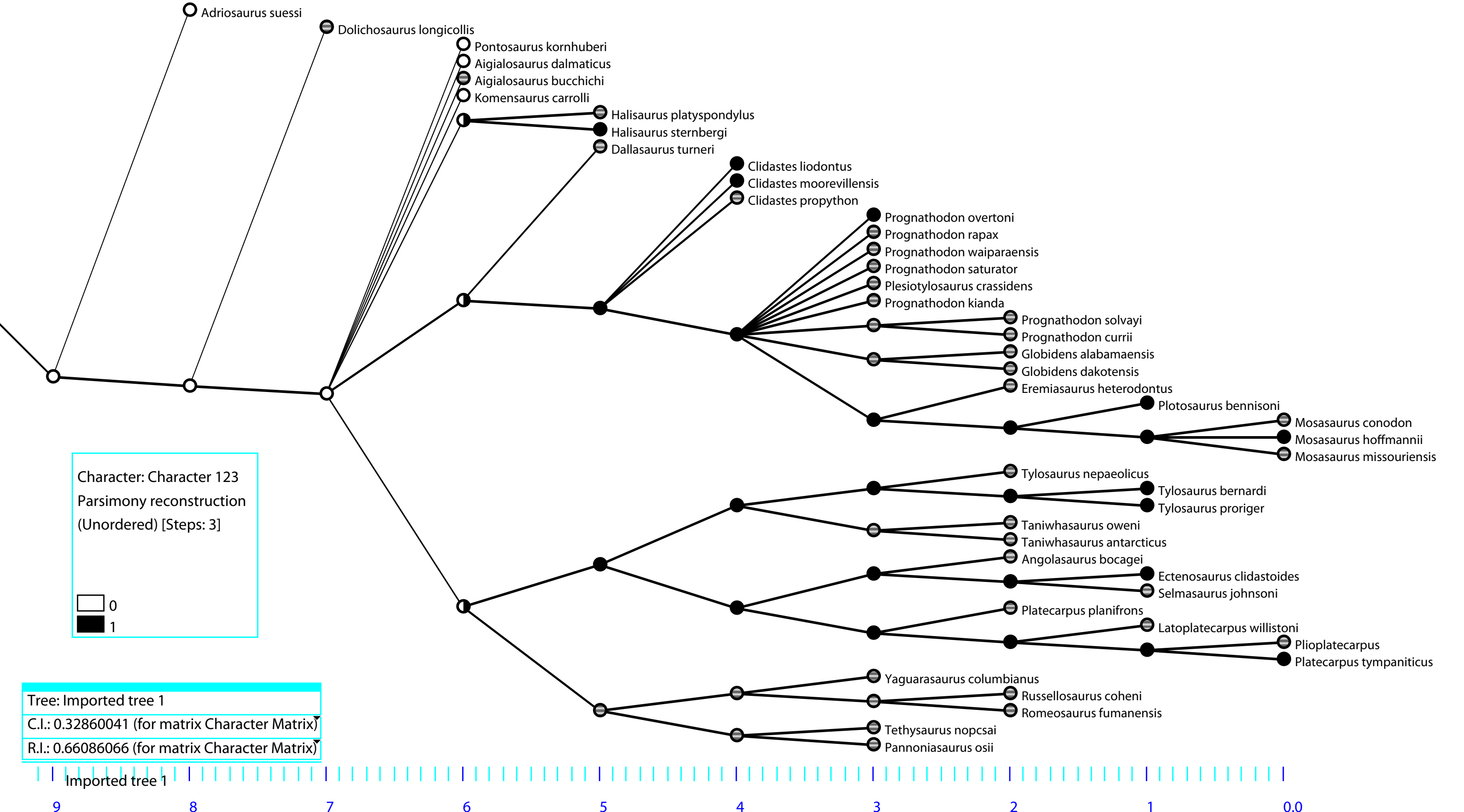
Adriosaurus suessi
Dolichosaurus longicollis
Pontosaurus kornhuberi
Aigialosaurus dalmaticus
Aigialosaurus bucchichi
Komensaurus carrolli
Halisaurus platyspondylus
Halisaurus sternbergi
Dallasaurus turneri
Clidastes liodontus
Clidastes moorevillensis
Clidastes propython
Prognathodon overtoni
Prognathodon rapax
Prognathodon waiparaensis
Prognathodon saturator
Plesiotylosaurus crassidens
Prognathodon kianda
Prognathodon solvayi
Prognathodon currii
Globidens alabamaensis
Globidens dakotensis
Eremiasaurus heterodontus
Plotosaurus bennisoni
Mosasaurus conodon
Mosasaurus hoffmannii
Mosasaurus missouriensis
Tylosaurus nepaeolicus
Tylosaurus bernardi
Tylosaurus proriger
Taniwhasaurus oweni
Taniwhasaurus antarcticus
Angolasaurus bocagei
Ectenosaurus clidastoides
Selmasaurus johnsoni
Platecarpus planifrons
Latoplatecarpus willistoni
Plioplatecarpus
Platecarpus tympaniticus
Yaguarasaurus columbianus
Russellosaurus coheni
Romeosaurus fumanensis
Tethysaurus nopcsai
Pannoniasaurus osii
Character: Character 123
Parsimony reconstruction
(Unordered) [Steps: 3]
0
1
Tree: Imported tree 1
C.I.: 0.32860041 (for matrix Character Matrix)
R.I.: 0.66086066 (for matrix Character Matrix)
Imported tree 1
9
8
7
6
5
4
3
2
1
0.0

IWMP: Ch. 89

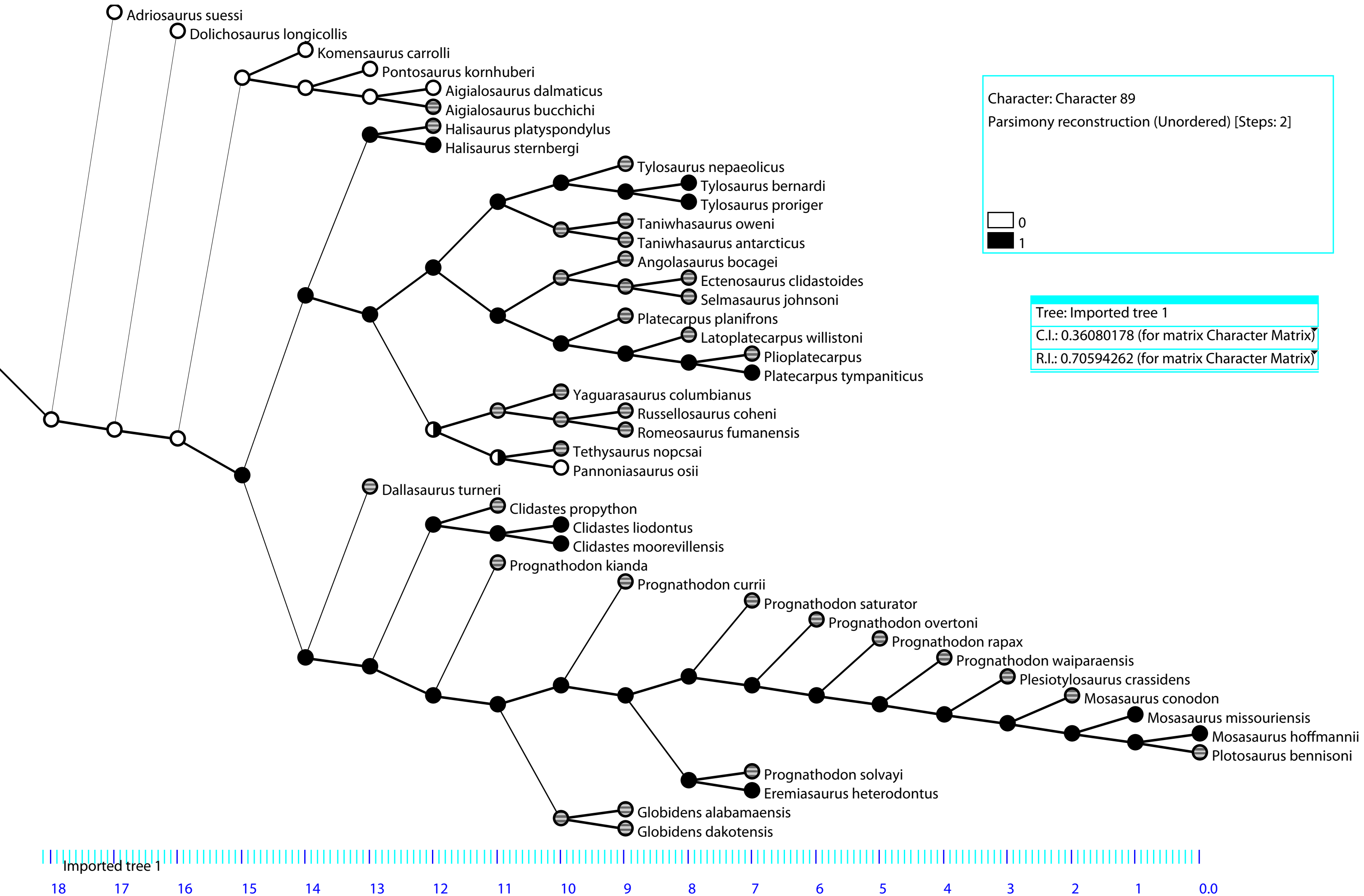

Adriosaurus suessi
Dolichosaurus longicollis
Komensaurus carrolli
Pontosaurus kornhuberi
Aigialosaurus dalmaticus
Aigialosaurus bucchichi
Halisaurus platyspondylus
Halisaurus sternbergi
Tylosaurus nepaeolicus
Tylosaurus bernardi
Tylosaurus proriger
Taniwhasaurus oweni
Taniwhasaurus antarcticus
Angolasaurus bocagei
Ectenosaurus clidastoides
Selmasaurus johnsoni
Platecarpus planifrons
Latoplatecarpus willistoni
Plioplatecarpus
Platecarpus tympaniticus
Yaguarasaurus columbianus
Russellosaurus coheni
Romeosaurus fumanensis
Tethysaurus nopcsai
Pannoniasaurus osii
Dallasaurus turneri
Clidastes propython
Clidastes liodontus
Clidastes moorevillensis
Prognathodon kianda
Prognathodon currii
Prognathodon saturator
Prognathodon overtoni
Prognathodon rapax
Prognathodon waiparaensis
Plesiotylosaurus crassidens
Mosasaurus conodon
Mosasaurus missouriensis
Mosasaurus hoffmannii
Plotosaurus bennisoni
Prognathodon solvayi
Eremiasaurus heterodontus
Globidens alabamaensis
Globidens dakotensis
Character: Character 89
Parsimony reconstruction (Unordered) [Steps: 2]
0
1
Tree: Imported tree 1
C.I.: 0.36080178 (for matrix Character Matrix)
R.I.: 0.70594262 (for matrix Character Matrix)
Imported tree 1
18
17
16
15
14
13
12
11
10
9
8
7
6
5
4
3
2
1
0.0

IWMP: Ch. 117

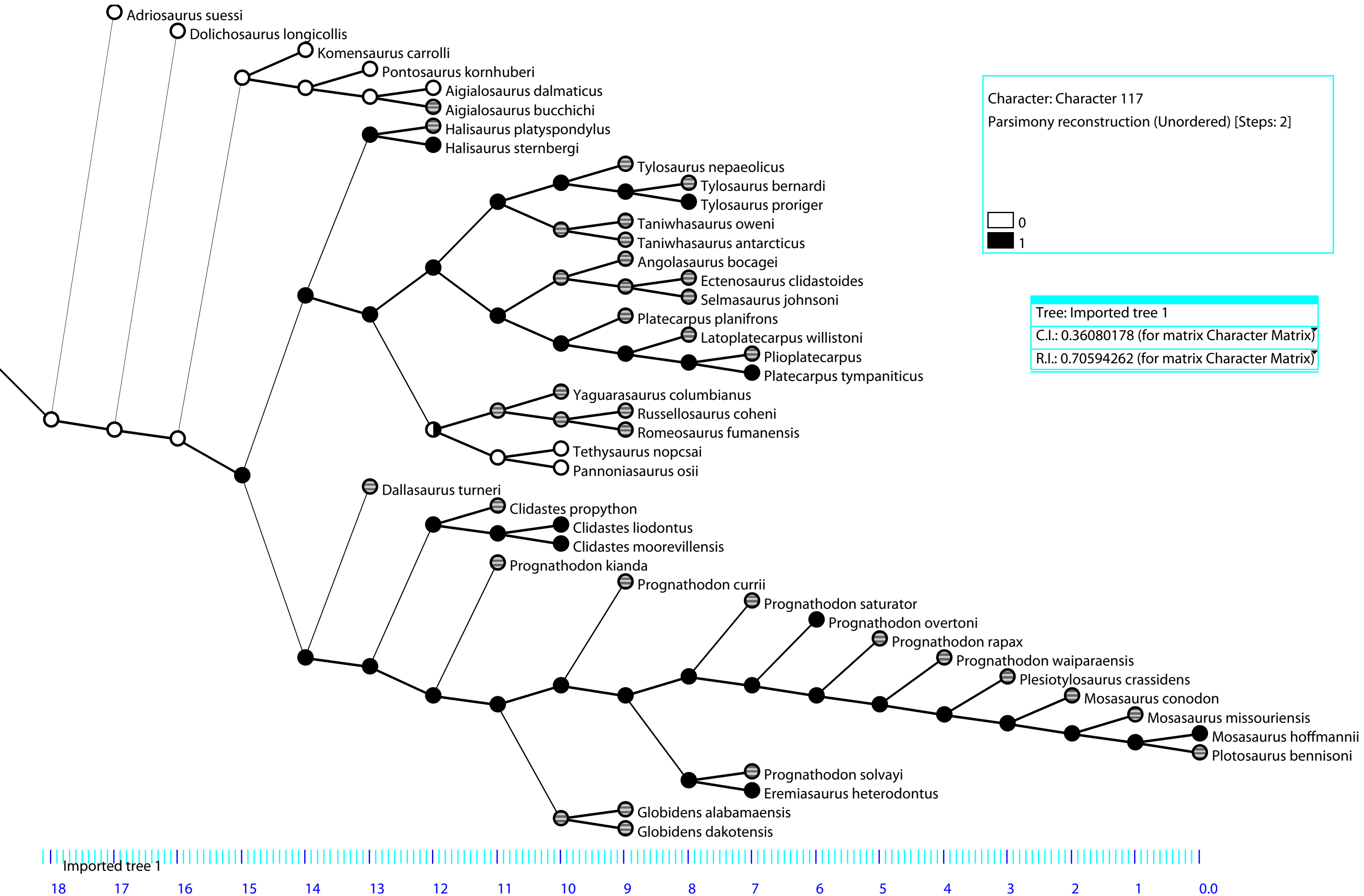
Adriosaurus suessi
Dolichosaurus longicollis
Komensaurus carrolli
Pontosaurus kornhuberi
Aigialosaurus dalmaticus
Aigialosaurus bucchichi
Halisaurus platyspondylus
Halisaurus sternbergi
Tylosaurus nepaeolicus
Tylosaurus bernardi
Tylosaurus proriger
Taniwhasaurus oweni
Taniwhasaurus antarcticus
Angolasaurus bocagei
Ectenosaurus clidastoides
Selmasaurus johnsoni
Platecarpus planifrons
Latoplatecarpus willistoni
Plioplatecarpus
Platecarpus tympaniticus
Yaguarasaurus columbianus
Russellosaurus coheni
Romeosaurus fumanensis
Tethysaurus nopcsai
Pannoniasaurus osii
Dallasaurus turneri
Clidastes propython
Clidastes liodontus
Clidastes moorevillensis
Prognathodon kianda
Prognathodon currii
Prognathodon saturator
Prognathodon overtoni
Prognathodon rapax
Prognathodon waiparaensis
Plesiotylosaurus crassidens
Mosasaurus conodon
Mosasaurus missouriensis
Mosasaurus hoffmannii
Plotosaurus bennisoni
Prognathodon solvayi
Eremiasaurus heterodontus
Globidens alabamaensis
Globidens dakotensis
Character: Character 117
Parsimony reconstruction (Unordered) [Steps: 2]
0
1
Tree: Imported tree 1
C.I.: 0.36080178 (for matrix Character Matrix)
R.I.: 0.70594262 (for matrix Character Matrix)
Imported tree 1
18
17
16
15
14
13
12
11
10
9
8
7
6
5
4
3
2
1
0.0

IWMP: Ch. 123

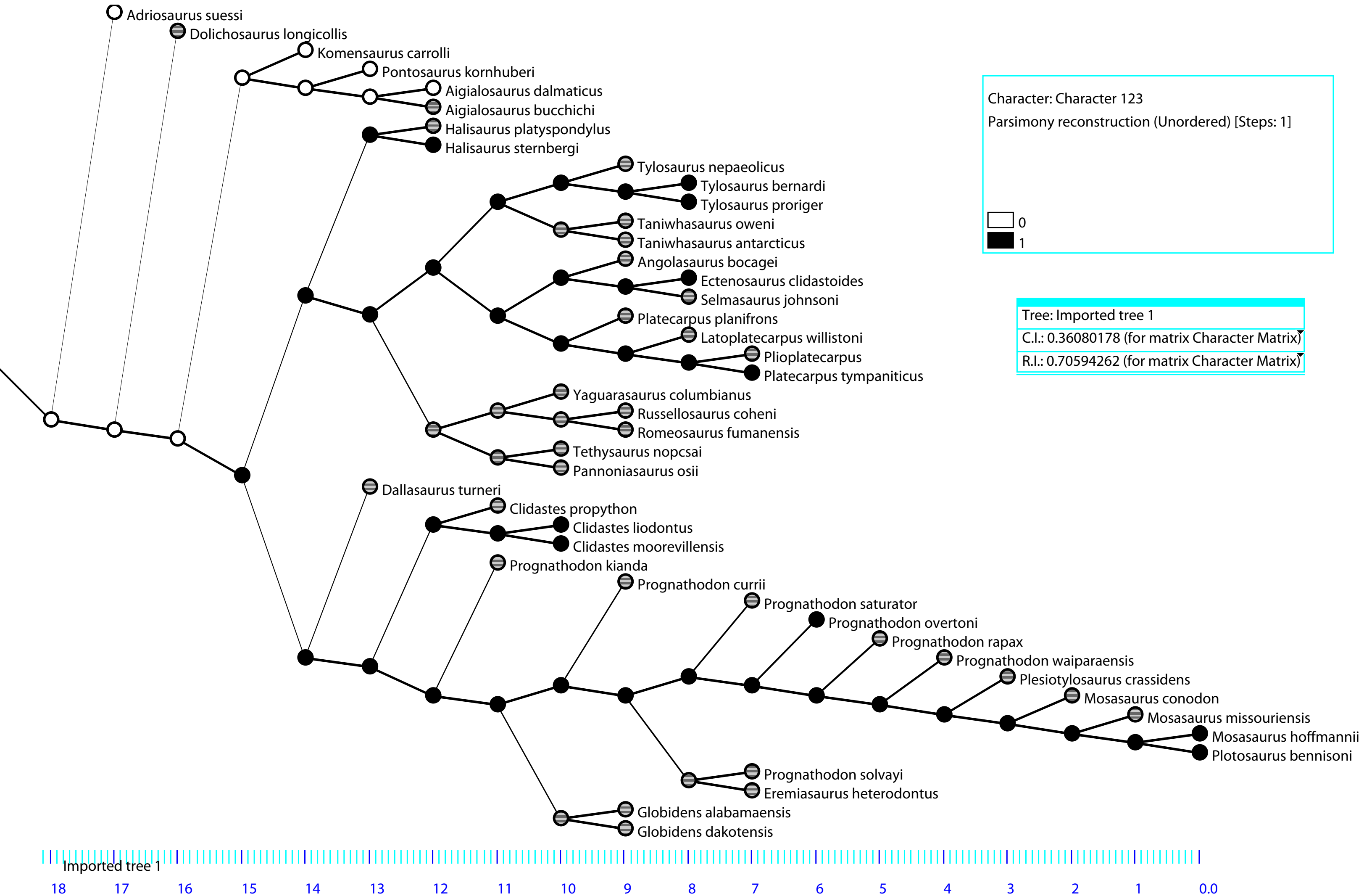

Adriosaurus suessi
Dolichosaurus longicollis
Komensaurus carrolli
Pontosaurus kornhuberi
Aigialosaurus dalmaticus
Aigialosaurus bucchichi
Halisaurus platyspondylus
Halisaurus sternbergi
Tylosaurus nepaeolicus
Tylosaurus bernardi
Tylosaurus proriger
Taniwhasaurus oweni
Taniwhasaurus antarcticus
Angolasaurus bocagei
Ectenosaurus clidastoides
Selmasaurus johnsoni
Platecarpus planifrons
Latoplatecarpus willistoni
Plioplatecarpus
Platecarpus tympaniticus
Yaguarasaurus columbianus
Russellosaurus coheni
Romeosaurus fumanensis
Tethysaurus nopcsai
Pannoniasaurus osii
Dallasaurus turneri
Clidastes propython
Clidastes liodontus
Clidastes moorevillensis
Prognathodon kianda
Prognathodon currii
Prognathodon saturator
Prognathodon overtoni
Prognathodon rapax
Prognathodon waiparaensis
Plesiotylosaurus crassidens
Mosasaurus conodon
Mosasaurus missouriensis
Mosasaurus hoffmannii
Plotosaurus bennisoni
Prognathodon solvayi
Eremiasaurus heterodontus
Globidens alabamaensis
Globidens dakotensis
Character: Character 123
Parsimony reconstruction (Unordered) [Steps: 1]
0
1
Tree: Imported tree 1
C.I.: 0.36080178 (for matrix Character Matrix)
R.I.: 0.70594262 (for matrix Character Matrix)
Imported tree 1
18
17
16
15
14
13
12
11
10
9
8
7
6
5
4
3
2
1
0.0

ML: Ch. 89

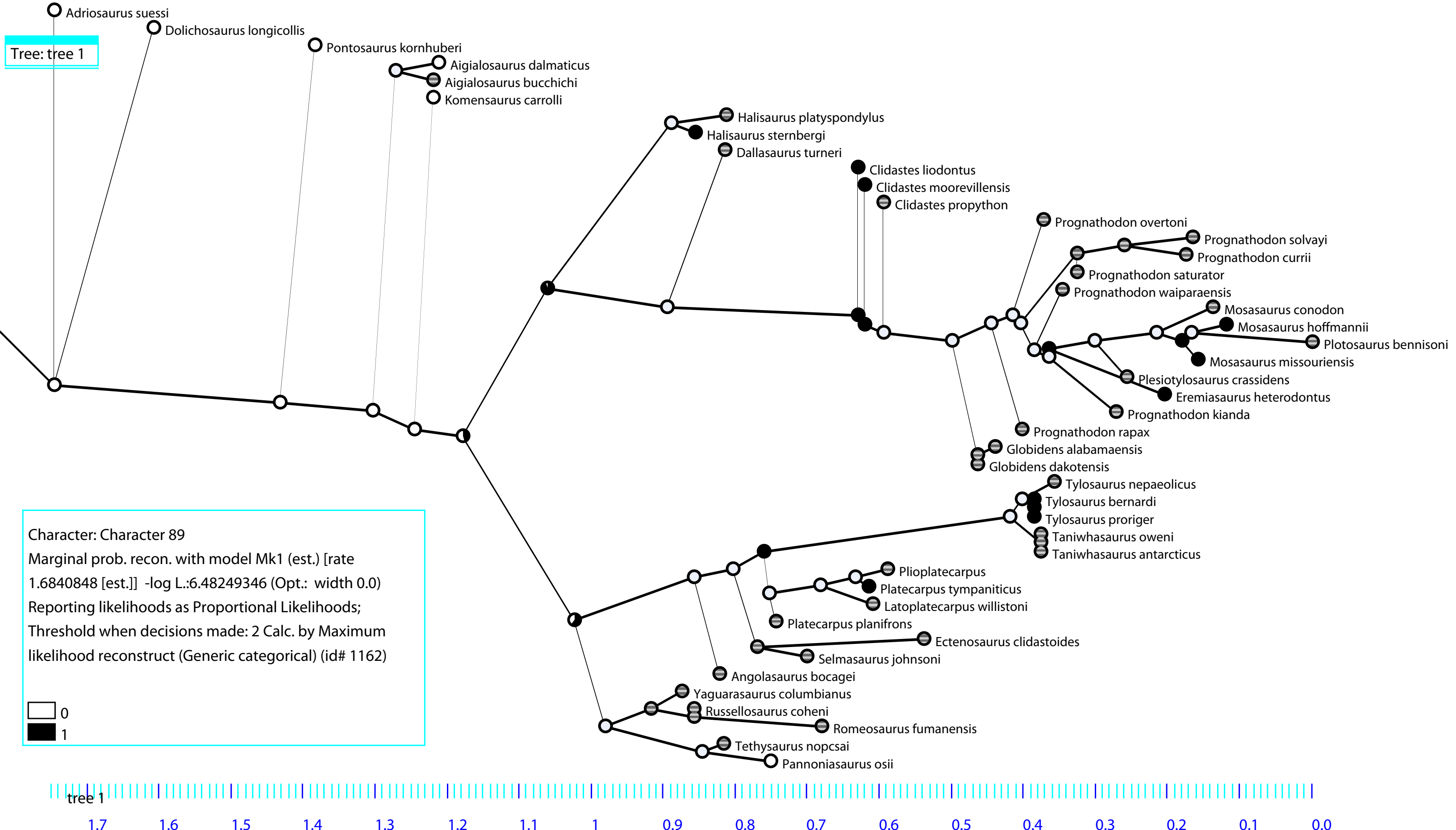

Character: Character 89
Marginal prob. recon. with model Mk1 (est.) [rate 1.6840848 [est.]] -log L.:6.48249346 (Opt.: width 0.0)
Reporting likelihoods as Proportional Likelihoods;
Threshold when decisions made: 2 Calc. by Maximum likelihood reconstruct (Generic categorical) (id# 1162)

0
1

ML: Ch. 117

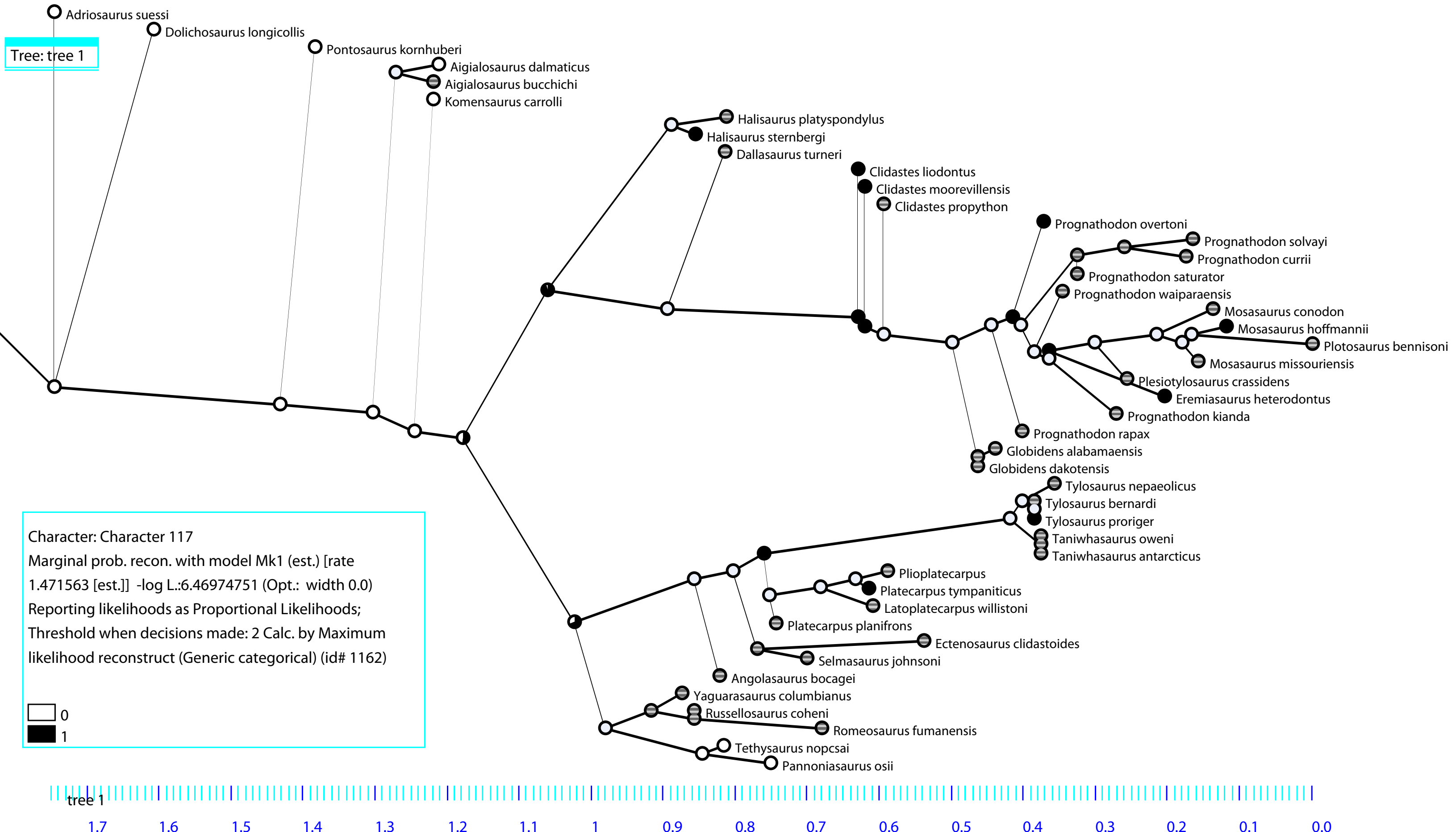

Tree: tree 1
Adriosaurus suessi
Dolichosaurus longicollis
Pontosaurus kornhuberi
Aigialosaurus dalmaticus
Aigialosaurus bucchichi
Komensaurus carrolli
Halisaurus platyspondylus
Halisaurus sternbergi
Dallasaurus turneri
Clidastes liodontus
Clidastes moorevillensis
Clidastes propython
Prognathodon overtoni
Prognathodon solvayi
Prognathodon currii
Prognathodon saturator
Prognathodon waiparaensis
Mosasaurus conodon
Mosasaurus hoffmannii
Plotosaurus bennisoni
Mosasaurus missouriensis
Plesiotylosaurus crassidens
Eremiasaurus heterodontus
Prognathodon kianda
Prognathodon rapax
Globidens alabamaensis
Globidens dakotensis
Tylosaurus nepaeolicus
Tylosaurus bernardi
Tylosaurus proriger
Taniwhasaurus oweni
Taniwhasaurus antarcticus
Plioplatecarpus
Platecarpus tympaniticus
Latoplatecarpus willistoni
Platecarpus planifrons
Ectenosaurus clidastoides
Selmasaurus johnsoni
Angolasaurus bocagei
Yaguarasaurus columbianus
Russellosaurus coheni
Romeosaurus fumanensis
Tethysaurus nopcsai
Pannoniasaurus osii
Character: Character 117
Marginal prob. recon. with model Mk1 (est.) [rate 1.471563 [est.]] -log L.:6.46974751 (Opt.: width 0.0)
Reporting likelihoods as Proportional Likelihoods;
Threshold when decisions made: 2 Calc. by Maximum likelihood reconstruct (Generic categorical) (id# 1162)
0
1
tree 1
1.7
1.6
1.5
1.4
1.3
1.2
1.1
1
0.9
0.8
0.7
0.6
0.5
0.4
0.3
0.2
0.1
0.0

ML: Ch. 123

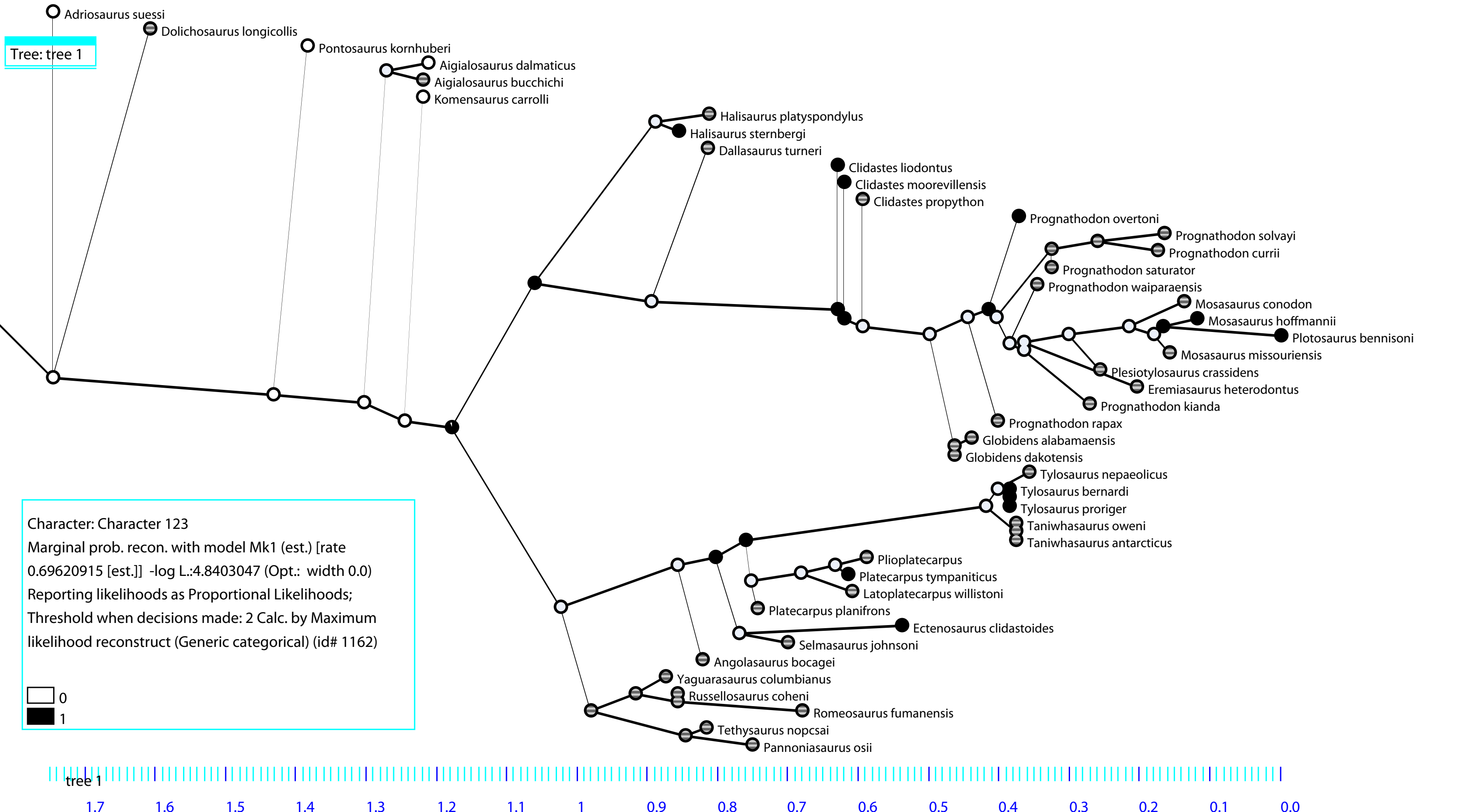
Adriosaurus suessi
Dolichosaurus longicollis
Tree: tree 1
Pontosaurus kornhuberi
Aigialosaurus dalmaticus
Aigialosaurus bucchichi
Komensaurus carrolli
Halisaurus platyspondylus
Halisaurus sternbergi
Dallasaurus turneri
Clidastes liodontus
Clidastes moorevillensis
Clidastes propython
Prognathodon overtoni
Prognathodon solvayi
Prognathodon currii
Prognathodon saturator
Prognathodon waiparaensis
Mosasaurus conodon
Mosasaurus hoffmannii
Plotosaurus bennisoni
Mosasaurus missouriensis
Plesiotylosaurus crassidens
Eremiasaurus heterodontus
Prognathodon kianda
Prognathodon rapax
Globidens alabamaensis
Globidens dakotensis
Tylosaurus nepaeolicus
Tylosaurus bernardi
Tylosaurus proriger
Taniwhasaurus oweni
Taniwhasaurus antarcticus
Plioplatecarpus
Platecarpus tympaniticus
Latoplatecarpus willistoni
Platecarpus planifrons
Ectenosaurus clidastoides
Selmasaurus johnsoni
Angolasaurus bocagei
Yaguarasaurus columbianus
Russellosaurus coheni
Romeosaurus fumanensis
Tethysaurus nopcsai
Pannoniasaurus osii
Character: Character 123
Marginal prob. recon. with model Mk1 (est.) [rate 0.69620915 [est.]] -log L.:4.8403047 (Opt.: width 0.0) Reporting likelihoods as Proportional Likelihoods; Threshold when decisions made: 2 Calc. by Maximum likelihood reconstruct (Generic categorical) (id# 1162)
0
1
tree 1
1.7
1.6
1.5
1.4
1.3
1.2
1.1
1
0.9
0.8
0.7
0.6
0.5
0.4
0.3
0.2
0.1
0.0

BI-MaxCladeCred: Ch.89

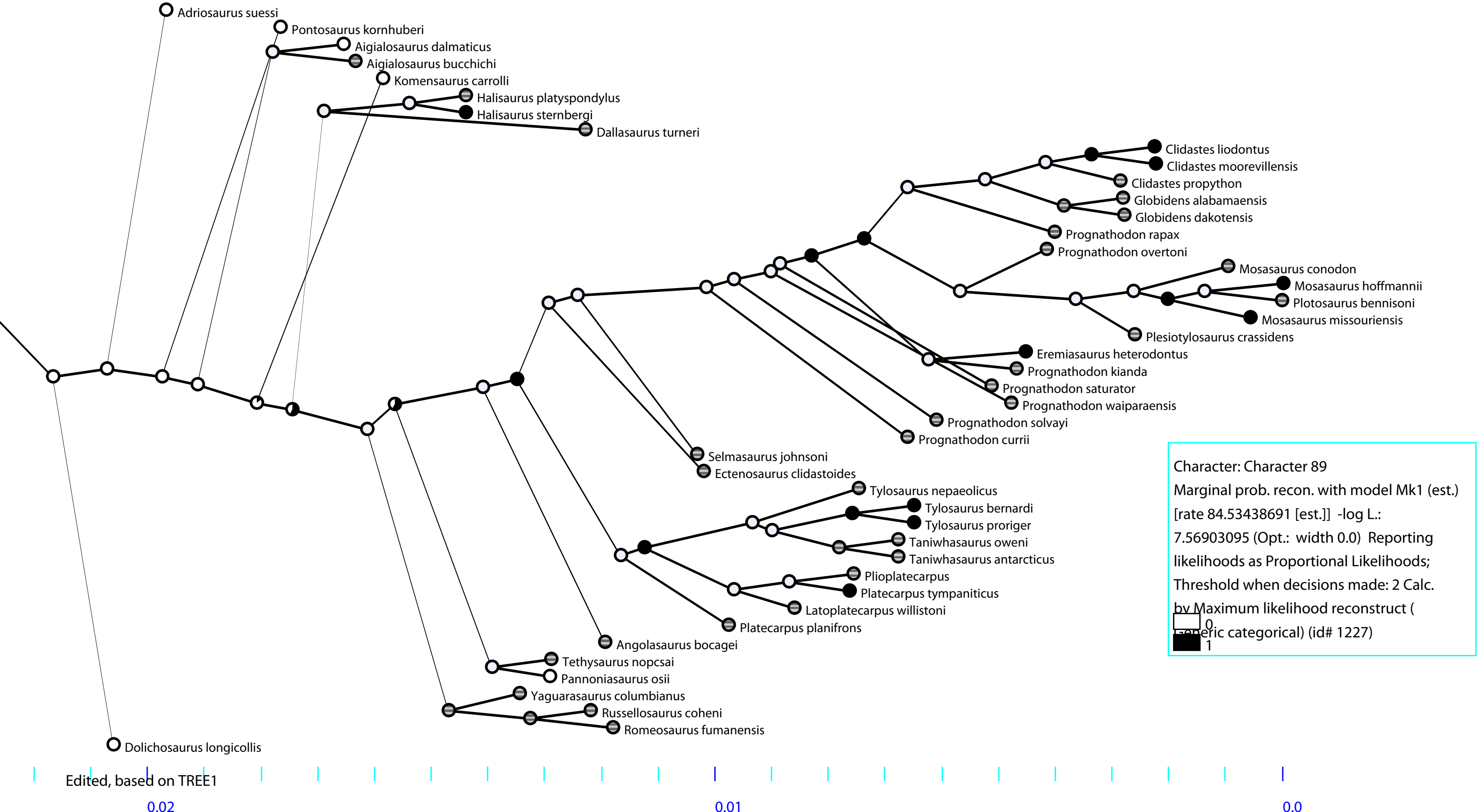

BI-MaxCladeCred: Ch.117

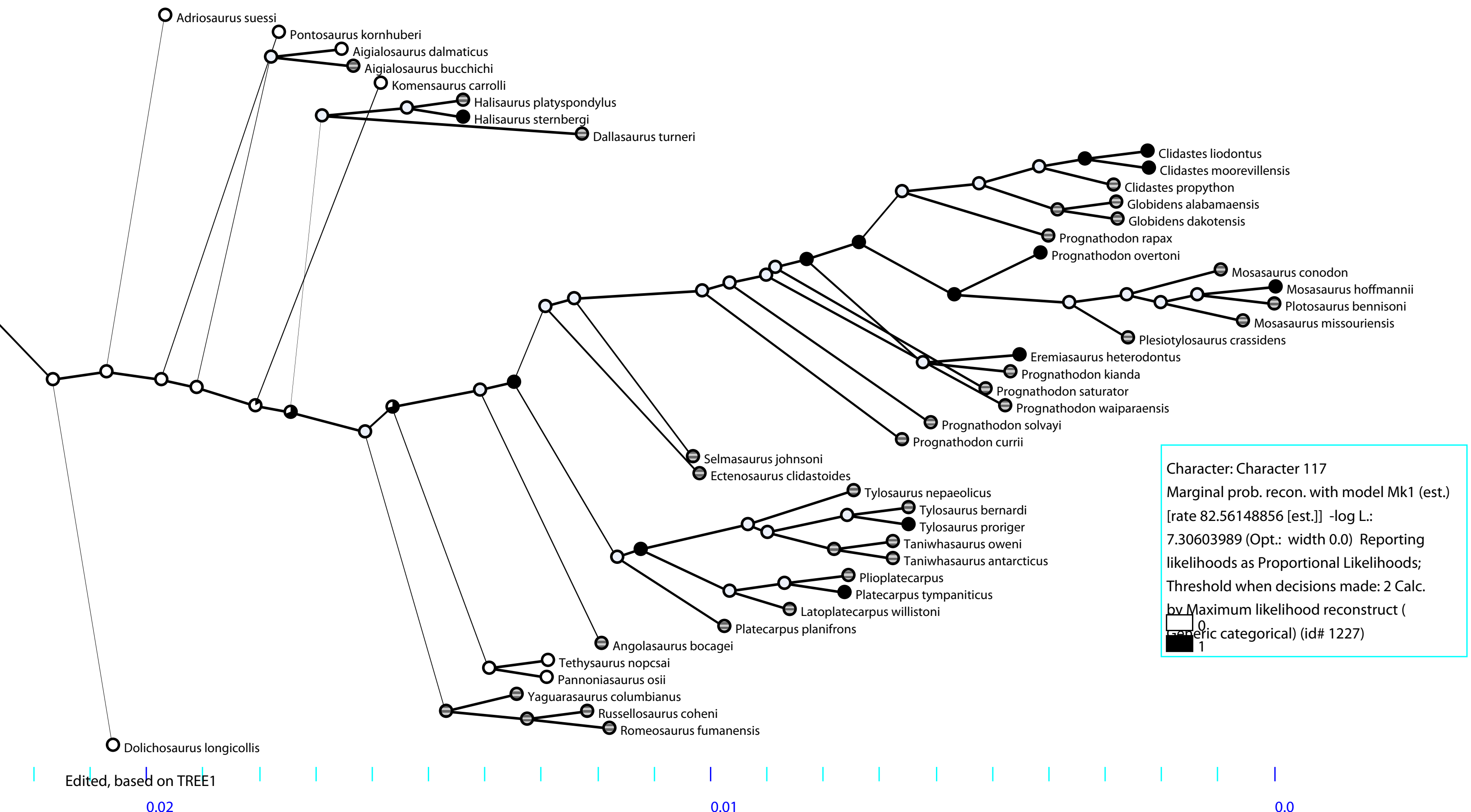

BI-MaxCladeCred: Ch.123

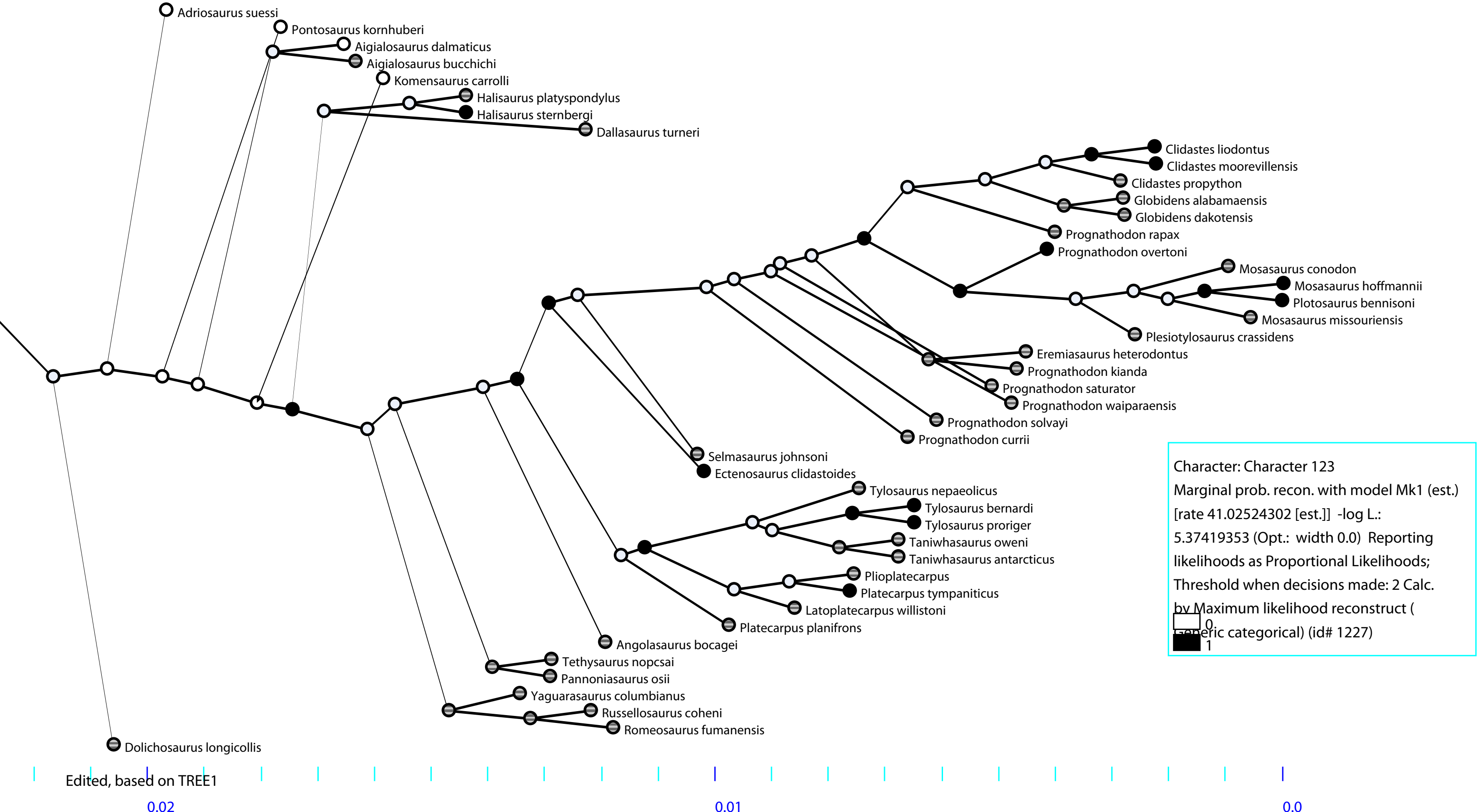

Supplement: S2 Fig — (PDF) [file pone.0176773.s004.pdf]
