# Supplementary material for: Mosasauroid phylogeny under multiple phylogenetic methods provides new insights on the evolution of aquatic adaptations in the group
Source: PLoS One. 2017 May 3;12(5):e0176773. doi: 10.1371/journal.pone.0176773 (PMC5415187; doi:10.1371/journal.pone.0176773)
Supplement: S1 Text — (DOCX) [file pone.0176773.s005.docx]

**Supporting Information**

Mosasauroid phylogeny under multiple phylogenetic methods provides new insights on the evolution of aquatic adaptations in the group

Tiago R. Simões*^1^, Oksana Vernygora^1^, Ilaria Paparella^1^, Paulina Jimenez-Huidobro^1^ and Michael W. Caldwell^1,2^

**S1 Text.**

Description of the characters used in phylogenetic analysis, taken from Palci et al. (2013) and Jimenez-Huidobro (2016).

Part I includes characters with multistate coding scheme and Part II includes the same character list, but using a contingent coding scheme. See Part I description below for details.

**Part I. Character list applying multistate character coding**

- 1. **Merged characters**

Character numbers from Jimenez-Huidobro et al. (2016): 1+2 (new character 1), 15+16 (new character 14), 28+29 (new character 26), 34+35 (new character 31), 58+59 (new character 54), 93+94 (new character 88).

* characters 42 to 44 are dependent on character 41, but it is not possible to merge all of them into a single multistate character. Old characters 17 already is a multistate character, and merging with character 18 would mix two different properties (types of frontal invasions with the extent of such elongations). The same applies to characters 77+78, and 102+103. We have left these characters unaltered.

**1.2 Additional character states**

Some additional character states had to be added to six characters in order to account for the variation represented by the newly included taxa. The numbers of the new character states in our new list of characters is: 37 (3); 63 (2); 100 (2).

**2. Character list with updated character numbers following changes described above.**

1. Premaxilla predental rostrum II: rostrum absent (0); rostrum very short and obtuse (1); or distinctly protruding (2); or very large and inflated (3). In *Clidastes* a short, acute, protruding rostrum (state 1) produces a ‘V’-shaped dorsal profile and, as far as is known, is peculiar to that genus. An alternative condition, described as ‘U’- shaped, includes those taxa whose rostral conditions span the whole range of states of characters 1 and 2. Hence, the descriptive character is abandoned in favor of a more informative structure-based series.
2. Premaxilla shape: bone broadly arcuate anteriorly (0); or relatively narrowly arcuate or acute anteriorly (1). In virtually all lizards the premaxilla is a very widely arcuate and lightly constructed element, and the base of the internarial process is quite narrow as in Aigialosaurus bucchichi. All other mosasaurids have a very narrowed premaxilla with the teeth forming a tight curve and the internarial process being proportionally wider (state 1). *Tethysaurus* was recoded as having state 0.
3. Premaxilla internarial bar width: narrow, distinctly less than half of the maximum width of the rostrum in dorsal view (0); or wide, being barely narrower than the rostrum (1). *Aigialosaurus* was recoded as having state 0.
4. Premaxilla internarial bar base shape: triangular (0); or rectangular (1). A vertical cross-section through the junction of the internarial bar and the dentigerous rostrum produces an inverted triangle in most taxa. But in state 1, this cross-section is transversely rectangular because the broad ventral surface of the bar is planar.
5. Premaxilla internarial bar dorsal keel: absent (0); or present (1). In state 1 a ridge rises above the level of a normally smoothly continuous transverse arch formed by the bones of the anterior muzzle.
6. Premaxilla internarial bar venter: with entrance for the fifth cranial nerve close to rostrum (0); or far removed from rostrum (1). The conduit that marks the path of the fifth cranial nerve from the maxilla into the premaxilla is expressed as a ventrolateral foramen within the premaxillo-maxillary sutural surface at the junction of the internarial bar and the dentigerous rostrum. State 1 includes a long shallow groove on the ventral surface of the bar. Anteriorly, this groove becomes a tunnel entering the bone at an extremely shallow angle, but disappearing below the surface at least 1 cm behind the rostrum.
7. Frontal shape in front of the orbits: sides sinusoidal (0); or bone nearly triangular and sides relatively straight (1). In state 1, the area above the orbits is expanded and an isosceles triangle is formed by the rectilinear sides. In certain taxa, a slight concavity is seen above the orbits, but anterior and posterior to this, there is no indication of a sinusoidal or recurved edge.
8. Frontal width: element broad and short (0); intermediate dimensions (1); or long and narrow (2). Mosasauroid frontals can be separated into a group that generally has a maximum length to maximum width ratio greater than 2:1 (state 2), between 1.5:1 and 2:1 (state 1), or equal to or less than 1.5:1 (state 0).
9. Frontal narial emargination: frontal not invaded by posterior end of nares (0); or distinct embayment present (1). In some mosasauroids, the posterior ends of the nares are concomitant with the anterior terminus of the frontal-prefrontal suture and, therefore, there is no marginal invasion of the frontal by the opening. However, in other mosasauroids this suture begins anterior and lateral to the posterior ends of the nares, causing a short emargination into the frontal.
10. Frontal midline dorsal keel: absent (0); or low, fairly inconspicuous (1); or high, thin, and well-developed (2).
11. Frontal ala shape: sharply acuminate (0); or more broadly pointed or rounded (1). In state 0, the anterolateral edge of the ala is smoothly concave, thus helping to form the sharply pointed or rounded and laterally oriented posterior corners. In some forms the anterolateral edge of the ala may be concave, but the tip is not sharp and directed laterally.
12. Frontal olfactory canal embrasure: canal not embraced ventrally by descending processes (0); or canal almost or completely enclosed below (1). In state 1, very short descending processes from the sides of the olfactory canal surround and almost, or totally, enclose the olfactory nerve.
13. Frontal posteroventral midline: tabular boss immediately anterior to the frontal-parietal suture absent (0); or present (1). A triangular boss with a flattened ventral surface at the posterior end of the olfactory canal is represented by state 1.
14. Frontal-parietal suture overlap orientation: apposing surfaces with no overlap (0); suture with oblique median frontal and parietal ridges contributing to overlap (1); or with all three ridges almost horizontal (2). In state 0, the median ridge from the frontal and the single parietal ridge are oriented at a distinct angle to the upper skull surface while the outer, or lateral, frontal ridge appears to be nearly horizontal. In *Tylosaurus nepaeolicus* and T. *proriger* (state 1), the obliquity of the intercalating ridges is reclined almost to the horizontal, greatly extending the amount of lateral overlap.
15. Frontal invasion of parietal I: lateral sutural flange of frontal posteriorly extended (0); or median frontal sutural flange posteriorly extended (1); or both extended (2); or suture straight (3). In all mosasaurines the oblique median frontal sutural ridge extends onto the dorsal surface of the parietal table and embraces a portion of the anterior table within a tightly crescentic midline embayment. In *Plioplatecarpus* and *Platecarpus*, the lateral oblique sutural ridge from the frontal is greatly protracted posteriorly to cause a large, anteriorly convex embayment in the dorsal frontal-parietal suture. In this case the entire posterolateral corner of the frontal is extended backwards to embrace the anterolateral portion of the parietal table on both sides. Consequently, the parietal foramen is very widely embraced laterally and the oblique anterior sutural ridge of the parietal occupies a position inside the embayment within the frontal. *Dallasaurus* was recoded as ?.
16. Frontal medial invasion of parietal II: if present, posteriorly extended median sutural flange short (0); or long (1). The median oblique sutural flange is either short, not reaching back to the parietal foramen (state 0), or tightly embraces the foramen while extending backwards to a position even with or beyond its posterior edge (state 1).
17. Parietal length: dorsal surface relatively short with epaxial musculature insertion posterior, between suspensorial rami only (0); or dorsal surface elongate, with epaxial musculature insertion dorsal as well as posterior (1).
18. Parietal table shape: generally rectangular to trapezoidal, with sides converging, but not meeting (0); or triangular, with sides contacting in front of suspensorial rami (1); or triangular table with posterior portion forming parasagittal crest or ridge (2).
19. Parietal foramen size: relatively small (0); or large (1). If the foramen is smaller than or equal to the area of the stapedial pit, it is considered small. If the foramen is significantly larger or if the distance across the foramen is more than half the distance between it and the nearest edge of the parietal table, the derived state is achieved.
20. Parietal foramen position I: foramen generally nearer to center of parietal table, well away from frontal-parietal suture (0); or close to or barely touching suture (1); or huge foramen straddling suture and deeply invading frontal (2). Generally in state 1, the distance from the foramen to the suture is about equal to or less than one foramen’s length.
21. Parietal foramen ventral opening: opening is level with main ventral surface (0); or opening surrounded by a rounded, elongate ridge (1).
22. Parietal posterior shelf: presence of a distinct horizontal shelf projecting posteriorly from between the suspensorial rami (0); or shelf absent (1). In some mosasauroids, a somewhat crescent-shaped shelf (in dorsal view) lies at the posterior end of the bone medial to, and below, the origination of the suspensorial rami.
23. Parietal suspensorial ramus compression: greatest width vertical or oblique (0); or greatest width horizontal (1). In *Tylosaurus*, the anterior edge of the ramus begins very low on the lateral wall of the descending process, leading to formation of a proximoventral sulcus, but the straps are horizontal distally.
24. Parietal union with supratemoral: suspensorial ramus from parietal overlaps supratemporal without interdigitation (0); or forked distal ramus sandwiches proximal end of supratemporal (1).
25. Prefrontal supraorbital process: process absent, or present as a very small rounded knob (0); or a distinct, to large, triangular, or rounded overhanging wing (1).
26. Prefrontal-postorbitofrontal contact: absent (0); prefrontal overlapped ventrally by postorbitofrontal (0); or prefrontal overlapped laterally by postorbitofrontal (1). Postorbitofrontal ventral overlap of the prefrontal is extreme in Platecarpus tympaniticus and Plioplatecarpus, such that there is even a thin flange of the frontal interjected between the prefrontal above and the postorbitofrontal below. In *T. proriger*, the postorbitofrontal sends a long narrow process forward to fit into a lateral groove on the prefrontal. In *Plesiotylosaurus*, the overlap is relatively short and more oblique, and there is no groove on the prefrontal.
27. Postorbitofrontal shape: narrow (0); or wide (1). In *Clidastes* and the *Globidensini*, the lateral extent of the element is almost equal to half of the width of the frontal and the outline of the bone is basically squared.
28. Postorbitofrontal transverse dorsal ridge: absent (0); or present (1). In state 1, an inconspicuous, low, and narrowly rounded ridge traces from the anterolateral corner of the parietal suture across the top of the element to disappear behind the origin of the jugal process.
29. (modified) Maxilla tooth number: 20–24 (0); or 17–19 (1); or 15–16 (2); 12–14 (3).
30. Maxillo-premaxillary suture posterior terminus: suture ends above a point that is anterior to or level with the midline of the fourth maxillary tooth (0); or between the fourth and ninth teeth (1); or level with or posterior to the ninth tooth (2). These somewhat arbitrary divisions of the character states are meant to describe in more concrete terms those sutures that terminate far anteriorly, those that terminate less anteriorly, and those that terminate near the midlength of the maxilla, respectively.
31. Maxilla posterodorsal extent: process absent (0); recurved wing of maxilla prevents emargination of prefrontal on dorsolateral edge of external naris (1); or does not (2).
32. Jugal posteroventral angle: angle very obtuse or curvilinear (0); or slightly obtuse, near 120^o^ (1); or 90^o^ (2). *Aigialosaurus* was recoded as having state 1, *Russellosaurus* and *Tethysaurus* were recoded as having state 2.
33. Jugal posteroventral process: absent (0); or present (1).
34. Ectopterygoid contact with maxilla: present (0); or absent (1).
35. Pterygoid tooth row elevation: teeth arise from robust, transversely flattened, main shaft of pterygoid (0); or teeth arise from thin pronounced vertical ridge (1). In state 0, the teeth emanate from the relatively planar surface of the thick, slightly dorsoventrally compressed main shaft of the pterygoid. In state 1, a tall, thin dentigerous ridge emanates ventrally from a horizontal flange that forms the base of the quadratic ramus and the ectopterygoid process, thus causing the main shaft to be trough-shaped. Although the outgroup we selected (*Varanus*) does not possess pterygoid teeth we decided to code the primitive condition as state 0 because that is the condition observed in fossil varanoids like *Ovoo gurval* and basal anguimorphs like *Ophisaurus apodus*.
36. Pterygoid tooth size: anterior teeth significantly smaller than marginal teeth (0); or anterior teeth large, approaching size of marginal teeth (1). As per the argument discussed for character 40 we coded the outgroup as having state 0.
37. Quadrate suprastapedial process length: process short, ends at a level well above midheight (0); or of moderate length, ending very near midheight (1); or long, distinctly below midheight (2); suprastapedial process absent (3). *Russellosaurus* was recoded as having state 2.
38. Quadrate suprastapedial process constriction: distinct dorsal constriction (0); or virtually no dorsal constriction (1). Lack of constriction results in an essentially parallel-sided process in posterodorsal view, but can also include the tapering form characteristic of some *Tylosaurus*.
39. Quadrate suprastapedial ridge: if present, ridge on ventromedial edge of suprastapedial process indistinct, straight and/or narrow (0); or ridge wide, broadly rounded, and curving downward, especially above stapedial pit (1).
40. Quadrate suprastapedial process fusion: no fusion present (0); or process fused to, or in extensive contact with, elaborated process from below (1). A posterior rugose area may be inflated and broadened mediolaterally to partially enclose the ventral end of a broad and elongate suprastapedial process as in *Halisaurus*. In *Globidens*, *Prognathodon*, and *Plesiotylosaurus*, the process is fused ventrally to a narrow pedunculate medial extension of the tympanic rim. A similar condition is present in *Ectenosaurus*, except that the tympanic rim is not medially extended and has a short projection that overlaps a portion of the suprastapedial process posteriorly.
41. Quadrate stapedial pit shape: pit broadly oval to almost circular (0); or relatively narrowly oval (1); or extremely elongate with a constricted middle (2). In state 0, the length to width ratio is less than 1.8:1; in state 1 it ranges from 1.8:1 to 2.4:1; and in state 2, it is greater than 2.4:1.
42. Quadrate posteroventral ascending tympanic rim condition: ascending ridge small or absent (0); or a high, elongate triangular crest (1); or a crest extremely produced laterally (2). In state 1, this extended rim causes a fairly deep sulcus in the ventral portion of the intratympanic cavity. In *Plioplatecarpus*, the entire lower tympanic rim and ala are expanded into a large conch (state 2), which tremendously increases the depth of the intratympanic cavity.
43. Quadrate ala thickness: ala thin (0); or thick (1). In state 0, the bone in the central area of the ala is only about 1 mm thick in medium-sized specimens and that area is usually badly crushed or completely destroyed. Alternatively, the ala extends from the main shaft with only minor thinning, providing a great deal of strength to the entire bone. *Tethysaurus* was recoded as having state 0.
44. Quadrate conch: ala and main shaft encompassing a deeply bowled area (0); or alar concavity shallow (1). A relatively deeper sulcus in the anterior part of the intratympanic cavity and more definition to the ala and the main shaft are features of state 0. *Tethysaurus* was recoded as having state 0.
45. Basisphenoid pterygoid process shape: process relatively narrow with articular surface facing mostly anterolaterally (0); or somewhat thinner, more fan- shaped with a posterior extension of the articular surface causing a more lateral orientation (1).
46. Quadrate ala groove: absent (0); or long, distinct, and deep groove present in anterolateral edge of ala (1); or groove along dorsal margin of quadrate ala (2).
47. Quadrate median ridge: single thin, high ridge, dorsal to ventral (0); or ridge low and rounded with divergent ventral ridges (1).
48. Quadrate anterior ventral condyle modification: no upward deflection of anterior edge of condyle (0); or distinct deflection present (1). A relatively narrow bump in the otherwise horizontal trace of the anterior articular edge is also supertended by a sulcus on the anteroventral face of the bone.
49. Quadrate ventral condyle: condyle saddle-shaped, concave in anteroposterior view (0); or gently domed, convex in any view (1).
50. Basioccipital tubera size: short (0); or long (1). Long tubera are typically parallel-sided in posterior profile and protrude ventrolaterally at exactly 45^o^ from horizontal. Short tubera have relatively large bases that taper distally, and emanate more horizontally.
51. Basioccipital tubera shape: tubera not anteroposteriorly elongate (0); or anteroposteriorly elongate with rugose ventrolateral surfaces (1).
52. Basioccipital canal: absent (0); or present as a pair separated by a median septum (1); or present as a single bilobate canal (2).
53. Dentary tooth number: 20–24 (0); 17–19 (1); 15–16 (2); 14 (3); 13 (4); 12 (5). It is easy to assume this character is correlated with the number of maxillary teeth, except that is not the case in *Ectenosaurus* *clidastoides*, which has 16 or 17 maxillary teeth and only 13 dentary teeth.
54. Dentary anterior projection: absent (0); short (1); or long (2). In state 1, the projection of bone anterior to the first tooth is at least the length of a complete tooth space. *Russellosaurus* was recoded as not applicable.
55. Dentary medial parapet: parapet positioned at base of tooth roots (0); or elevated and strap-like, enclosing about half of height of tooth attachment in shallow channel (1), or strap equal in height to lateral wall of bone (2). States 1 and 2 are possible sequential stages of modification from a classically pleurodont dentition to the typical mosasaur ‘sub-thecodont’ dentition. *Tethysaurus* was recoded as ?.
56. Splenial-angular articulation shape: splenial articulation in posterior view almost circular (0); or laterally compressed (1).
57. Splenial-angular articular surface: essentially smooth concavoconvex surfaces (0); or distinct horizontal tongues and grooves present (1).
58. Coronoid shape: coronoid with slight dorsal curvature, posterior wing not widely fan-shaped (0); or very concave above, posterior wing greatly expanded (1). *Ectenosaurus* was recoded as having state 0.
59. Coronoid posteromedial process: small but present (0); or absent (1). *Russellosaurus* was recoded as having state 0, *Ectenosaurus* was recoded as having state 1.
60. Coronoid medial wing: does not reach angular (0); or contacts angular (1). *Aigialosaurus* was recoded as ?.
61. Coronoid posterior wing: without medial crescentic pit (0); or with distinct excavation (1). In state 1, there is a posteriorly open, ‘C’-shaped excavation in the medial side of the posterior wing of this element. *Dallasaurus* was recoded as ?.
62. Surangular coronoid buttress: low, thick, about parallel to lower edge of mandible (0); or high, thin, rapidly rising anteriorly (1). A rounded dorsal edge of the surangular remains almost parallel to the ventral edge as it approaches the posterior end of the coronoid, meeting the latter element near its posteroventral edge in state 0. In state 1, the dorsal edge rises and thins anteriorly until meeting the posterior edge of the coronoid near its apex, producing a triangular posterior mandible in lateral aspect.
63. Surangular-articular suture position: behind the condyle in lateral view (0); or at middle of glenoid on lateral edge (1); anterior to condyle (2). In state 1, there is usually an interdigitation in the dorsal part of the suture. *Aigialosaurus dalmaticus* was rescored as 2.
64. Surangular-articular lateral suture trace: suture descends and angles or curves anteriorly (0); or is virtually stra ight throughout its length (1). In state 1, the suture trails from the glenoid posteriorly about halfway along the dorsolateral margin of the retroarticular process, then abruptly turns anteriorly off the edge and strikes in a straight line for the posterior end of the angular.
65. Articular retroarticular process inflection: moderate inflection, less than 60^o^ (0); or extreme inflection, almost 90^o^ (1).
66. Articular retroarticular process innervation foramina: no large foramina on lateral face of retroarticular process (0); or one to three large foramina present (1).
67. Tooth surface I: teeth finely striate medially (0); or not medially striate (1). In “Russellosaurinae,” medial tooth striations are very fine and groups of tightly spaced striae are usually set apart by facets, leading to a fasciculate appearance. *Angolasaurus* was recoded as ?, *Aigialosaurus* was recoded as having state 1.
68. Tooth surface II: teeth not coarsely textured (0); or very coarsely ornamented with bumps and ridges (1). In both species of *Globidens* and in *Prognathodon* *overtoni*, the coarse surface texture is extreme, consisting of thick pustules, and vermiform or anastomosing ridges. Teeth in *P.* *rapax* are smooth over the majority of their surface, but usually a few widely scattered, large, very long, sharp-crested vermiform ridges are present.
69. Tooth facets: absent (0); or present (1). *Halisaurus* teeth are smoothly rounded except for the inconspicuous carinae. *Clidastes* is described in numerous places as having smooth unfaceted teeth, but many immature individuals and some larger specimens have teeth with three distinct facets on the medial faces. Adult *Tylosaurus* proriger has indistinct facets. *Mosasaurus* has taken this characteristic to the extreme. *Russellosaurus*, *Tethysaurus*, *Angolasaurus*, *Ectenosaurus*, *Platecarpus* (*P. planifrons* and *P. tympaniticus*), and *Plioplatecarpus* have been recoded as having state 0.
70. Tooth fluting: absent (0); or present (1). In *Ectenosaurus*, and some *Platecarpus* *planifrons*, several broadly rounded vertical ridges alternate with shallow, round-bottomed grooves completely around the teeth. *Tethysaurus* was recoded as having both states 0 and 1, because grooves can be observed in larger specimens. *Angolasaurus* was recoded as having state 1.
71. Tooth inflation: crowns of posterior marginal teeth conical, tapering throughout (0); or crowns of posterior marginal teeth swollen near the tip or above the base (1).The rear teeth of *Globidens* and *Prognathodon overtoni* are distinctly fatter than other mosasauroid teeth, but those of *P. rapax* are also swollen immediately distal to the base.
72. Tooth carinae I: absent (0); or present but extremely weak (1); or strong and elevated (2). *Halisaurus* exhibits the minimal expression of this character (state 1) in that its marginal teeth are almost perfectly round in cross-section; the carinae are extremely thin and barely stand above the surface of the teeth.
73. Tooth carinae serration: absent (0); or present (1).
74. Tooth replacement mode: replacement teeth form in shallow excavations (0); or in subdental crypts (1). All mosasauroids that can be evaluated have an ‘anguimorph’ type of tooth replacement, which is to have interdental positioning of replacement teeth and resorption pits associated with each. *Angolasaurus* was recoded as ?.
75. Atlas neural arch: notch in anterior border (0); or no notch in anterior border (1). *Dallasaurus* was recoded as ?.
76. Atlas synapophysis: extremely reduced (0); or large and elongate (1). In state 1, a robust synapophysis extends well posteroventral to the medial articular surface for the atlas centrum, and it may be pedunculate (*Clidastes*) or with a ventral ‘skirt’ that gives it a triangular shape (*Mosasaurus*). A very small triangular synapophysis barely, if at all, extends posterior to the medial articular edge in state 0.
77. Zygosphenes and zygantra: absent (0); or present (1). This character assesses only the presence of zygosphenes and zygantra, not their relative development. Nonfunctional and functional are considered as present. Although the outgroup we selected (*Varanus*) does not possess zygosphene and zygantra we decided to code the primitive condition as present because these structures can be observed in primitive varanoids like *Saniwa*.
78. Zygosphene and zygantra number: present on many vertebrae (0); or present on only a few (1). As per the argument discussed for character 84 we coded the outgroup as having state 0.
79. Hypapophyses: last hypapophysis occurs on or anterior to seventh vertebra (0); or on eight or posteriorly (1).
80. Synapophysis height: facets for rib articulations tall and narrow on posterior cervicals and anterior trunk vertebrae (0); or facets ovoid, shorter than the centrum height on those vertebrae (1).
81. Synapophysis length: synapophyses of middle trunk vertebrae not laterally elongate (0); or distinctly laterally elongate (1). The lateral extension of the synapophyses from the middle of the trunk is as much as 70–80% of the length of the same vertebra is represented by state 1.
82. Synapophysis ventral extension: synapophyses extend barely or not at all below ventral margin of cervical centra (0); or some extend far below ventral margin of centrum (1). In state 1, two or more anterior cervical vertebrae have rib articulations that dip well below the centrum, causing a very deeply concave ventral margin in anterior profile.
83. Vertebral condyle inclination: condyles of trunk vertebrae inclined (0); or condyles vertical (1).
84. Vertebral condyle shape I: condyles of anterior-most trunk vertebrae extremely dorsoventrally depressed (0); or essentially equidimensional (1). In state 0, posterior height: width ratios of anterior trunk vertebrae are close to 2:1. In state 1, they are between to 4:3 and 1:1.
85. Vertebral condyle shape II: condyles of posterior trunk vertebrae not higher than wide (0); or slightly compressed (1). In state 1, the posterior condylar aspect reveals outlines that appear to be higher than wide and even perhaps slightly subrectangular, due to the slight emargination for the dorsal nerve cord.
86. Vertebral synapophysis dorsal ridge: sharp ridge absent on posterior trunk synapophyses (0); or with a sharp-edged and anteriorly precipitous ridge connecting distal synapophysis with prezygapophysis (1). In state 0, the ridge in question, if present, may be incomplete or it may be rounded across the crest with the anterior and posterior sides about equally sloping.
87. Vertebral length proportions: cervical vertebrae distinctly shorter than longest vertebrae (0); or almost equal or are the longest (1).
88. Presacral vertebrae number II: if few, then 28 or 29 (0); 30 or 31 (1); 39 or more (2). Here, presacral vertebrae are considered to be all those anterior to the first bearing an elongate transverse process.
89. Sacral vertebrae number: two (0); or less than two (1). Numerous well preserved specimens of derived mosasauroids have failed to show any direct contact of the pelvic girdle with vertebrae in the sacral area. Certainly, no transverse processes bear any type of concave facet for the ilium, and so it is generally assumed that a ligamentous contact was established with only one transverse process. Depending on one’s perspective, it could be said that derived mosasauroids have either no or one sacral vertebra.
90. Caudal dorsal expansion: neural spines of tail all uniformly shortened posteriorly (0); or several spines dorsally elongated behind middle of tail (1). *Dallasaurus* was recoded as ?.
91. Haemal arch length: haemal arches about equal in length to neural arch of same vertebra (0); or length about 1.5 times greater than neural arch length (1). This ratio may be as great as 1.2:1 in state 0. Comparison is most accurate in the middle of the tail and is consistent even on those vertebrae in which the neural spines are also elongated.
92. Haemal arch articulation: arches articulating (0); or arches fused to centra (1).
93. Tail curvature: no structural downturn of tail (0); or tail with curved posterior portion (1).
94. Body proportions: head and trunk shorter than or about equal to tail length (0); or head and trunk longer than tail (1).
95. Scapula/coracoid size: both bones about equal (0); or scapula about half the size of coracoid (1). *Dallasaurus* was recoded as ?.
96. Scapula width: no anteroposterior widening (0); or distinct fan-shaped widening (1); or extreme widening (2). In state 0, the anterior and posterior edges of the scapula encompass less than one quarter of the arc of a circle, but in state 1, the arc is increased to approximately one third. In state 2, the distal margin encompasses almost a half-circle and the anterior and posterior borders are of almost equal length.
97. Scapula dorsal convexity: if scapula widened, dorsal margin very convex (0); or broadly convex (1). In state 0, the anteroposterior dimension is almost the same as the proximodistal dimension. In state 1, the anteroposterior dimension is much larger.
98. Scapula posterior emargination: posterior border of bone gently concave (0); or deeply concave (1). In state 1, there is a deeply arcuate emargination on the posterior scapular border, just dorsal to the glenoid. It is immediately bounded dorsally by a corner, which begins a straight-edged segment that continues to the dorsal margin.
99. Scapula-coracoid suture: unfused scapula-coracoid contact has interdigitate suture anteriorly (0); or apposing surfaces without interdigitation (1). *Dallasaurus* was recoded as ?.
100. Coracoid neck elongation: neck rapidly tapering from medial corners to a relatively broad base (0); or neck gradually tapering to a relatively narrow base (1); coracoid neck absent (2). In state 1, this character describes an outline of the bone, which is nearly symmetrical and gracefully fan-shaped, with gently concave, nearly equidistant sides.
101. Coracoid anterior emargination: present (0); or absent (1).
102. Humerus length: humerus distinctly elongate, about three or more times longer than distal width (0); or greatly shortened, about 1.5 to 2 times longer than distal width (1); or length and distal width virtually equal (2); or distal width slightly greater than length (3).
103. Humerus postglenoid process: absent or very small (0); or distinctly enlarged (1).
104. Humerus glenoid condyle: if present, condyle gently domed and elongate, ovoid in proximal view (0); or condyle saddle-shaped, subtriangular in proximal view and depressed (1); or condyle highly domed or protuberant and short ovoid to almost round in proximal view (2). In some taxa, the condylar surfaces of the limbs were finished in thick cartilage and there was no bony surface of the condyle to be preserved. This condition is scored as not represented. In some taxa, the glenoid condyle extends more proximally than does the postglenoid process (state 2), and it is not ovoid as state 0. *Dallasaurus* was recoded as having state 0.
105. Humerus deltopectoral crest: crest undivided (0); or split into two separate insertional areas (1). In state 1, the deltoid crest occupies an anterolateral or anterior position confluent with the glenoid condyle, while the pectoral crest occupies a medial or anteromedial area that may or may not be confluent with the glenoid condyle. The deltoid crest is often quite short, broad, and indistinct, being easily erased by degradational taphonomic processes.
106. Humerus pectoral crest: located anteriorly (0); or medially (1). In state 1, the pectoral crest is located near the middle of the flexor (or medial) side on the proximal end of the bone.
107. Humerus ectepicondylar groove: groove or foramen present on distolateral edge (0); or absent (1).
108. Humerus ectepicondyle: absent (0); or present as a prominence (1). A radial tuberosity is reduced in size in *Prognathodon*, but very elongated in *Plesiotylosaurus*. *Tethysaurus* was recoded as having state 0.
109. Humerus entepicondyle: absent (0); or present as a prominence (1). The ulnar tuberosity protrudes posteriorly and medially from the posterodistal corner of the bone immediately proximal to the ulnar facet, causing a substantial dilation of the posterodistal corner of the humerus. *Tethysaurus* was recoded as having state 0.
110. Radius shape: radius not expanded anterodistally (0); or slightly expanded (1); or broadly expanded (2).
111. Ulna contact with centrale: broad ulnare prevents contact (0); or ulna contacts centrale (1). In state 1, the ulnare is omitted from the border of the antebrachial foramen. There is usually a well-developed faceted articulation between the ulna and the centrale (or intermedium, as used by Russell, 1967).
112. Radiale size: large and broad (0); or small to absent (1).
113. Carpal reduction: carpals number six or more (0); or five or less (1).
114. Pisiform: present (0); or absent (1).
115. Metacarpal I expansion: spindle-shaped, elongate (0); or broadly expanded (1). The broad expansion is also associated with an anteroproximal overhanging crest in every case observed.
116. Phalanx shape: phalanges elongate, spindle-shaped (0); or blocky, hourglass-shaped (1). *Mosasaurus* and *Plotosaurus* have phalanges that are slightly compressed and anteroposteriorly expanded on both ends. *Dallasaurus* was recoded as ?.
117. Ilium crest: crest blade-like, articulates with sacral ribs (0); or elongate, cylindrical, does not articulate with sacral ribs (1).
118. Ilium acetabular area: arcuate ridge supertending acetabulum (0); or acetabulum set into broad, short ‘V’-shaped notch (1). The primitive ilium has the acetabulum impressed on the lateral wall of the bone, with a long narrow crest anterodorsally as the only surrounding topographic feature. In state 1, the acetabular area is set into a short, broadly ‘V’-shaped depression that tapers dorsally. The lateral walls of the ilium are therefore distinctly higher than the rim of the acetabulum.
119. Pubic tubercle condition: tubercle an elongate protuberance located closer to the midlength of the shaft (0); or a thin semicircular crest-like blade located close to the acetabulum (1).
120. Ischiadic tubercle size: elongate (0); or short (1). In state 0, the tubercle is as long as the shaft of the ischium is wide, but it is only a short narrow spur in state 1.
121. Astragalus: notched emargination for the crural foramen, without pedunculate fibular articulation (0); or without notch, pedunculate fibular articulation present (1). For state 0, the tibia and fibula are of equal length about the crural foramen and the astragalus contacts both to about the same degree. The form of the latter element is symmetrical and subcircular with a sharp proximal notch. In state 1, the outline of the element is basically reniform and the tibial articulation is on the same line as the crural emargination. The fibula is also shortened and its contact with the astragalus is narrow.
122. Appendicular epiphyses: formed from ossified cartilage (0); or from thick unossified cartilage (1); or epiphyses missing or extremely thin (2). Ends of the limb bones show distinct vascularization and rugose surfaces indicating an apparently thick non-vascularized, unossified cartilage cap. Extremely smooth articular surfaces suggest the epiphyses were excessively thin or perhaps even lost.
123. Hyperphalangy: absent (0); or present (1). Hyperphalangy is defined as presence of one or more extra phalanges as compared to the primitive amniote formula of 2-3-4-5-3.
124. Posterior thoracic vertebra: not markedly longer than anterior thoracic vertebrae (0); or are markedly longer (1).
125. Ectopterygoid process of pterygoid: distal portion of process not offset anterolaterally and/or lacking longitudinal grooves and ridges (0); distal portion of process is offset anterolaterally and bears longitudinal grooves and ridges (1).

**Additional literature cited:**

LeBlanc, A. R. H., M. W. Caldwell, and N. Bardet. 2012. A new mosasaurine from the Maastrichtian (Upper Cretaceous) phosphates of Morocco and the implications for the systematics of the Mosasaurinae. Journal of Vertebrate Paleontology 32:82–104.

Palci, A., Caldwell, M. W., and Papazzoni, C. A. 2013. A new genus and subfamily of mosasaurs from the Upper Cretaceous of northern Italy. Journal of Vertebrate Paleontology, 33(3):599–612.

Russell, D. A. 1967. Systematics and morphology of American mosasaurs. Bulletin of the Peabody Museum of Natural History 23:1–237.

**Character list applying contingent character coding**

New characters states included herein: Character 41 state 3; character 68 state 2; character 106 state 2.

(1) Premaxilla predental rostrum I: total lack of a bony rostrum (0); or presence of any predental rostrum (1). In lateral profile, the anterior end of the premaxilla either exhibits some bony anterior projection above the dental margin, or the bone recedes posterodorsally from the dental margin. State 1 produces a relatively taller lateral profile with an obvious ‘bow’ or ‘prow.’

(2) Premaxilla predental rostrum II: rostrum very short and obtuse (0); or distinctly protruding (1); or very large and inflated (2). In *Clidastes* a short, acute, protruding rostrum (state 1) produces a ‘V’-shaped dorsal profile and, as far as is known, is peculiar to that genus. An alternative condition, described as ‘U’- shaped, includes those taxa whose rostral conditions span the whole range of states of characters 1 and 2. Hence, the descriptive character is abandoned in favor of a more informative structure-based series.

(3) Premaxilla shape: bone broadly arcuate anteriorly (0); or relatively narrowly arcuate or acute anteriorly (1). In virtually all lizards the premaxilla is a very widely arcuate and lightly constructed element, and the base of the internarial process is quite narrow as in Aigialosaurus bucchichi. All other mosasaurids have a very narrowed premaxilla with the teeth forming a tight curve and the internarial process being proportionally wider (state 1). *Tethysaurus* was recoded as having state 0.

(4) Premaxilla internarial bar width: narrow, distinctly less than half of the maximum width of the rostrum in dorsal view (0); or wide, being barely narrower than the rostrum (1). *Aigialosaurus* was recoded as having state 0.

(5) Premaxilla internarial bar base shape: triangular (0); or rectangular (1). A vertical cross-section through the junction of the internarial bar and the dentigerous rostrum produces an inverted triangle in most taxa. But in state 1, this cross-section is transversely rectangular because the broad ventral surface of the bar is planar.

(6) Premaxilla internarial bar dorsal keel: absent (0); or present (1). In state 1 a ridge rises above the level of a normally smoothly continuous transverse arch formed by the bones of the anterior muzzle.

(7) Premaxilla internarial bar venter: with entrance for the fifth cranial nerve close to rostrum (0); or far removed from rostrum (1). The conduit that marks the path of the fifth cranial nerve from the maxilla into the premaxilla is expressed as a ventrolateral foramen within the premaxillo-maxillary sutural surface at the junction of the internarial bar and the dentigerous rostrum. State 1 includes a long shallow groove on the ventral surface of the bar. Anteriorly, this groove becomes a tunnel entering the bone at an extremely shallow angle, but disappearing below the surface at least 1 cm behind the rostrum.

(8) Frontal shape in front of the orbits: sides sinusoidal (0); or bone nearly triangular and sides relatively straight (1). In state 1, the area above the orbits is expanded and an isosceles triangle is formed by the rectilinear sides. In certain taxa, a slight concavity is seen above the orbits, but anterior and posterior to this, there is no indication of a sinusoidal or recurved edge.

(9) Frontal width: element broad and short (0); intermediate dimensions (1); or long and narrow (2). Mosasauroid frontals can be separated into a group that generally has a maximum length to maximum width ratio greater than 2:1 (state 2), between 1.5:1 and 2:1 (state 1), or equal to or less than 1.5:1 (state 0).

(10) Frontal narial emargination: frontal not invaded by posterior end of nares (0); or distinct embayment present (1). In some mosasauroids, the posterior ends of the nares are concomitant with the anterior terminus of the frontal-prefrontal suture and, therefore, there is no marginal invasion of the frontal by the opening. However, in other mosasauroids this suture begins anterior and lateral to the posterior ends of the nares, causing a short emargination into the frontal.

(11) Frontal midline dorsal keel: absent (0); or low, fairly inconspicuous (1); or high, thin, and well-developed (2).

(12) Frontal ala shape: sharply acuminate (0); or more broadly pointed or rounded (1). In state 0, the anterolateral edge of the ala is smoothly concave, thus helping to form the sharply pointed or rounded and laterally oriented posterior corners. In some forms the anterolateral edge of the ala may be concave, but the tip is not sharp and directed laterally.

(13) Frontal olfactory canal embrasure: canal not embraced ventrally by descending processes (0); or canal almost or completely enclosed below (1). In state 1, very short descending processes from the sides of the olfactory canal surround and almost, or totally, enclose the olfactory nerve.

(14) Frontal posteroventral midline: tabular boss immediately anterior to the frontal-parietal suture absent (0); or present (1). A triangular boss with a flattened ventral surface at the posterior end of the olfactory canal is represented by state 1.

(15) Frontal-parietal suture: apposing surfaces with low interlocking ridges (0); or with overlapping flanges (1). In state 0, an oblique ridge on the anterior sutural surface of the parietal intercalates between a single median posterior and a single lateral posterior ridge from the frontal. In state 1, these ridges are protracted into strongly overlapping flanges. The dorsal trace of the suture can be quite complex with a portion of the parietal embraced by the posterior extension of these frontal flanges.

(16) Frontal-parietal suture overlap orientation: suture with oblique median frontal and parietal ridges contributing to overlap (0); or with all three ridges almost horizontal (1). In state 0, the median ridge from the frontal and the single parietal ridge are oriented at a distinct angle to the upper skull surface while the outer, or lateral, frontal ridge appears to be nearly horizontal. In *Tylosaurus nepaeolicus* and T. *proriger* (state 1), the obliquity of the intercalating ridges is reclined almost to the horizontal, greatly extending the amount of lateral overlap.

(17) Frontal invasion of parietal I: lateral sutural flange of frontal posteriorly extended (0); or median frontal sutural flange posteriorly extended (1); or both extended (2); or suture straight (3). In all mosasaurines the oblique median frontal sutural ridge extends onto the dorsal surface of the parietal table and embraces a portion of the anterior table within a tightly crescentic midline embayment. In *Plioplatecarpus* and *Platecarpus*, the lateral oblique sutural ridge from the frontal is greatly protracted posteriorly to cause a large, anteriorly convex embayment in the dorsal frontal-parietal suture. In this case the entire posterolateral corner of the frontal is extended backwards to embrace the anterolateral portion of the parietal table on both sides. Consequently, the parietal foramen is very widely embraced laterally and the oblique anterior sutural ridge of the parietal occupies a position inside the embayment within the frontal. *Dallasaurus* was recoded as ?.

(18) Frontal medial invasion of parietal II: if present, posteriorly extended median sutural flange short (0); or long (1). The median oblique sutural flange is either short, not reaching back to the parietal foramen (state 0), or tightly embraces the foramen while extending backwards to a position even with or beyond its posterior edge (state 1).

(19) Parietal length: dorsal surface relatively short with epaxial musculature insertion posterior, between suspensorial rami only (0); or dorsal surface elongate, with epaxial musculature insertion dorsal as well as posterior (1).

(20) Parietal table shape: generally rectangular to trapezoidal, with sides converging, but not meeting (0); or triangular, with sides contacting in front of suspensorial rami (1); or triangular table with posterior portion forming parasagittal crest or ridge (2).

(21) Parietal foramen size: relatively small (0); or large (1). If the foramen is smaller than or equal to the area of the stapedial pit, it is considered small. If the foramen is significantly larger or if the distance across the foramen is more than half the distance between it and the nearest edge of the parietal table, the derived state is achieved.

(22) Parietal foramen position I: foramen generally nearer to center of parietal table, well away from frontal-parietal suture (0); or close to or barely touching suture (1); or huge foramen straddling suture and deeply invading frontal (2). Generally in state 1, the distance from the foramen to the suture is about equal to or less than one foramen’s length.

(23) Parietal foramen ventral opening: opening is level with main ventral surface (0); or opening surrounded by a rounded, elongate ridge (1).

(24) Parietal posterior shelf: presence of a distinct horizontal shelf projecting posteriorly from between the suspensorial rami (0); or shelf absent (1). In some mosasauroids, a somewhat crescent-shaped shelf (in dorsal view) lies at the posterior end of the bone medial to, and below, the origination of the suspensorial rami.

(25) Parietal suspensorial ramus compression: greatest width vertical or oblique (0); or greatest width horizontal (1). In *Tylosaurus*, the anterior edge of the ramus begins very low on the lateral wall of the descending process, leading to formation of a proximoventral sulcus, but the straps are horizontal distally.

(26) Parietal union with supratemoral: suspensorial ramus from parietal overlaps supratemporal without interdigitation (0); or forked distal ramus sandwiches proximal end of supratemporal (1).

(27) Prefrontal supraorbital process: process absent, or present as a very small rounded knob (0); or a distinct, to large, triangular, or rounded overhanging wing (1).

(28) Prefrontal contact with postorbitofrontal: no contact at edge of frontal (0); of elements in contact there (1). State 1 is usually described as the frontal being emarginated above the orbits. Often this character can be evaluated by examining the ventral surface of the frontal where depressions outline the limits of the sutures for the two ventral elements.

(29) Prefrontal-postorbitofrontal overlap: prefrontal overlapped ventrally by postorbitofrontal (0); or prefrontal overlapped laterally (1). Postorbitofrontal ventral overlap of the prefrontal is extreme in Platecarpus tympaniticus and

Plioplatecarpus, such that there is even a thin flange of the frontal interjected between the prefrontal above and the postorbitofrontal below. In *T. proriger*, the postorbitofrontal sends a long narrow process forward to fit into a lateral groove on the prefrontal. In *Plesiotylosaurus*, the overlap is relatively short and more oblique, and there is no groove on the prefrontal.

(30) Postorbitofrontal shape: narrow (0); or wide (1). In *Clidastes* and the *Globidensini*, the lateral extent of the element is almost equal to half of the width of the frontal and the outline of the bone is basically squared.

(31) Postorbitofrontal transverse dorsal ridge: absent (0); or present (1). In state 1, an inconspicuous, low, and narrowly rounded ridge traces from the anterolateral corner of the parietal suture across the top of the element to disappear behind the origin of the jugal process.

(32) Maxilla tooth number: 20–24 (0); or 17–19 (1); or 15–16 (2); 12–14 (3).

(33) Maxillo-premaxillary suture posterior terminus: suture ends above a point that is anterior to or level with the midline of the fourth maxillary tooth (0); or between the fourth and ninth teeth (1); or level with or posterior to the ninth tooth (2). These somewhat arbitrary divisions of the character states are meant to describe in more concrete terms those sutures that terminate far anteriorly, those that terminate less anteriorly, and those that terminate near the midlength of the maxilla, respectively.

(34) Maxilla posterodorsal process: recurved wing of maxilla dorsolaterally overlaps a portion of the anterior end of the prefrontal (0); or process absent (1).

(35) Maxilla posterodorsal extent: recurved wing of maxilla prevents emargination of prefrontal on dorsolateral edge of external naris (0); or does not (1).

(36) Jugal posteroventral angle: angle very obtuse or curvilinear (0); or slightly obtuse, near 120^o^ (1); or 90^o^ (2). *Aigialosaurus* was recoded as having state 1, *Russellosaurus* and *Tethysaurus* were recoded as having state 2.

(37) Jugal posteroventral process: absent (0); or present (1).

(38) Ectopterygoid contact with maxilla: present (0); or absent (1).

(39) Pterygoid tooth row elevation: teeth arise from robust, transversely flattened, main shaft of pterygoid (0); or teeth arise from thin pronounced vertical ridge (1). In state 0, the teeth emanate from the relatively planar surface of the thick, slightly dorsoventrally compressed main shaft of the pterygoid. In state 1, a tall, thin dentigerous ridge emanates ventrally from a horizontal flange that forms the base of the quadratic ramus and the ectopterygoid process, thus causing the main shaft to be trough-shaped. Although the outgroup we selected (*Varanus*) does not possess pterygoid teeth we decided to code the primitive condition as state 0 because that is the condition observed in fossil varanoids like *Ovoo gurval* and basal anguimorphs like *Ophisaurus apodus*.

(40) Pterygoid tooth size: anterior teeth significantly smaller than marginal teeth (0); or anterior teeth large, approaching size of marginal teeth (1). As per the argument discussed for character 40 we coded the outgroup as having state 0.

(41) Quadrate suprastapedial process length: process short, ends at a level well above midheight (0); or of moderate length, ending very near midheight (1); or long, distinctly below midheight (2); suprastapedial process absent (3). *Russellosaurus* was recoded as having state 2.

(42) Quadrate suprastapedial process constriction: distinct dorsal constriction (0); or virtually no dorsal constriction (1). Lack of constriction results in an essentially parallel-sided process in posterodorsal view, but can also include the tapering form characteristic of some *Tylosaurus*.

(43) Quadrate suprastapedial ridge: if present, ridge on ventromedial edge of suprastapedial process indistinct, straight and/or narrow (0); or ridge wide, broadly rounded, and curving downward, especially above stapedial pit (1).

(44) Quadrate suprastapedial process fusion: no fusion present (0); or process fused to, or in extensive contact with, elaborated process from below (1). A posterior rugose area may be inflated and broadened mediolaterally to partially enclose the ventral end of a broad and elongate suprastapedial process as in *Halisaurus*. In *Globidens*, *Prognathodon*, and *Plesiotylosaurus*, the process is fused ventrally to a narrow pedunculate medial extension of the tympanic rim. A similar condition is present in *Ectenosaurus*, except that the tympanic rim is not medially extended and has a short projection that overlaps a portion of the suprastapedial process posteriorly.

(45) Quadrate stapedial pit shape: pit broadly oval to almost circular (0); or relatively narrowly oval (1); or extremely elongate with a constricted middle (2). In state 0, the length to width ratio is less than 1.8:1; in state 1 it ranges from 1.8:1 to 2.4:1; and in state 2, it is greater than 2.4:1.

(46) Quadrate posteroventral ascending tympanic rim condition: ascending ridge small or absent (0); or a high, elongate triangular crest (1); or a crest extremely produced laterally (2). In state 1, this extended rim causes a fairly deep sulcus in the ventral portion of the intratympanic cavity. In *Plioplatecarpus*, the entire lower tympanic rim and ala are expanded into a large conch (state 2), which tremendously increases the depth of the intratympanic cavity.

(47) Quadrate ala thickness: ala thin (0); or thick (1). In state 0, the bone in the central area of the ala is only about 1 mm thick in medium-sized specimens and that area is usually badly crushed or completely destroyed. Alternatively, the ala extends from the main shaft with only minor thinning, providing a great deal of strength to the entire bone. *Tethysaurus* was recoded as having state 0.

(48) Quadrate conch: ala and main shaft encompassing a deeply bowled area (0); or alar concavity shallow (1). A relatively deeper sulcus in the anterior part of the intratympanic cavity and more definition to the ala and the main shaft are features of state 0. *Tethysaurus* was recoded as having state 0.

(49) Basisphenoid pterygoid process shape: process relatively narrow with articular surface facing mostly anterolaterally (0); or somewhat thinner, more fan- shaped with a posterior extension of the articular surface causing a more lateral orientation (1).

(50) Quadrate ala groove: absent (0); or long, distinct, and deep groove present in anterolateral edge of ala (1); or groove along dorsal margin of quadrate ala (2).

(51) Quadrate median ridge: single thin, high ridge, dorsal to ventral (0); or ridge low and rounded with divergent ventral ridges (1).

(52) Quadrate anterior ventral condyle modification: no upward deflection of anterior edge of condyle (0); or distinct deflection present (1). A relatively narrow bump in the otherwise horizontal trace of the anterior articular edge is also supertended by a sulcus on the anteroventral face of the bone.

(53) Quadrate ventral condyle: condyle saddle-shaped, concave in anteroposterior view (0); or gently domed, convex in any view (1).

(54) Basioccipital tubera size: short (0); or long (1). Long tubera are typically parallel-sided in posterior profile and protrude ventrolaterally at exactly 45^o^ from horizontal. Short tubera have relatively large bases that taper distally, and emanate more horizontally.

(55) Basioccipital tubera shape: tubera not anteroposteriorly elongate (0); or anteroposteriorly elongate with rugose ventrolateral surfaces (1).

(56) Basioccipital canal: absent (0); or present as a pair separated by a median septum (1); or present as a single bilobate canal (2).

(57) Dentary tooth number: 20–24 (0); 17–19 (1); 15–16 (2); 14 (3); 13 (4); 12 (5). It is easy to assume this character is correlated with the number of maxillary teeth, except that is not the case in *Ectenosaurus* *clidastoides*, which has 16 or 17 maxillary teeth and only 13 dentary teeth.

(58) Dentary anterior projection: projection of bone anterior to first tooth present (0); or absent (1).

(59) Dentary anterior projection length: short (0); or long (1). In state 1, the projection of bone anterior to the first tooth is at least the length of a complete tooth space. *Russellosaurus* was recoded as not applicable.

(60) Dentary medial parapet: parapet positioned at base of tooth roots (0); or elevated and strap-like, enclosing about half of height of tooth attachment in shallow channel (1), or strap equal in height to lateral wall of bone (2). States 1 and 2 are possible sequential stages of modification from a classically pleurodont dentition to the typical mosasaur ‘sub-thecodont’ dentition. *Tethysaurus* was recoded as ?.

(61) Splenial-angular articulation shape: splenial articulation in posterior view almost circular (0); or laterally compressed (1).

(62) Splenial-angular articular surface: essentially smooth concavoconvex surfaces (0); or distinct horizontal tongues and grooves present (1).

(63) Coronoid shape: coronoid with slight dorsal curvature, posterior wing not widely fan-shaped (0); or very concave above, posterior wing greatly expanded (1). *Ectenosaurus* was recoded as having state 0.

(64) Coronoid posteromedial process: small but present (0); or absent (1). *Russellosaurus* was recoded as having state 0, *Ectenosaurus* was recoded as having state 1.

(65) Coronoid medial wing: does not reach angular (0); or contacts angular (1). *Aigialosaurus* was recoded as ?.

(66) Coronoid posterior wing: without medial crescentic pit (0); or with distinct excavation (1). In state 1, there is a posteriorly open, ‘C’-shaped excavation in the medial side of the posterior wing of this element. *Dallasaurus* was recoded as ?.

(67) Surangular coronoid buttress: low, thick, about parallel to lower edge of mandible (0); or high, thin, rapidly rising anteriorly (1). A rounded dorsal edge of the surangular remains almost parallel to the ventral edge as it approaches the posterior end of the coronoid, meeting the latter element near its posteroventral edge in state 0. In state 1, the dorsal edge rises and thins anteriorly until meeting the posterior edge of the coronoid near its apex, producing a triangular posterior mandible in lateral aspect.

(68) Surangular-articular suture position: behind the condyle in lateral view (0); or at middle of glenoid on lateral edge (1); anterior to condyle (2). In state 1, there is usually an interdigitation in the dorsal part of the suture. *Aigialosaurus dalmaticus* was rescored as 2.

(69) Surangular-articular lateral suture trace: suture descends and angles or curves anteriorly (0); or is virtually straight throughout its length (1). In state 1, the suture trails from the glenoid posteriorly about halfway along the dorsolateral margin of the retroarticular process, then abruptly turns anteriorly off the edge and strikes in a straight line for the posterior end of the angular.

(70) Articular retroarticular process inflection: moderate inflection, less than 60^o^ (0); or extreme inflection, almost 90^o^ (1).

(71) Articular retroarticular process innervation foramina: no large foramina on lateral face of retroarticular process (0); or one to three large foramina present (1).

(72) Tooth surface I: teeth finely striate medially (0); or not medially striate (1). In “Russellosaurinae,” medial tooth striations are very fine and groups of tightly spaced striae are usually set apart by facets, leading to a fasciculate appearance. *Angolasaurus* was recoded as ?, *Aigialosaurus* was recoded as having state 1.

(73) Tooth surface II: teeth not coarsely textured (0); or very coarsely ornamented with bumps and ridges (1). In both species of *Globidens* and in *Prognathodon* *overtoni*, the coarse surface texture is extreme, consisting of thick pustules, and vermiform or anastomosing ridges. Teeth in *P.* *rapax* are smooth over the majority of their surface, but usually a few widely scattered, large, very long, sharp-crested vermiform ridges are present.

(74) Tooth facets: absent (0); or present (1). *Halisaurus* teeth are smoothly rounded except for the inconspicuous carinae. *Clidastes* is described in numerous places as having smooth unfaceted teeth, but many immature individuals and some larger specimens have teeth with three distinct facets on the medial faces. Adult *Tylosaurus* proriger has indistinct facets. *Mosasaurus* has taken this characteristic to the extreme. *Russellosaurus*, *Tethysaurus*, *Angolasaurus*, *Ectenosaurus*, *Platecarpus* (*P. planifrons* and *P. tympaniticus*), and *Plioplatecarpus* have been recoded as having state 0.

(75) Tooth fluting: absent (0); or present (1). In *Ectenosaurus*, and some *Platecarpus* *planifrons*, several broadly rounded vertical ridges alternate with shallow, round-bottomed grooves completely around the teeth. *Tethysaurus* was recoded as having both states 0 and 1, because grooves can be observed in larger specimens. *Angolasaurus* was recoded as having state 1.

(76) Tooth inflation: crowns of posterior marginal teeth conical, tapering throughout (0); or crowns of posterior marginal teeth swollen near the tip or above the base (1).The rear teeth of *Globidens* and *Prognathodon overtoni* are distinctly fatter than other mosasauroid teeth, but those of *P. rapax* are also swollen immediately distal to the base.

(77) Tooth carinae I: absent (0); or present but extremely weak (1); or strong and elevated (2). *Halisaurus* exhibits the minimal expression of this character (state 1) in that its marginal teeth are almost perfectly round in cross-section; the carinae are extremely thin and barely stand above the surface of the teeth.

(78) Tooth carinae serration: absent (0); or present (1).

(79) Tooth replacement mode: replacement teeth form in shallow excavations (0); or in subdental crypts (1). All mosasauroids that can be evaluated have an ‘anguimorph’ type of tooth replacement, which is to have interdental positioning of replacement teeth and resorption pits associated with each. *Angolasaurus* was recoded as ?.

(80) Atlas neural arch: notch in anterior border (0); or no notch in anterior border (1). *Dallasaurus* was recoded as ?.

(81) Atlas synapophysis: extremely reduced (0); or large and elongate (1). In state 1, a robust synapophysis extends well posteroventral to the medial articular surface for the atlas centrum, and it may be pedunculate (*Clidastes*) or with a ventral ‘skirt’ that gives it a triangular shape (*Mosasaurus*). A very small triangular synapophysis barely, if at all, extends posterior to the medial articular edge in state 0.

(82) Zygosphenes and zygantra: absent (0); or present (1). This character assesses only the presence of zygosphenes and zygantra, not their relative development. Nonfunctional and functional are considered as present. Although the outgroup we selected (*Varanus*) does not possess zygosphene and zygantra we decided to code the primitive condition as present because these structures can be observed in primitive varanoids like *Saniwa*.

(83) Zygosphene and zygantra number: present on many vertebrae (0); or present on only a few (1). As per the argument discussed for character 84 we coded the outgroup as having state 0.

(84) Hypapophyses: last hypapophysis occurs on or anterior to seventh vertebra (0); or on eight or posteriorly (1).

(85) Synapophysis height: facets for rib articulations tall and narrow on posterior cervicals and anterior trunk vertebrae (0); or facets ovoid, shorter than the centrum height on those vertebrae (1).

(86) Synapophysis length: synapophyses of middle trunk vertebrae not laterally elongate (0); or distinctly laterally elongate (1). The lateral extension of the synapophyses from the middle of the trunk is as much as 70–80% of the length of the same vertebra is represented by state 1.

(87) Synapophysis ventral extension: synapophyses extend barely or not at all below ventral margin of cervical centra (0); or some extend far below ventral margin of centrum (1). In state 1, two or more anterior cervical vertebrae have rib articulations that dip well below the centrum, causing a very deeply concave ventral margin in anterior profile.

(88) Vertebral condyle inclination: condyles of trunk vertebrae inclined (0); or condyles vertical (1).

(89) Vertebral condyle shape I: condyles of anterior-most trunk vertebrae extremely dorsoventrally depressed (0); or essentially equidimensional (1). In state 0, posterior height: width ratios of anterior trunk vertebrae are close to 2:1. In state 1, they are between to 4:3 and 1:1.

(90) Vertebral condyle shape II: condyles of posterior trunk vertebrae not higher than wide (0); or slightly compressed (1). In state 1, the posterior condylar aspect reveals outlines that appear to be higher than wide and even perhaps slightly subrectangular, due to the slight emargination for the dorsal nerve cord.

(91) Vertebral synapophysis dorsal ridge: sharp ridge absent on posterior trunk synapophyses (0); or with a sharp-edged and anteriorly precipitous ridge connecting distal synapophysis with prezygapophysis (1). In state 0, the ridge in question, if present, may be incomplete or it may be rounded across the crest with the anterior and posterior sides about equally sloping.

(92) Vertebral length proportions: cervical vertebrae distinctly shorter than longest vertebrae (0); or almost equal or are the longest (1).

(93) Presacral vertebrae number I: relatively few, 32 or less (0); or numerous, 39 or more (1). Here, presacral vertebrae are considered to be all those anterior to the first bearing an elongate transverse process.

(94) Presacral vertebrae number II: if few, then 28 or 29 (0); 30 or 31 (1).

(95) Sacral vertebrae number: two (0); or less than two (1). Numerous well preserved specimens of derived mosasauroids have failed to show any direct contact of the pelvic girdle with vertebrae in the sacral area. Certainly, no transverse processes bear any type of concave facet for the ilium, and so it is generally assumed that a ligamentous contact was established with only one transverse process. Depending on one’s perspective, it could be said that derived mosasauroids have either no or one sacral vertebra.

(96) Caudal dorsal expansion: neural spines of tail all uniformly shortened posteriorly (0); or several spines dorsally elongated behind middle of tail (1). *Dallasaurus* was recoded as ?.

(97) Haemal arch length: haemal arches about equal in length to neural arch of same vertebra (0); or length about 1.5 times greater than neural arch length (1). This ratio may be as great as 1.2:1 in state 0. Comparison is most accurate in the middle of the tail and is consistent even on those vertebrae in which the neural spines are also elongated.

(98) Haemal arch articulation: arches articulating (0); or arches fused to centra (1).

(99) Tail curvature: no structural downturn of tail (0); or tail with curved posterior portion (1).

(100) Body proportions: head and trunk shorter than or about equal to tail length (0); or head and trunk longer than tail (1).

(101) Scapula/coracoid size: both bones about equal (0); or scapula about half the size of coracoid (1). *Dallasaurus* was recoded as ?.

(102) Scapula width: no anteroposterior widening (0); or distinct fan-shaped widening (1); or extreme widening (2). In state 0, the anterior and posterior edges of the scapula encompass less than one quarter of the arc of a circle, but in state 1, the arc is increased to approximately one third. In state 2, the distal margin encompasses almost a half-circle and the anterior and posterior borders are of almost equal length.

(103) Scapula dorsal convexity: if scapula widened, dorsal margin very convex (0); or broadly convex (1). In state 0, the anteroposterior dimension is almost the same as the proximodistal dimension. In state 1, the anteroposterior dimension is much larger.

(104) Scapula posterior emargination: posterior border of bone gently concave (0); or deeply concave (1). In state 1, there is a deeply arcuate emargination on the posterior scapular border, just dorsal to the glenoid. It is immediately bounded dorsally by a corner, which begins a straight-edged segment that continues to the dorsal margin.

(105) Scapula-coracoid suture: unfused scapula-coracoid contact has interdigitate suture anteriorly (0); or apposing surfaces without interdigitation (1). *Dallasaurus* was recoded as ?.

(106) Coracoid neck elongation: neck rapidly tapering from medial corners to a relatively broad base (0); or neck gradually tapering to a relatively narrow base (1); coracoid neck absent (2). In state 1, this character describes an outline of the bone, which is nearly symmetrical and gracefully fan-shaped, with gently concave, nearly equidistant sides.

(107) Coracoid anterior emargination: present (0); or absent (1).

(108) Humerus length: humerus distinctly elongate, about three or more times longer than distal width (0); or greatly shortened, about 1.5 to 2 times longer than distal width (1); or length and distal width virtually equal (2); or distal width slightly greater than length (3).

(109) Humerus postglenoid process: absent or very small (0); or distinctly enlarged (1).

(110) Humerus glenoid condyle: if present, condyle gently domed and elongate, ovoid in proximal view (0); or condyle saddle-shaped, subtriangular in proximal view and depressed (1); or condyle highly domed or protuberant and short ovoid to almost round in proximal view (2). In some taxa, the condylar surfaces of the limbs were finished in thick cartilage and there was no bony surface of the condyle to be preserved. This condition is scored as not represented. In some taxa, the glenoid condyle extends more proximally than does the postglenoid process (state 2), and it is not ovoid as state 0. *Dallasaurus* was recoded as having state 0.

(111) Humerus deltopectoral crest: crest undivided (0); or split into two separate insertional areas (1). In state 1, the deltoid crest occupies an anterolateral or anterior position confluent with the glenoid condyle, while the pectoral crest occupies a medial or anteromedial area that may or may not be confluent with the glenoid condyle. The deltoid crest is often quite short, broad, and indistinct, being easily erased by degradational taphonomic processes.

(112) Humerus pectoral crest: located anteriorly (0); or medially (1). In state 1, the pectoral crest is located near the middle of the flexor (or medial) side on the proximal end of the bone.

(113) Humerus ectepicondylar groove: groove or foramen present on distolateral edge (0); or absent (1).

(114) Humerus ectepicondyle: absent (0); or present as a prominence (1). A radial tuberosity is reduced in size in *Prognathodon*, but very elongated in *Plesiotylosaurus*. *Tethysaurus* was recoded as having state 0.

(115) Humerus entepicondyle: absent (0); or present as a prominence (1). The ulnar tuberosity protrudes posteriorly and medially from the posterodistal corner of the bone immediately proximal to the ulnar facet, causing a substantial dilation of the posterodistal corner of the humerus. *Tethysaurus* was recoded as having state 0.

(116) Radius shape: radius not expanded anterodistally (0); or slightly expanded (1); or broadly expanded (2).

(117) Ulna contact with centrale: broad ulnare prevents contact (0); or ulna contacts centrale (1). In state 1, the ulnare is omitted from the border of the antebrachial foramen. There is usually a well-developed faceted articulation between the ulna and the centrale (or intermedium, as used by Russell, 1967).

(118) Radiale size: large and broad (0); or small to absent (1).

(119) Carpal reduction: carpals number six or more (0); or five or less (1).

(120) Pisiform: present (0); or absent (1).

(121) Metacarpal I expansion: spindle-shaped, elongate (0); or broadly expanded (1). The broad expansion is also associated with an anteroproximal overhanging crest in every case observed.

(122) Phalanx shape: phalanges elongate, spindle-shaped (0); or blocky, hourglass-shaped (1). *Mosasaurus* and *Plotosaurus* have phalanges that are slightly compressed and anteroposteriorly expanded on both ends. *Dallasaurus* was recoded as ?.

(123) Ilium crest: crest blade-like, articulates with sacral ribs (0); or elongate, cylindrical, does not articulate with sacral ribs (1).

(124) Ilium acetabular area: arcuate ridge supertending acetabulum (0); or acetabulum set into broad, short ‘V’-shaped notch (1). The primitive ilium has the acetabulum impressed on the lateral wall of the bone, with a long narrow crest anterodorsally as the only surrounding topographic feature. In state 1, the acetabular area is set into a short, broadly ‘V’-shaped depression that tapers dorsally. The lateral walls of the ilium are therefore distinctly higher than the rim of the acetabulum.

(125) Pubic tubercle condition: tubercle an elongate protuberance located closer to the midlength of the shaft (0); or a thin semicircular crest-like blade located close to the acetabulum (1).

(126) Ischiadic tubercle size: elongate (0); or short (1). In state 0, the tubercle is as long as the shaft of the ischium is wide, but it is only a short narrow spur in state 1.

(127) Astragalus: notched emargination for the crural foramen, without pedunculate fibular articulation (0); or without notch, pedunculate fibular articulation present (1). For state 0, the tibia and fibula are of equal length about the crural foramen and the astragalus contacts both to about the same degree. The form of the latter element is symmetrical and subcircular with a sharp proximal notch. In state 1, the outline of the element is basically reniform and the tibial articulation is on the same line as the crural emargination. The fibula is also shortened and its contact with the astragalus is narrow.

(128) Appendicular epiphyses: formed from ossified cartilage (0); or from thick unossified cartilage (1); or epiphyses missing or extremely thin (2). Ends of the limb bones show distinct vascularization and rugose surfaces indicating an apparently thick non-vascularized, unossified cartilage cap. Extremely smooth articular surfaces suggest the epiphyses were excessively thin or perhaps even lost.

(129) Hyperphalangy: absent (0); or present (1). Hyperphalangy is defined as presence of one or more extra phalanges as compared to the primitive amniote formula of 2-3-4-5-3.

(130) Posterior thoracic vertebra: not markedly longer than anterior thoracic vertebrae (0); or are markedly longer (1).

(131) Ectopterygoid process of pterygoid: distal portion of process not offset anterolaterally and/or lacking longitudinal grooves and ridges (0); distal portion of process is offset anterolaterally and bears longitudinal grooves and ridges (1).
